# 1,2,4,5‐Tetrakis(tetramethylguanidino)‐3,6‐diethynyl‐benzenes: Fluorescent Probes, Redox‐Active Ligands and Strong Organic Electron Donors

**DOI:** 10.1002/chem.202001557

**Published:** 2020-07-10

**Authors:** Conrad Wagner, Franka Kreis, Dennis Popp, Olaf Hübner, Elisabeth Kaifer, Hans‐Jörg Himmel

**Affiliations:** ^1^ Anorganisch-Chemisches Institut Ruprecht-Karls-Universität Heidelberg Im Neuenheimer Feld 270 69120 Heidelberg Germany

**Keywords:** copper, guanidine, organic electron donors, redox chemistry, redox-active ligand

## Abstract

In this work, the change of reactivity induced by the introduction of two *para*‐ethynyl substituents (CCSi(*i*Pr)_3_ or CCH) to the organic electron‐donor 1,2,4,5‐tetrakis(tetramethylguanidino)‐benzene is evaluated. The redox‐properties and redox‐state dependent fluorescence are evaluated, and dinuclear Cu^I^ and Cu^II^ complexes synthesized. The Lewis‐acidic B(C_6_F_5_)_3_ substitutes the proton of the ethynyl −CCH groups to give new anionic −CCB(C_6_F_5_)_3_^−^ substituents, leading eventually to a novel dianionic strong electron donor in its diprotonated form. Its two‐electron oxidation with dioxygen in the presence of a copper catalyst yields the first redox‐active guanidine that is neutral (instead of cationic) in its oxidized form.

## Graphical abstract

**The diverse chemistry** of redox‐active guanidines is enriched by compounds with alkynyl groups, showing redox‐state‐dependent fluorescence, and allowing the synthesis of the first redox‐active guanidine that is neutral in its oxidized form (see figure).

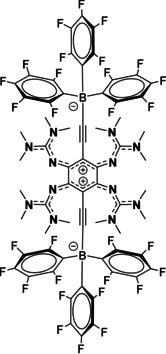

## Introduction

Redox‐active organic molecules are used in numerous applications,^1^ for example, in electrochromic devices,^2^, ^3^, ^4^ in redox reactions and proton‐coupled electron transfer,^5^, ^6^, ^7^, ^8^ and as redox‐active ligands in coordination chemistry.^9^, ^10^, ^11^, ^12^ The interest in these compounds is largely stimulated by the possibility to rationally tune the redox properties and further desired features by the rich arsenal of organic synthesis strategies. Quinones are particularly well‐studied redox‐active compounds, and are found in numerous applications. For example, they are used as stoichiometric reagents (often in combination with strong acids) in a number of (Scholl‐type) aryl‐aryl coupling reactions,^13^, ^14^ and as redox catalysts for example, for the oxidation of amines and alcohols.^15^, ^16^ The anthraquinone process for hydrogen peroxide production is a large‐scale industrial application of quinones.^17^, ^18^ They are also applied in some steps of the synthesis of pharmaceuticals and drugs.^19^ Moreover, *o*‐quinones/catecholates are prime examples for redox‐active ligands that are currently intensively used in coordination chemistry. Finally, their applications in organic redox‐flow batteries is currently studied.^20^, ^21^, ^22^, ^23^ Generally, quinones are neutral in their oxidized form and dianionic in their reduced form.

**Scheme 1:**
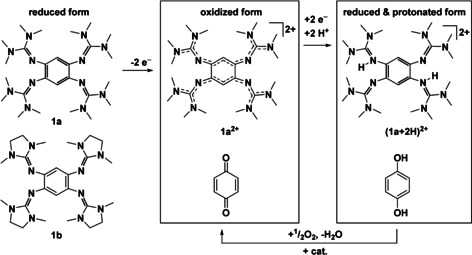
Lewis structures of GFA **1 a** and **1 b** and oxidized and reduced, protonated states of **1 a** that could be used in PCET (2 e−/2 H^+^) reactions. The relevant compounds are dications, in contrast to the neutral benzoquinone and dihydrobenzoquinone.

Our group developed guanidino‐functionalized aromatic compounds (GFAs) as a powerful class of redox‐active molecules,^11^, ^24^, ^25^, ^26^, ^27^ and demonstrated the use of GFAs in several fields of modern research. Hence, GFAs like the redox‐active guanidines **1 a** or **1 b** (Scheme 1)^28^ were found to be superior reagents in proton coupled electron transfer reactions,^24^, ^29^, ^30^, ^31^, ^32^ allowing a variety of aryl‐aryl coupling reactions that were previously only feasible with quinones such as DDQ or chloranil (CA).^32^ They could also be used in materials, for example, semiconducting devices^33^ or „low‐dimensional perovskites“.^34^ Finally, GFAs like **1 a** are versatile redox‐active ligands in late‐transition metal complexes, establishing stable ligand‐metal bonding in three redox states of the ligand (neutral, radical monocationic and dicationic).^25^, ^35^, ^36^, ^37^ In this context it should be noted that complexes with guanidine or guanidinate ligands^38^, ^39^, ^40^, ^41^ as well as other *N*‐heterocyclic imino (NHI) ligands/substituents^42^, ^43^, ^44^ are intensively studied.

In several works we described intramolecular (reversible) electron‐transfer processes between GFA ligands and metal atoms in mono‐ and dinuclear copper complexes,^11^, ^45^, ^46^, ^47^ including the first dinuclear copper complexes showing reversible, thermally stimulated redox isomerism (also denoted valence tautomerism).^47^

Starting with the archetypical compounds **1 a** and **1 b**, several derivatives were obtained by substituting the two remaining aromatic protons (e.g. by halides,^26^, ^48^ nitro^48^ or even additional guanidino groups^27^), or by modifying the guanidino groups.^49^, ^50^ These substitutions affect the redox properties as well as the optical properties.^24^

**Figure 1:**
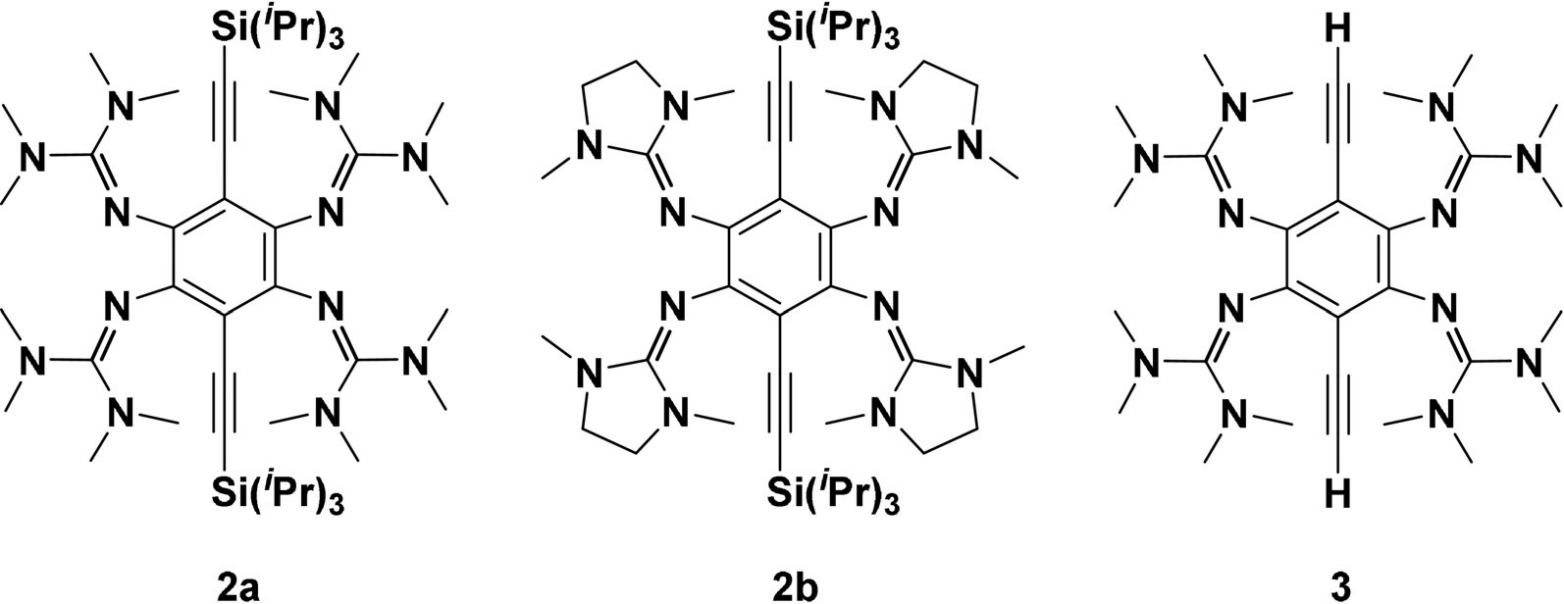
Lewis representations of the three 1,2,4,5‐tetrakis(tetramethylguanidino)‐3,6‐diethynyl‐benzene compounds studied in this work.

Herein we report on the synthesis and the chemistry of compounds, in which the two remaining aromatic protons of **1 a**/**1 b** are substituted by ethynyl groups. Figure 1 shows the Lewis structures of the three compounds **2 a**, **2 b** and **3** studied in this work. The synthesis of **2 a** was described in a preliminary work.^51^ As detailed in the following, the peculiarities of these three compounds are the redox‐state dependent fluorescence, and the additional reactivity inscribed by the ethynyl groups (especially for compound **3**). Moreover, the first dinuclear metal complexes of **2 a** and **3** are synthesized and analysed.

## Results and Discussion

### Synthesis and characterization of 1,2,4,5‐tetrakis(tetramethylguanidino)‐3,6‐diethynyl‐benzenes

**Scheme 2:**
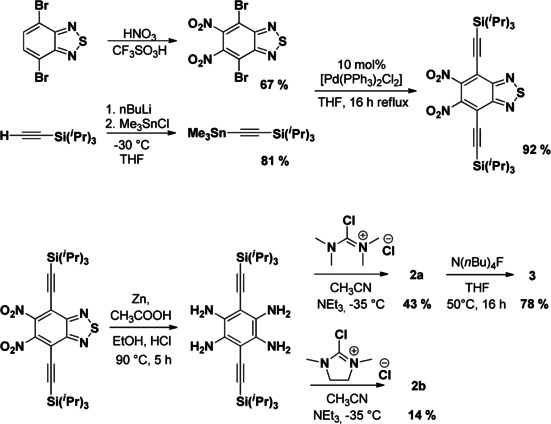
Synthesis of the **2 a**,^51^ **2 b** and **3**. The yields refer to the isolated, pure compounds.

The synthesis of the three compounds (see Scheme 2) commences with 4,7‐dibromo‐2,1,3‐benzothiadiazole. Conversion to 5,6‐dinitro‐4,7‐bis[2‐[tris(1‐methylethyl)silyl]ethynyl]‐2,1,3‐benzothiadiazole is followed by reduction to give 1,2,4,5‐tetra(amino)‐3,6‐bis‐[(triisopropylsilyl)ethynyl]benzene. Reaction with chloro‐N,N,N’,N’‐tetramethyl‐formamidinium‐chloride leads to **2 a** (43 % isolated yield)^51^ and reaction with 2‐chloro‐1,3‐dimethyl‐4,5‐dihydro‐1*H*‐imidazolium‐chloride leads to **2 b** (14 % isolated yield). The low isolated yield of **2 b** is due to its relatively high solubility in organic solvents that hampers its isolation by precipitation. Removal of the two silyl groups from compound **2 a** is achieved with tetrabutylammonium fluoride in THF, yielding pure compound **3** in good yield (78 %). The addition of an extra proton source is not required. The presence of terminal alkynes was evidenced by NMR and IR spectroscopy. Hence, the two protons of the alkyne groups show at *δ*=3.05 ppm in the ^1^H NMR spectrum. In the IR spectrum, sharp absorptions at 3260 and at 2084 cm^−1^ are assigned to the alkyne stretching modes *ν*(C−H) and *ν*(C≡C), respectively.

Interestingly, the three compounds differ distinctly in their solubility. Compound **2 a** is soluble in THF, but much less soluble in Et_2_O or toluene. It is completely insoluble in more polar solvents such as Me_2_CO or CH_3_CN. It is highly soluble in CH_2_Cl_2_, but decomposes in this solvent within hours to unknown products. By contrast, **2 b** is much more soluble in CH_3_CN or toluene. Compound **3** is generally barely soluble in standard organic solvents, and seems to decompose within hours in toluene and especially in CH_2_Cl_2_ solution. Please note that cyclic voltammetry studies in CH_2_Cl_2_ are still possible (see below), but no reactions of these compounds in this solvent could be carried out. For comparison, compounds **1 a** and **1 b** are stable and soluble in CH_3_CN and CH_2_Cl_2_ solutions. The differences in solubility and stability limit a comparison of the reactivity of the three compounds.

**Figure 2:**
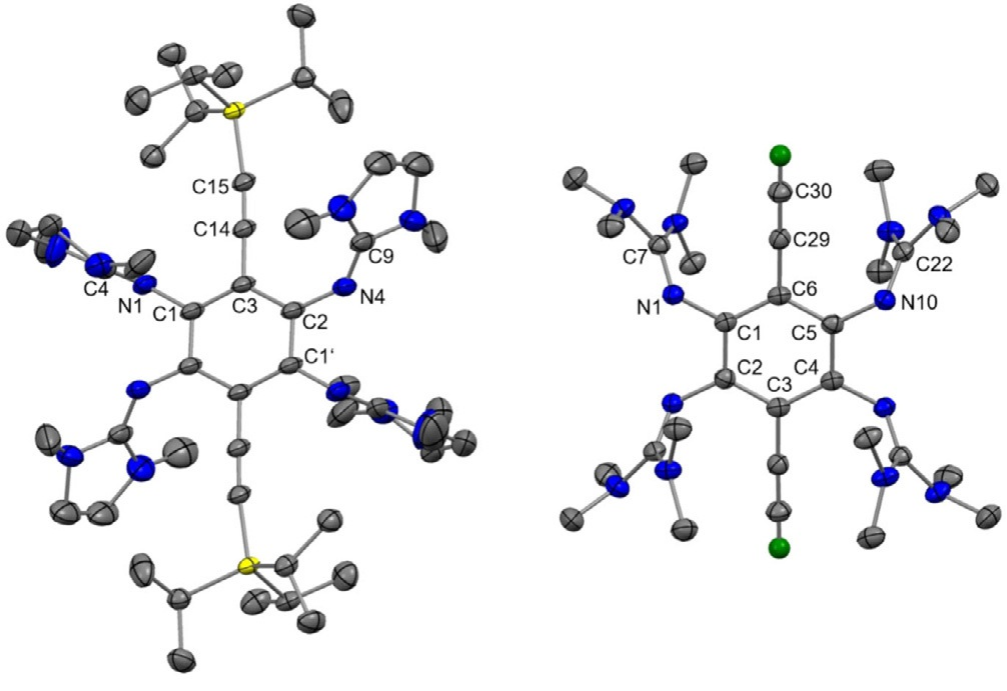
Illustration of the solid‐state structures of **2 b** and **3**. Displacement ellipsoids drawn at the 50 % probability level. Hydrogen atoms bound to carbon were input at calculated positions and refined with a riding model. Ethynyl hydrogens in green, all other hydrogens omitted. Selected bond lengths are included in Table 1.

Table 1 compares some bond parameters for **2 a**,^51^ **2 b** and **3**, and the solid‐state structures of **2 b** and **3** are visualized in Figure 2. In similarity to the structures of other GFAs, the CN_3_ planes of the guanidino groups are highly twisted with respect to the central aromatic C_6_ ring plane (see the analysis of this issue in ref. 52). Due to this preferred conformation, there is no steric strain in the molecule. The imino N=C bond lengths (N1‐C4/N4‐C9 in **2 a**/**2 b** and N1‐C7/N10‐C22 in **3**) are similar for all compounds (shortest and longest bonds of 1.283(3) and 1.294(4) Å, respectively), and fall in a typical range for N=C double bonds in neutral guanidines.^24^ These bonds are very sensitive to changes in the electronic structure, and are elongated significantly upon protonation, metal coordination or oxidation (see discussion below).

**Table 1:** Comparison of selected bond lengths (in Å) for the three new neutral GFAs and the compounds obtained upon two‐electron oxidation.

bond	**2 a** 51	**2 a** ^2+^(PF_6_^−^)_2_	**2 b**	**3**	**3** ^2+^(PF_6_^−^)_2_
N1−C1 N4−C2	1.409(4) 1.411(4)	1.341(2) 1.298(2)	1.413(2) 1.401(3)	1.414(2) 1.408(2)^N10−C5^	1.336(1) 1.296(1)
N1−C4 N4−C9	1.294(4) 1.284(4)	1.334(2) 1.365(2)	1.283(3) 1.286(3)	1.290(2)^N1−C7^ 1.292(2)^N10−C22^	1.342(1) 1.372(1)
C1−C2	1.408(4)	1.490(2)	1.408(2)	1.402(2)	1.498(1)
C1−C3	1.408(4)	1.385(2)	1.406(3)	1.412(2)^C1−C6^	1.389(1)
C2−C3	1.415(4)	1.446(2)	1.415(3)	1.412(2)^C6−C5^	1.443(1)
C3−C14	1.436(4)	1.431(2)	1.441(2)	1.442(2)^C6−C29^	1.425(2)
C14−C15	1.208(5)	1.209(3)	1.205(2)	1.195(2)^C29−C30^	1.199(1)

**Figure 3:**
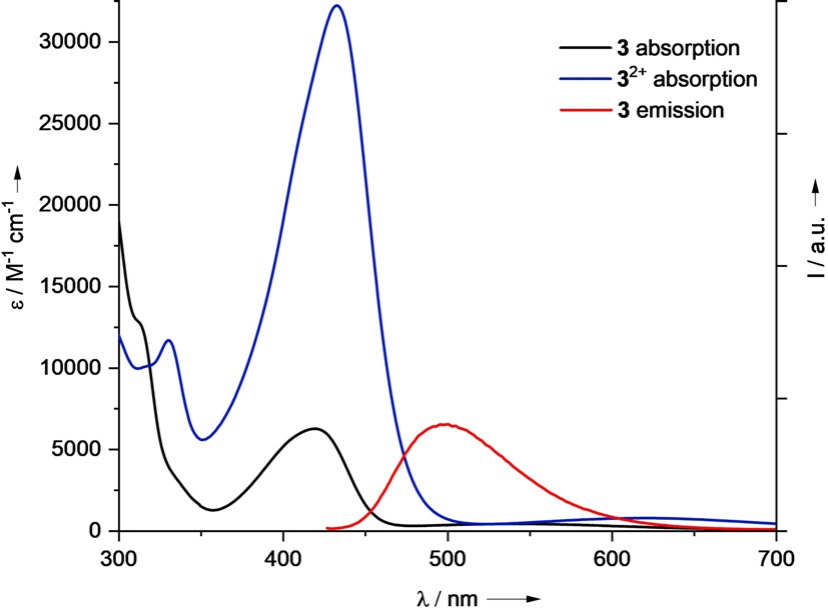
UV‐vis and luminescence spectra recorded for compound **3** (toluene) and **3** ^2+^(PF_6_^−^)_2_ (acetonitrile).

Next, we inspected the optical properties of the three compounds. Due to the huge difference in solubility, the spectra had to be recorded in different solvents. The optical properties of all discussed compounds are collected in Table 2. In the electronic absorption spectra, all three compounds **2 a**, **2 b** and **3** display one band in the visible region, with maxima of absorption at 433 (**2 a** in THF), 429 (**2 b** in THF) and 420 (**3** in toluene) nm (see Figure 3 for compound **3** in toluene). The extinction coefficient is only slightly higher for **2 a** than for **2 b** (by ca. 10 %), but significantly higher than for **3**. All three compounds show relatively strong fluorescence (maximum of emission at 502 (**2 a**), 504 (**2 b**) and 500 (**3)** nm, see Figure 3), in difference to the fluorescent‐silent compounds **1 a** and **1 b**. The quantum yields increase in the row **3** (*Φ*=12 %) <**2 a** (*Φ*=18 %) <**2 b** (*Φ*=31 %). The more rigid guanidino groups in **2 b** might be responsible for the remarkable difference in the quantum yield between **2 a** and **2 b** (both in THF solution). In this context it is worth noting that the quantum yield of fluorescence of **2 a** in solution massively increases upon decrease of the temperature.^51^

**Table 2:** Comparison of the optical properties for several compounds: **2 a**, (**2 a**+2 H)^2+^(PF_6_^−^)_2_, (**2 a**)^2+^(PF_6_^−^)_2_, **2 b** and **5** in THF, (**2 a**+4 H)^4+^(Cl^−^)_4_ and **3** ^2+^(PF_6_^−^)_2_ in CH_3_CN, **3** in toluene, **4** in CH_2_Cl_2_ (fluorescence quantum yield *Φ*, life time *τ*).

Compound	*λ*_max, abs._ [nm] (*ϵ* [m ^−1^ cm^−1^])	*λ*_max, em._ [nm]	*Φ*	*τ* [ns]
**2 a**	332 (32 200), 433 (10 900)	502	0.18	2.3
(**2 a**+2H)^2+^(PF_6_^−^)_2_	293 (82 100), 419 (13 300)	508	0.31	3.9
(**2 a**+4H)^4+^(Cl^−^)_4_	279 (47 000), 392 (4800)	477	0.33	5.3
(**2 a**)^2+^(PF_6_^−^)_2_	346 (21 200), 446 (32 100)	–	–	–
**2 b**	330 (30 000), 429 (10 000)	504	0.31	*a*
**3**	313 (12 500), 420 (6200)	500	0.12	*a*
**3** ^2+^(PF_6_^−^)_2_	330 (11 600), 433 (32 300)	–	–	–
**4**	299 (23 600), 401 (8300)	445	*b*	*b*
**5**	323 (23 800), 455 (29 600)	–	–	–

[a] Not determined. [b] Fluorescence signal too weak.

**Figure 4:**
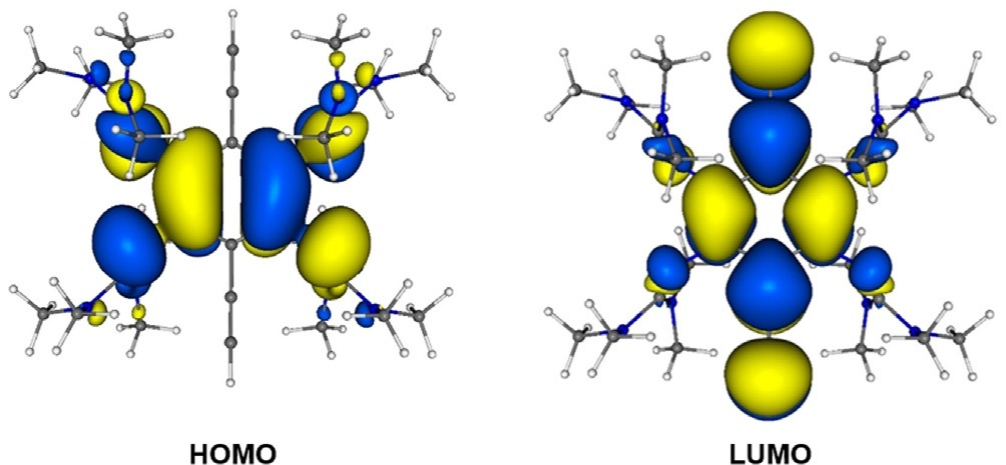
Isodensity plots of HOMO and LUMO for **3**. The HOMO—LUMO transition excites electrons from one π‐system (involving the guanidino groups) to an orthogonal π‐systems (involving the ethynyl groups), offering a qualitative explanation for the fluorescence properties. Contour values for the isodensity plots are ±0.02 Bohr^(−3/2)^.

Quantum‐chemical calculations (B3LYP/def2‐TZVP) were carried out to get information about the nature of the electronic transition. The calculated lowest‐energy electronic transition (TD‐DFT calculation) is in excellent agreement with the experimental results (observed: 433 nm for **2 a** and 420 nm for **3**; calcd 429 nm for **2 a** 51 and 403 nm for **3**), and can safely be assigned to the HOMO→LUMO transition (see Supporting Information, Figures S47 and S48). The C_6_ ring and the guanidino groups, but not the ethynyl groups contribute to the HOMO orbital. By contrast, the LUMO is localized on the C_6_ ring and the ethynyl groups, and the guanidino groups contribute only marginally (see Figure 4). Hence, in the HOMO→LUMO transition an electron is excited from one π‐system to an orthogonal π‐system, like in typical cross‐conjugated cruciform fluorophores.^53^

### Redox properties

**Figure 5:**
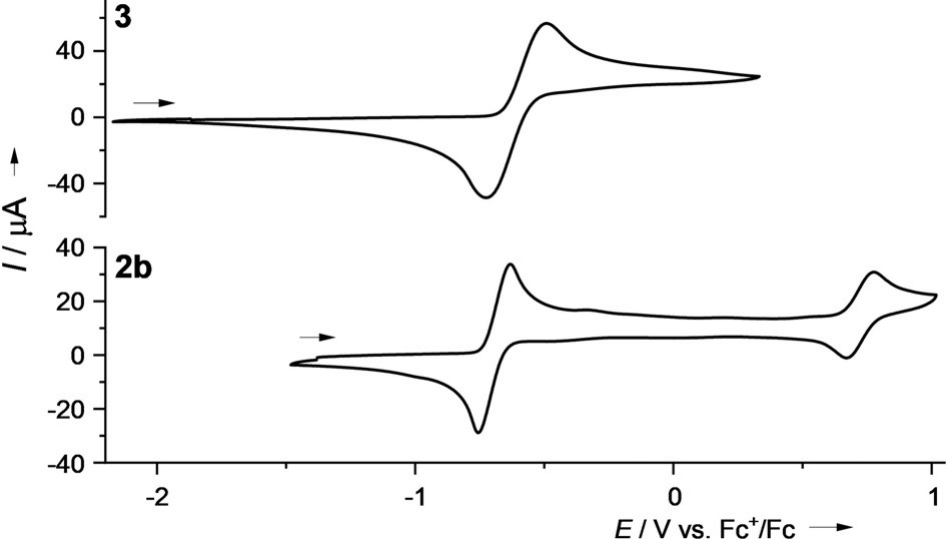
CV curves for **2 b** and **3** in CH_2_Cl_2_ solution (1 mm) (with 0.1 m N(*n*Bu)_4_PF_6_ as supporting electrolyte, Ag/AgCl reference electrode, 100 mV s^−1^ scan rate). Potentials given vs. the redox‐couple ferrocenium/ferrocene (Fc^+^/Fc).

The redox properties are first analysed in electrochemical studies. In Table 3, the redox potentials obtained from cyclic voltammetry (CV) for the compounds **2 a**, **2 b** and **3** are compared with those of **1 a** and **1 b**. In all cases a quasi‐reversible two‐electron redox process is observed. At high potentials, a one‐electron redox process follows, leading eventually to the GFA trication. The alkynyl groups shift the redox potential to slightly higher values. This shift is larger for the CCH groups than for the CCSi(*i*Pr)_3_ groups. Compound **3** in CH_2_Cl_2_ solution (see Figure 5) shows the quasi‐reversible two‐electron redox process, assigned to the redox couple **3** ^2+^/**3**, with the highest potential (*E*_1/2_=−0.61 V, *E*_ox_=−0.49 V) of the tetrakis‐guanidine compounds studied herein. Another reversible one‐electron process which is usually observed for GFAs (GFA^2+^/GFA^**⋅**3+^), is also expected for **3**. However, the potential window in dichloromethane and the one‐electron process seem to be in proximity to each other (see Supporting Information, Figure S11). Therefore, it cannot be assigned properly, yet the value is estimated at *E*_ox_∼0.73 V. Please note that although compound **3** decomposes slowly in CH_2_Cl_2_ solutions, its lifetime is sufficiently high for cyclic voltammetry studies.

**Table 3:** Potentials (*E*_ox_ and *E*_1/2_, both given relative to Fc^+^/Fc, 100 mV s^‐1^ scan rate) from CV measurements in CH_2_Cl_2_ (N(*n*Bu)_4_PF_6_ as supporting electrolyte, Ag/AgCl reference electrode).

Redox couple		**1 a**	**1 b**	**2 a**	**2 b**	**3**
GFA/GFA^2+^	*E*_ox_ [V]	−0.62	−0.74	−0.60	−0.63	−0.49
	*E*_1/2_ [V]	−0.70	−0.79	−0.65	−0.69	−0.61
GFA^2+^/GFA^3+^	*E*_ox_ [V]	0.64	0.70	0.80	0.77	∼0.73 V
	*E*_1/2_ [V]	0.60	0.66	0.76	0.72

**Scheme 3:**
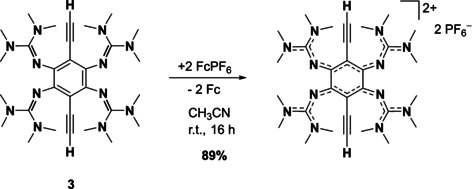
Two‐electron oxidation of **3** with ferrocenium hexafluorophosphate to give the salt **3** ^2+^(PF_6_^−^)_2_.

Motivated by the results of the cyclic voltammetry measurements, we reacted compound **3** with oxidizing reagents. Reaction of **3** with two equivalents of ferrocenium hexafluorophosphate in acetonitrile at room temperature indeed leads to clean two‐electron oxidation (Scheme 3). The product salt **3** ^2+^(PF_6_^−^)_2_, obtained in 89 % isolated yield, can be re‐crystallized by slow diffusion of diethyl ether into an acetonitrile solution. In the UV‐vis spectrum, a small bathochromic shift of the lowest‐energy absorption from *λ*_max_=420 nm to 433 nm upon oxidation is measured (see Table 2). Interestingly, this small shift is accompanied by a massive increase of the extinction coefficient (by a factor of 5.2). Moreover, oxidation completely extinguishes the fluorescence signal.

As already mentioned, compound **3** consists of two cross‐conjugated π‐systems. The donor π‐system, being the HOMO of the neutral compound, involves the aromatic ring and the guanidino groups. The acceptor π‐system, being the LUMO of the neutral compound, involves the aromatic ring and the alkynyl groups. Hence the compound could be described as a cross‐conjugated cruciform chromophore. For the dication **3** ^2+^, the LUMO is localized on the central C_6_ ring and the guanidino groups (see Supporting Information, Figure S48), in similarity to the HOMO of the neutral compound. However, the HOMO (a_g_ symmetric) and HOMO‐1 (a_u_ symmetric) of **3** ^2+^ are centred on the alkynyl groups, the central C_6_ ring, and the guanidino groups. For **3**, the lowest‐energetic electronic excitation (calculated at 402.7 nm) is a pure HOMO→LUMO transition. According to TD‐DFT (B3LYP/def2‐TZVP), the HOMO→LUMO transition of **3** ^2+^ (calculated at 675.9 nm) is symmetry forbidden, since both orbitals exhibit a_g_ symmetry. Thus, an electronic excitation with high HOMO‐1 → LUMO character (77.5 %), calculated at 439.2 nm, is assigned to the observed band at 433 nm (see Supporting Information, Figure S47). The distinct changes of the electronic excitations are responsible for the extinction of fluorescence upon oxidation of **3**.

Hence compound **3** shows distinct redox‐state dependent fluorescence, meaning that the fluorescence signal could be used as a probe for its redox state.

**Figure 6:**
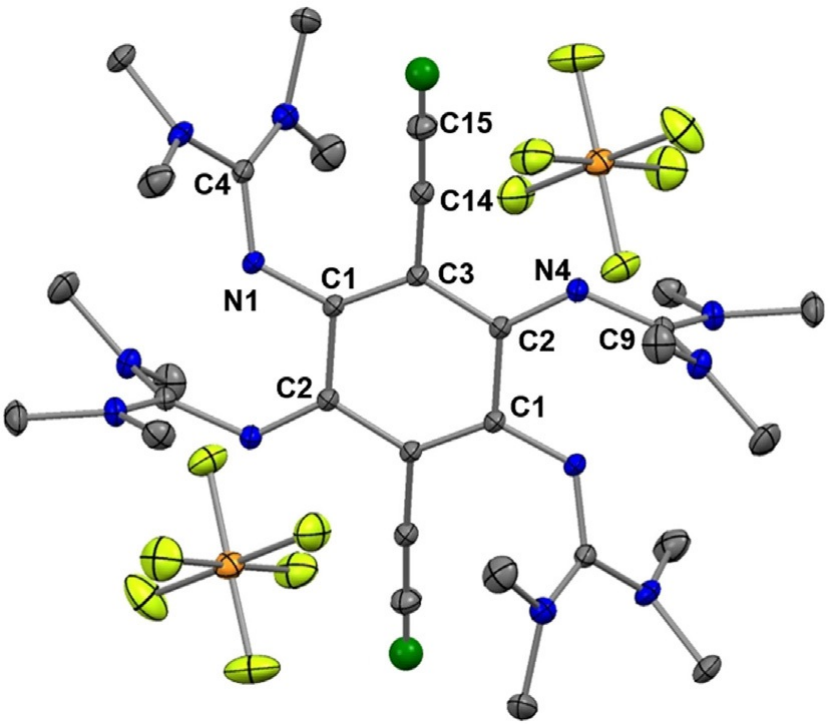
Illustration of the structure of **3** ^2+^(PF_6_^−^)_2_ in the solid state. Displacement ellipsoids drawn at the 50 % probability level. Hydrogen atoms bound to carbon were input at calculated positions and refined with a riding model. Ethynyl hydrogens in green, all other hydrogens omitted. Selected bond lengths are included in Table 1.

Figure 6 illustrates the solid‐state structure of **3** ^2+^(PF_6_^−^)_2_, and some structural parameters are included in Table 1. The significant differences between the C−C bond distances in the C_6_ ring (C1‐C2 1.498(1) Å, C1‐C3 1.389(1) Å and C2‐C3’ 1.443(1) Å) signal loss of aromaticity. The imino N=C bond distances of neutral **3** (1.290(2)/1.292(2) Å) are elongated to 1.342(1)/1.372(1) Å in **3** ^2+^(PF_6_^−^)_2_. After oxidation, the C1−N1/C2−N4 bond distances are shorter than the N1‐C4/N4‐C9 bond distances. Although aromaticity is removed, the central C_6_ ring remains planar, in line with the absence of steric strain in the compound.

### Coordination chemistry

**Scheme 4:**
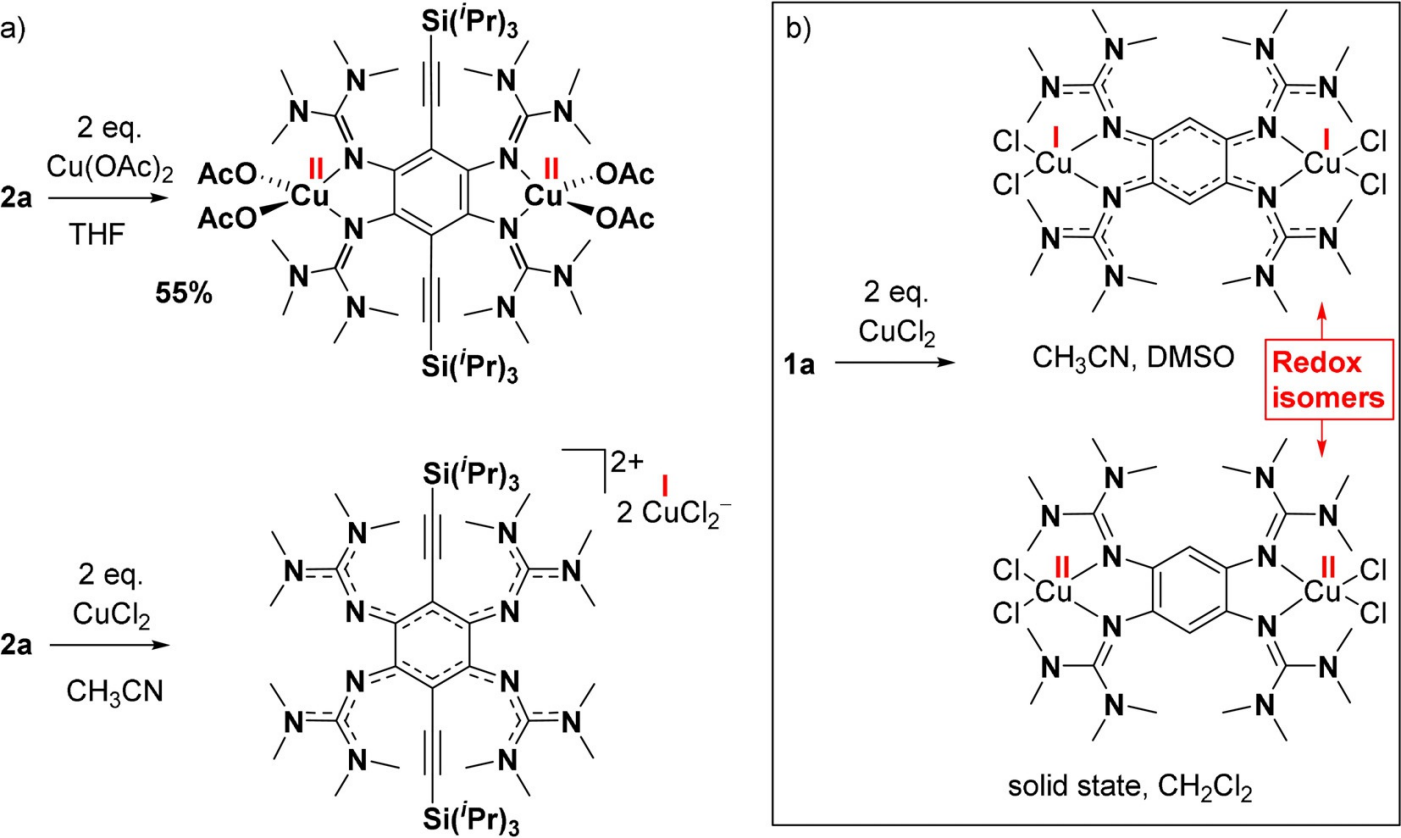
a) Different outcome of the reactions of **2 a** with one of the two Cu^II^ compounds CuCl_2_ and Cu(OAc)_2_. b) Reaction of **1 a** with 2 equiv. of CuCl_2_ (see ref. 46 for details).

**Figure 7:**
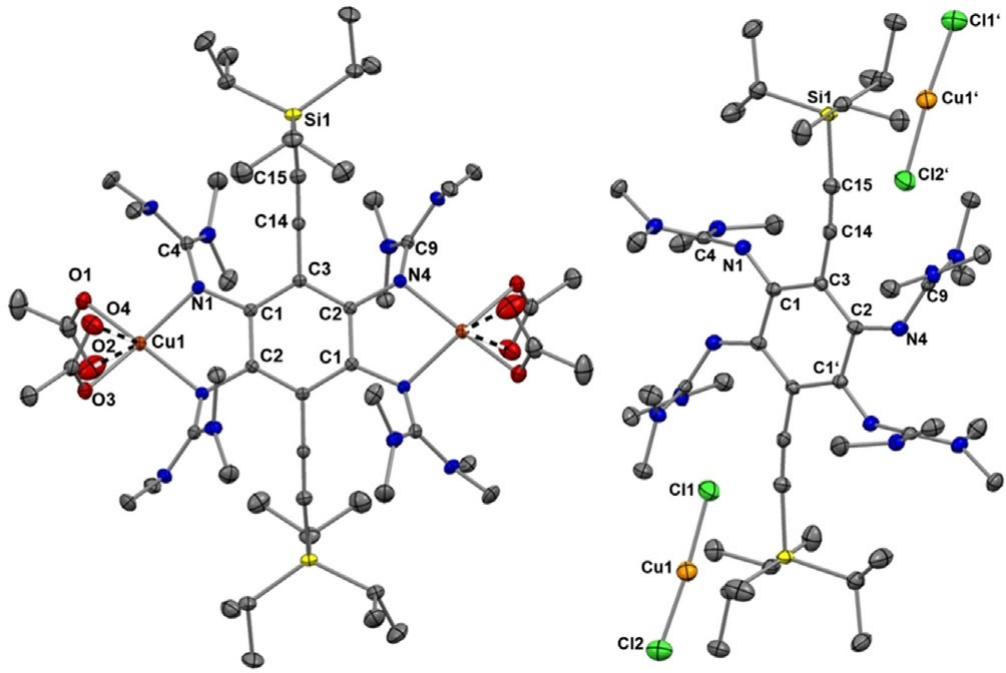
Illustration of the solid‐state structures of the complex [**2 a**{Cu(OAc)_2_}_2_] (left) and the salt (**2 a**)^2+^(CuCl_2_^−^)_2_. Displacement ellipsoids drawn at the 50 % probability level. All hydrogen atoms omitted.

Reactions of GFA **2 a** with Cu^II^ compounds could lead to electron transfer and/or formation of dinuclear copper complexes. We found different products for reactions with CuCl_2_ and Cu(OAc)_2_ (Scheme 4). With Cu(OAc)_2_ in THF solution, the paramagnetic dinuclear Cu^II^ complex [**2 a**{Cu(OAc)_2_}_2_] is formed, with a neutral ligand unit. The complex is isolated in a yield of 55 %. The UV‐vis spectrum of a THF solution displays a band at 438 nm, with a miniscule bathochromic shift with respect to free **2 a**. The fluorescence is completely extinguished upon copper coordination. The solid state structure, as derived from XRD analysis of crystals grown from a saturated CH_3_CN solution, is displayed in Figure 7. Each copper atom binds to four ligand atoms and in addition interacts weakly with two further oxygen atoms of the acetate ligands (see Table 4). As expected, the imino N=C bond distances of **2 a** are considerably elongated upon complexation (from 1.294(4)/1.284(4) Å in free **2 a** to 1.344(1)/1.345(1) Å in [**2 a**{Cu(OAc)_2_}_2_], see Table 4), in line with significant π‐contributions to the metal‐ligand bonding.^54^ The cyclic voltammogram of [**2 a**{Cu(OAc)_2_}_2_] shows several oxidation waves (at −0.30, −0.16 and +0.13 V), and an intense reduction wave at −0.67 V (see Supporting Information, Figure S19). The redox processes responsible for these waves are clearly not reversible. Most likely, oxidation initiates decomposition of the complex. This behaviour is in stark contrast to that of complexes [**1 a**{M(OAc)_2_}_2_] (M=Cu, Ni or Pd), that could be reversibly oxidized in two one‐electron steps, allowing the isolation of salts of the monocation [**1 a**{M(OAc)_2_}_2_]^+^ (with radical monocationic **1 a^⋅^** ^+^ units) and the dication [**1 a**{M(OAc)_2_}_2_]^2+^ (with dicationic **1 a** ^2+^ units).^35^, ^36^

**Table 4:** Comparison of selected bond lengths (in Å) for the copper‐containing compounds synthesized and characterized in this work.

Bond	[**2 a**(CuI)_2_]	**2 a** ^2+^(CuCl_2_^‐^)_2_	[**2 a**(CuOAc)_2_]	[**3**(CuI)_2_]	[**3**(CuOAc)_2_]
N1−C1 N4−C2	1.411(3) 1.405(3)	1.304(3) 1.315(3)	1.407(1) 1.407(1)	1.410(7) 1.414(7)	1.403(2) 1.409(2)
N1−C4 N4−C9	1.326(3) 1.327(3)	1.352(3) 1.366(2)	1.344(1) 1.345(1)	1.335(7) 1.328(7)	1.340(3) 1.350(3)
C1−C2	1.399(3)	1.506(3)	1.403(1)	1.414(7)	1.402(3)
C1−C3	1.418(3)	1.428(3)	1.417(1)	1.413(7)	1.411(3)
C2−C3	1.420(3)	1.407(3)	1.418(1)	1.406(7)	1.414(3)
C3−C14	1.438(3)	1.433(3)	1.432(1)	1.445(7)	1.436(3)
C14−C15	1.208(3)	1.218(3)	1.212(2)	1.191(8)	1.192(3)
N1−Cu1	2.028(2)	–	2.000(1)	2.022(4)	1.997(2)
N4−Cu1	2.039(2)	–	1.988(1)	2.026(4)	1.984(2)
Cu1−I1	2.431(1)	–	–	2.450(1)	–
Cu1−O1	–	–	1.946(1)	–	1.976(2)
Cu1−O2			2.684(1)		2.601(2)
Cu1−O3			1.971(1)	–	1.962(2)
Cu1−O4			2.657(1)		2.685(2)

In the case of reaction of **2 a** with CuCl_2_, formation of the respective complex was neither observed in solution nor in the solid state. Instead, a redox reaction took place, leading to the diamagnetic salt (**2 a**)^2+^(CuCl_2_^−^)_2_ (oxidized guanidine, Cu^I^ anions), as concluded from the crystal data and the NMR spectra in solution. For comparison, the reaction of **1 a** with 2 equiv. of CuCl_2_ gives a dinuclear copper complex [**1 a**(CuCl_2_)_2_] that changes its electronic structure with the environment (Scheme 4).^46^ In the solid state and CH_2_Cl_2_ solution, a paramagnetic dinuclear Cu^II^ complex with reduced, neutral guanidine ligand is present, and in more polar solvents (CH_3_CN or DMSO) a diamagnetic dinuclear Cu^I^ complex with oxidized, dicationic guanidine ligand.

**Figure 8:**
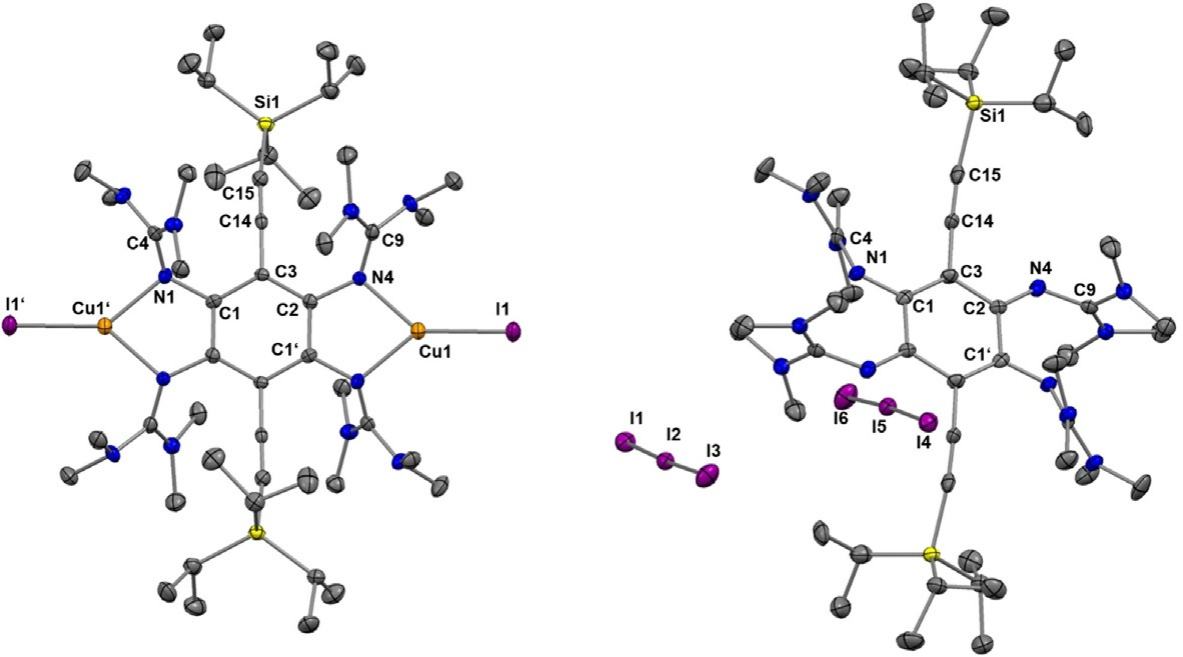
Illustration of the solid‐state structures of the dinuclear Cu^I^ complex [**2 a**(CuI)_2_] (left) and the salt (**2 a**)^2+^(I_3_^−^)_2_ formed upon oxidation of [**2 a**(CuI)_2_] with I_2_. Displacement ellipsoids drawn at the 50 % probability level. All hydrogen atoms omitted.

In another experiment, we reacted **2 a** with CuI. This reaction leads to the dinuclear Cu^I^ complex [**2 a**(CuI)_2_] in 82 % isolated yield. Figure 8 displays the structure of the complex in the solid state. As expected, the imino N=C double bond lengths increase upon coordination, from 1.294(4)/1.284(4) Å in **2 a** to 1.326(3)/1.327(3) Å in [**2 a**(CuI)_2_]. In the UV‐vis spectrum (CH_2_Cl_2_ solution), the band in the visible region experiences a slight bathochromic shift (447 nm for [**2 a**(CuI)_2_] in CH_2_Cl_2_ solution compared with 433 nm for **2 a** in THF solution). Interestingly, the fluorescence is completely extinguished, in line with the results obtained for coordination of Cu^I^ to tetrakisguanidino‐phenazine ligands.^55^ TD‐DFT calculations (B3LYP/def2‐TZVP) found a relatively strong electronic excitation (HOMO‐2→LUMO) at 453.5 nm and a weak excitation (HOMO→LUMO) at 486.4 nm (see Supporting Information, Figures S51 and S52). While the LUMO is centred predominantly at the C_6_ ring and the ethynyl groups, the HOMO and HOMO‐1 are located on the C_6_ ring, the guanidino groups and the CuI groups. Hence the orbitals involved in the electronic excitations are significantly different to those involved for free **2 a**.

**Scheme 5:**
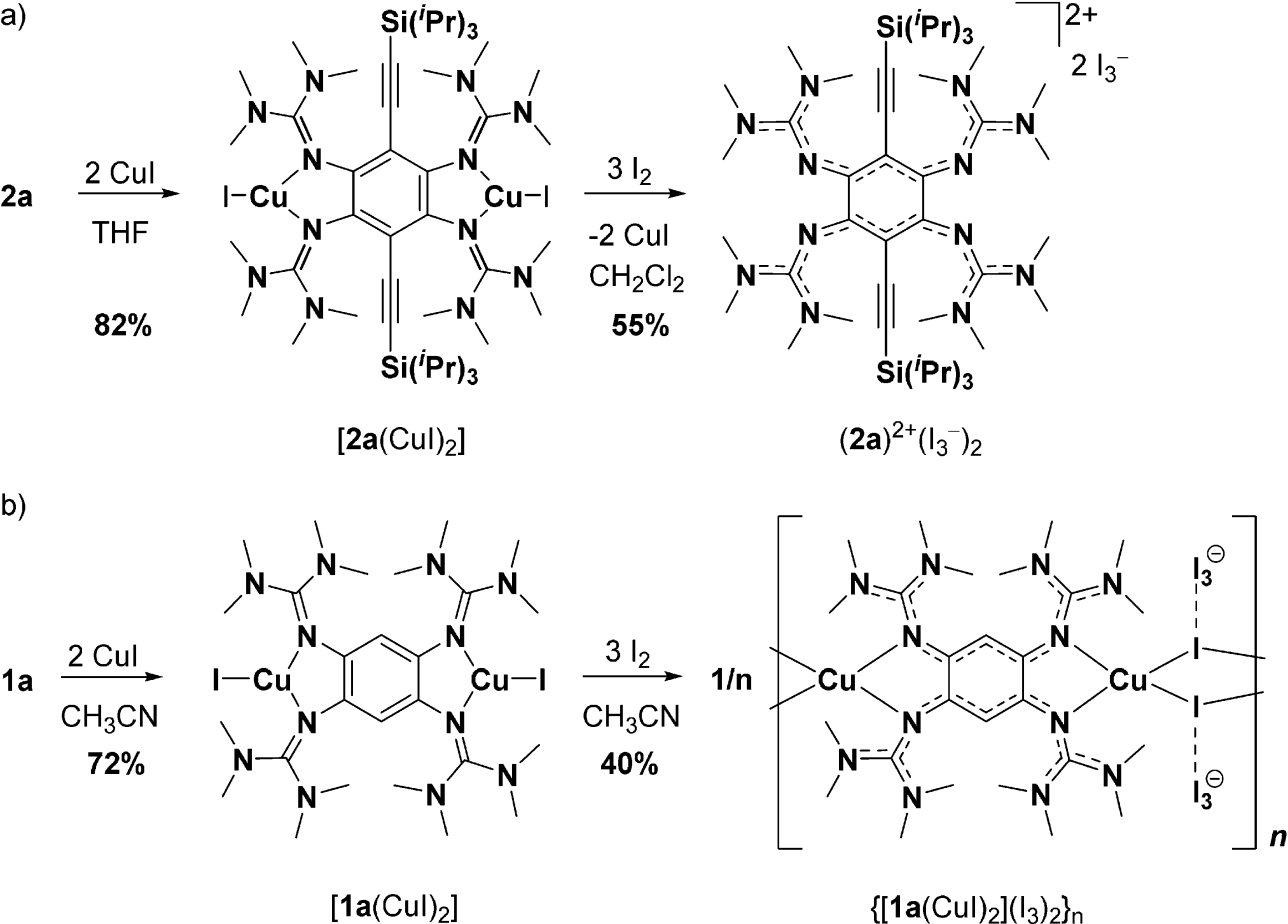
a) Synthesis of a dinuclear Cu^I^ complex of **2 a** and its oxidation with I_2_. b) Illustration of the reaction sequence for the analogue reaction with **1 a**. The different solvents applied in the reactions are due to the large differences in solubility between **1 a** and **2 a** and their complexes.

Then, we reacted the complex [**2 a**(CuI)_2_] with an excess of I_2_ (3 equivalents) in an attempt to isolate a complex with an oxidized guanidine ligand unit. However, the metal‐free salt (**2 a**)^2+^(I_3_^−^)_2_ is isolated in pure form in 55 % yield (see Scheme 5 a). This result indicates that the metal‐ligand bonds break upon ligand oxidation. In the case of the analogue complex [**1 a**(CuI)_2_], reaction with I_2_ gives a diamagnetic coordination polymer {[**1 a**(CuI)_2_](I_3_)_2_}_*n*_ with twofold oxidized bridging guanidine ligand units(see Scheme 5 b).^56^ Interestingly, this chain polymer is found to be an electric semiconductor with a relative small band gap of 1.05 eV (as estimated from an Arrhenius plot of the temperature dependence of the electrical conductivity). Hence all attempts to obtain a dinuclear copper complex with the oxidized, dicationic form **2 a** ^2+^ as ligand, failed. The reason for the distinctly different ligand behaviours of **2 a** and **1 a** is not yet clear, but it might arise from the slightly higher redox potential of **2 a** (see Table 3) and probably also from the differences in solubility and applied solvents that might shift possible equilibria to other sides.

**Scheme 6:**
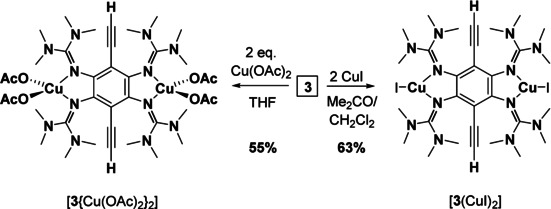
Synthesis of the complexes [**3**(CuI)_2_] and [**3**{Cu(OAc)_2_}_2_].

**Figure 9:**
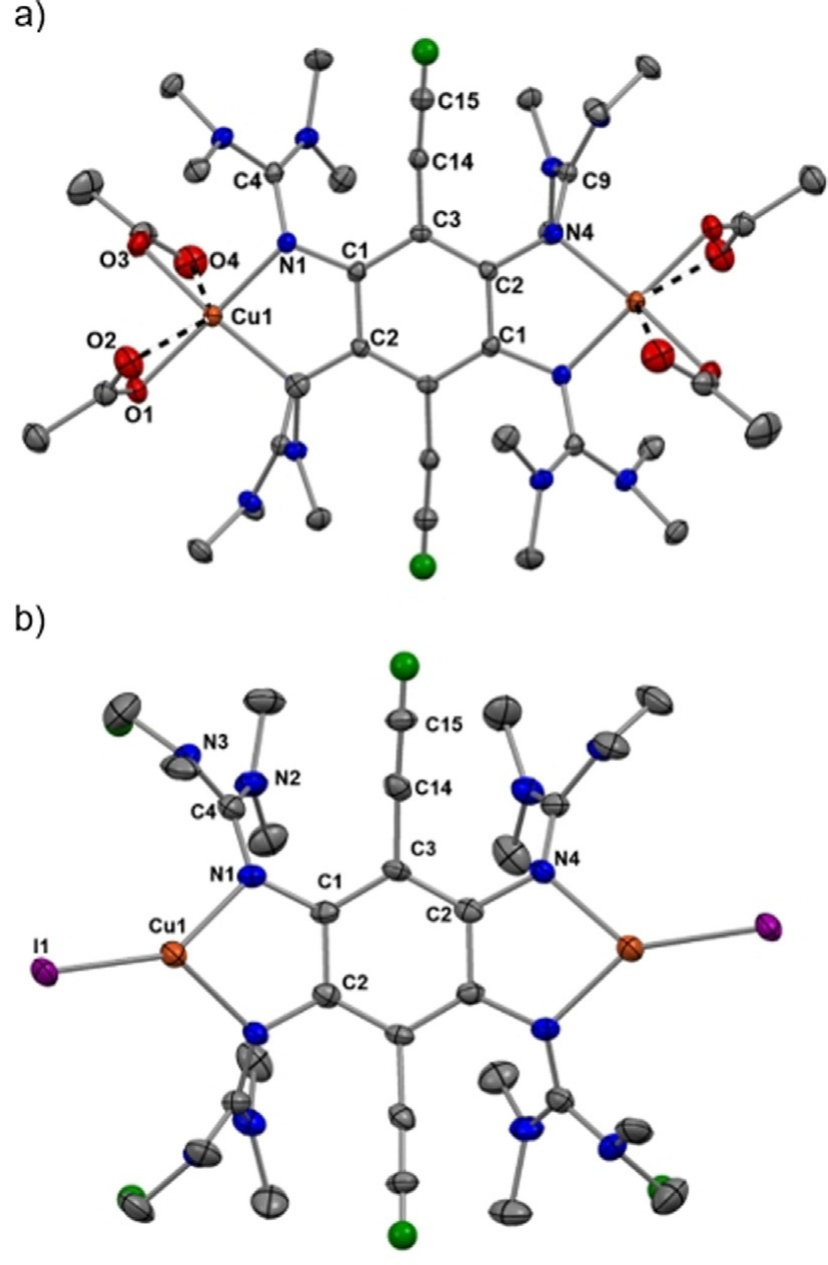
Illustration of the solid‐state structures of a) [**3**{Cu(OAc)_2_}_2_] and b) [**3**(CuI)_2_]. Displacement ellipsoids drawn at the 50 % probability level. Hydrogen atoms bound to carbon were input at calculated positions and refined with a riding model. Ethynyl hydrogens in green, all other hydrogens omitted.

We also studied the coordination chemistry of compound **3** (Scheme 6). Complexation with CuI gave [**3**(CuI)_2_] in 63 % yield. Reaction of **3** with Cu(OAc)_2_ resulted in the formation of the complex [**3**{Cu(OAc)_2_}_2_] in 55 % yield. Hence, dinuclear Cu^I^ as well as Cu^II^ complexes of the neutral ligand could be synthesized. The solid‐state structures of both complexes are illustrated in Figure 9, and selected bond lengths are compiled in Table 4. In both cases, the fluorescence is completely extinguished upon copper coordination (see the analysis of the electronic excitations for [**3**(CuI)_2_] with TD‐DFT in the Supporting Information, Figures S49 and S50).

Cyclic voltammograms of [**3**{Cu(OAc)_2_}_2_] in CH_2_Cl_2_ solution show only irreversible redox processes (see Supporting Information, Figure S33), for example, two oxidation waves at −0.30 V and −0.05 V, as well as a broad shoulder at −0.45 V. A sharp reduction wave is detected at −0.45 V. The irreversibility of the redox events might point again to the cleavage of the metal‐ligand bonds upon ligand oxidation. The complex [**3**(CuI)_2_] is stable in solution under inert‐gas, but is rapidly transformed to other products upon contact to air (see Supporting Information for a preliminary UV‐vis spectroscopic study on this issue, Figure S30). In this case, C−C coupling reactions might take place. The product is not soluble in standard organic solvents, in line with a polymeric structure. The rational synthesis of such coupling products is an attractive goal, which is however clearly outside the scope of this work. The synthesis of [**3**(CuCl_2_)_2_] was attempted but the complex could not be isolated, suggesting a similar reactivity as compound **2 a**. Again we observe a different behaviour to that of **1 a**, for which the dinuclear copper complex [**1 a**(CuCl_2_)_2_] is formed.

The results of this study show that compounds **2 a** and **3** could be used for the synthesis of dinuclear Cu^II^ and Cu^I^ complexes. However, with the oxidized form of the ligands, the complexes are not stable and the metal‐ligand bond is cleaved. This is in marked contrast to the properties of **1 a**, that forms stable complexes in the neutral and in the oxidized form. The differences are most likely caused by the higher redox potentials of **2 a** and **3** compared with **1 a**, and to some extend maybe also by the differences in the applied solvents (which are necessary due to the large differences in solubility) that might affect the position of equilibria. For **3**, further reactivity arises from the terminal alkynyl groups, and is currently studied in our group.

### Reactivity at the terminal alkynyl hydrogens of compound 3

**Scheme 7:**
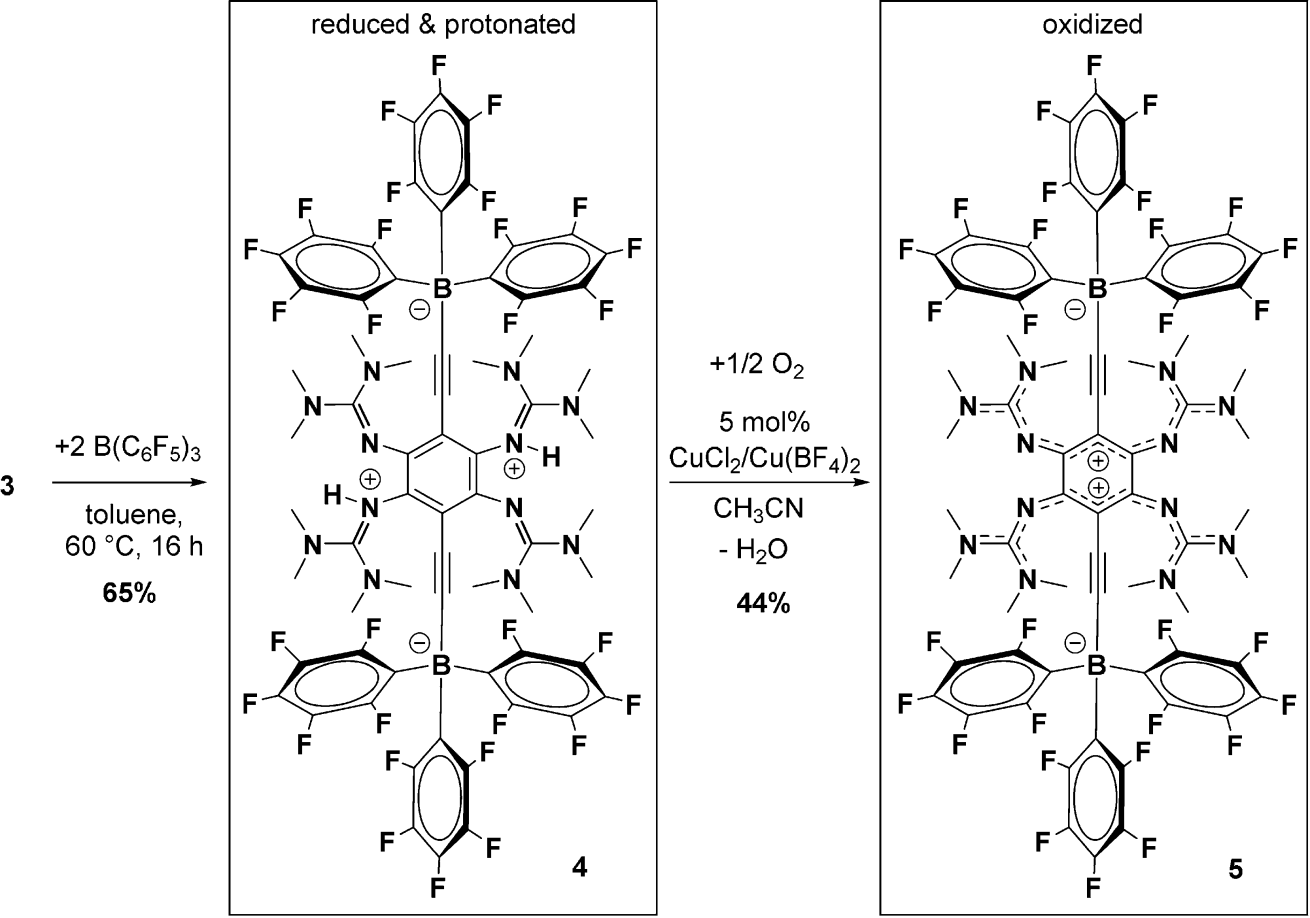
Synthesis of compounds **4** and **5** from **3**.

Next, we tested the possibility to replace the protons from the two terminal alkynyl groups by reaction with a Lewis acid. Indeed, reaction of compound **3** with two equivalents of tris(pentafluorophenyl)borane in toluene at 60 °C gives the neutral zwitterionic bis‐alkynylboronate compound **4** in 65 % isolated yield (Scheme 7). In this reaction, the proton of each terminal alkyne group is replaced by the borane, and the released proton captured by one of the guanidino groups. The reaction is an example of terminal alkyne activation by frustrated Lewis pairs. The previously reported reactions of a terminal alkyne RCCH (various rests R, for example, Ph or H, were tested) with the frustrated Lewis pair combination B(C_6_F_5_)_3_ and a bulky Lewis base LB (e.g. *t*Bu_3_P) yield salts [LBH]^+^[RCCB(C_6_F_5_)_3_]^−^ that could react further with the Lewis base or acid.^57^, ^58^, ^59^ In our reaction, the terminal alkyne and the basic guanidino groups are assembled in one molecule, and therefore an overall neutral compound is obtained.

**Figure 10:**
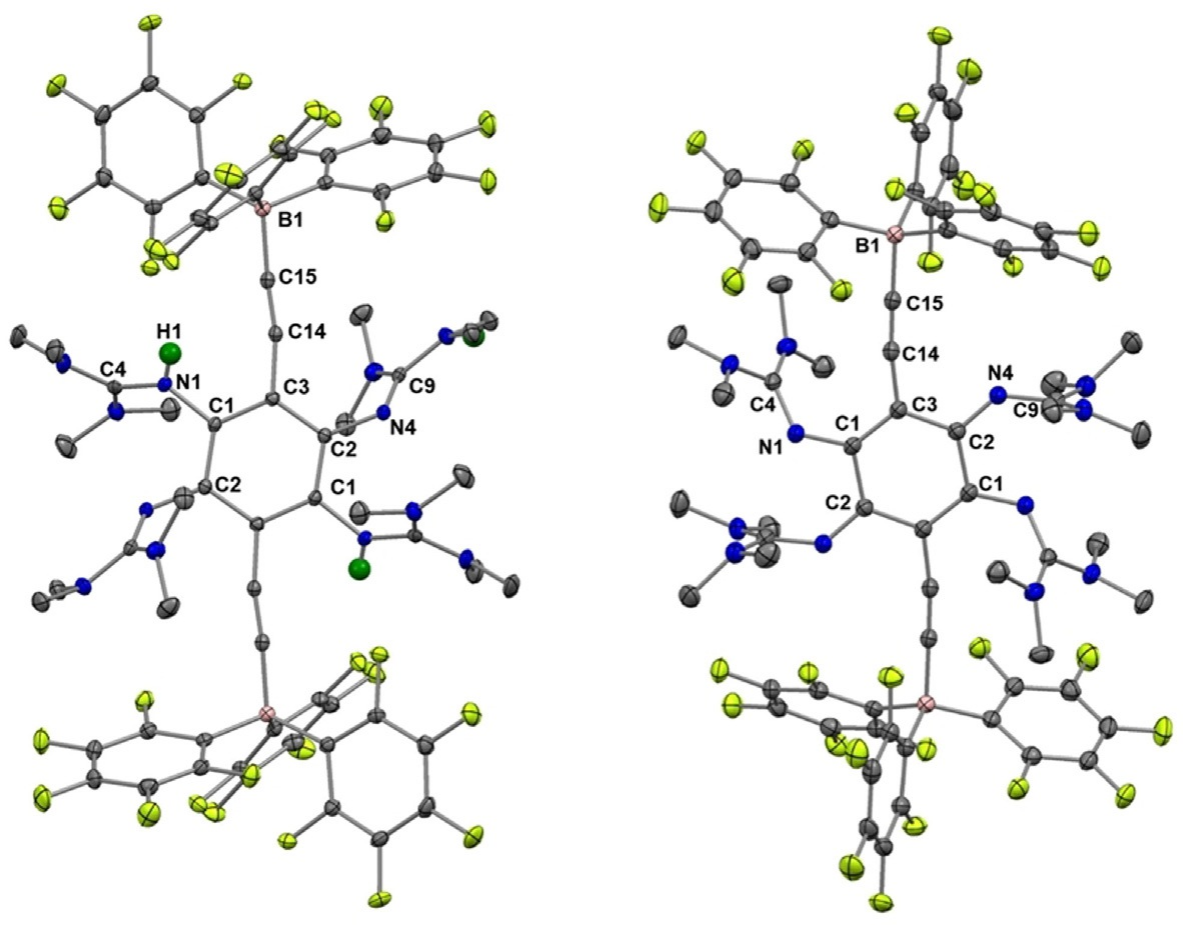
Illustration of the solid‐state structures of **4** and **5** in the solid state. Displacement ellipsoids drawn at the 50 % probability level. Hydrogen atoms bound to nitrogen (green colour) were located in difference Fourier syntheses and refined, either fully or with appropriate distance and/or symmetry. Methyl hydrogen atoms omitted.

The compound is soluble in CH_2_Cl_2_ (in contrast to **2 a**/**2 b** or **3** without signs of decomposition) and acetone, but insoluble in most other solvents (including CH_3_CN). It can be crystallized from a saturated dichloromethane solution. Compound **4** is only weakly fluorescent, with the maximum of emission at 445 nm (*λ*_ex_=315 nm), showing a significant shift compared to 500 nm for compound **3** (*λ*_ex_=420 nm) The fluorescence signal is extremely temperature‐sensitive. It rises at lower temperatures and decreases at higher temperatures (see Supporting Information, Figure S37). Twofold deprotonation of this compound would result in a dianionic, extremely electron‐rich GFA. Unfortunately, all attempts to deprotonate this compound (using triethylamine, butyllithium or sodium amide) failed and resulted in the recovery of unreacted **4**. On the other hand, oxidation of **4** coupled with deprotonation using catalytic amounts of copper salts with O_2_ is successful, giving the zwitterionic compound **5** in 44 % isolated yield. The catalyst is equal to that previously used for oxidation of protonated **1 a** with O_2_ (see also Scheme 1).^30^ The new compound **5** is quite soluble in Me_2_CO or THF, but to our surprise almost insoluble in CH_2_Cl_2_. The solid‐state structures of **4** and **5** are shown in Figure 10, and selected structural parameters are compiled in Table 5. In **4**, the C−C bond distances in the central C_6_ ring vary only slightly (1.402(3)/1.407(3) and 1.415(3) Å for C1‐C2/C1‐C3 and C2‐C3). By contrast, they vary much in **5** (1.497(2)/1.382(3)/1.449(3) Å for C1−C2/C1−C3 and C2−C3), indicating loss of aromaticity. Moreover, the N1−C1 and N4−C2 bond lengths are considerably shorter in **5** compared with **4**. On the other hand, the effect of oxidation on the bond lengths within the alkynyl groups is miniscule. Hence the structural comparison between **4** and **5** is in line with the Lewis structures in Scheme 7.

**Table 5:** Comparison of selected bond lengths (in Å) of compounds **4** and **5**.

Bond	**4**	**5**
N1−C1 N4−C2	1.421(3) 1.399(3)	1.344(3) 1.290(3)
N1−C4 N4−C9	1.347(2) 1.307(3)	1.334(2) 1.349(3)
C1−C2	1.402(3)	1.497(2)
C1−C3	1.407(3)	1.382(3)
C2−C3	1.415(3)	1.449(3)
C3−C14	1.437(2)	1.429(2)
C14−C15	1.208(3)	1.204(3)
C15−B1	1.595(3)	1.592(2)

As observed upon oxidation of **2 a** or **3**, the electronic absorption of **4** in the visible region experiences a bathochromic shift upon oxidation (accompanied in this case by deprotonation), from 401 nm in **4** to 455 nm in **5**, and also a massive increase in its extinction coefficient (by a factor of 3.6). The fluorescence, being already small in **4** at room temperature, is extinguished in **5**.

**Figure 11:**
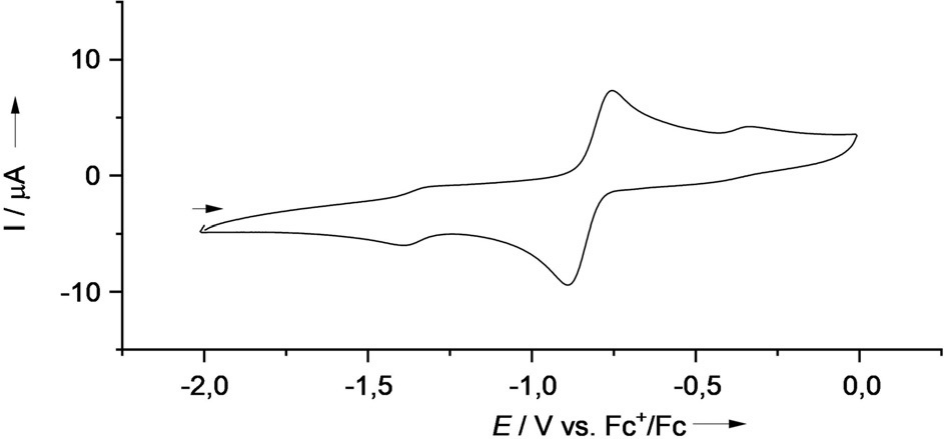
Cyclic voltammogram for **5** in DMF solution (1 mm) (with 0.1 m N(*n*Bu)_4_PF_6_ as supporting electrolyte, Ag/AgCl reference electrode, scan rate 100 mV s^−1^). Potentials given vs. the redox couple ferrocenium/ferrocene (Fc^+^/Fc).

Cyclic voltammetry was used to obtain information about the reduction potential (see Figure 11). In DMF solution, a reversible two‐electron redox process, assigned to the redox couple **5**/**5** ^2−^, is detected at *E*_1/2_=−0.83 V vs. Fc^+^/Fc (*E*_ox_=−0.75 V). A one‐electron process, assigned to the redox couple **5^⋅^** ^+^/**5**, occurs at *E*_1/2_=+0.91 V vs. Fc^+^/Fc (*E*_ox_=+0.97 V). Another oxidation wave at *E*_ox_=+1.26 V vs. Fc^+^/Fc clearly belongs to an irreversible redox event, presumably leading to degradation. Moreover, the voltammogram shows weaker waves (at −0.33 V in direction of oxidation and −1.40 V in direction of reduction). These waves are presumably caused by the extremely high reactivity of **5** ^2−^, that quickly undergoes reactions with dioxygen or other oxidizing impurities. Hence the redox potential of the reduced, dianionic form **5** ^2−^ is significantly lower than those of **1 a** or **2 a**. In fact, **5** ^2−^ has the lowest redox potential of all tetrakisguanidines. On the other hand, its redox potential is still slightly higher than that of the strongest guanidine electron donor, hexakis(*N*,*N*’‐dimethyl‐*N*,*N*’‐ethyleneguanidino)‐benzene, for which an *E*_1/2_ value of −0.96 V vs. Fc^+^/Fc was obtained.^27^ So far, it was not possible to isolate a salt of the dianion **5** ^2−^, which appears to be extremely reactive and sensitive to dioxygen.

## Conclusions

In this work the chemistry of redox‐active 1,2,4,5‐tetrakis(tetramethylguanidino)‐3,6‐diethynyl‐benzenes (compounds **2 a**, **2 b** and **3**) are studied. Substitution of the remaining two hydrogens of the redox‐active guanidine 1,2,4,5‐tetrakis(guanidino)benzene by ethynyl groups leads to redox‐active compounds with redox‐state dependent fluorescence. The fluorescence of the neutral reduced forms is extinguished upon oxidation. The four guanidino groups allow the use of the compounds as redox‐active bridging ligands in several dinuclear Cu^I^ and Cu^II^ complexes. In contrast to 1,2,4,5‐tetrakis(guanidino)benzene, the guanidine‐metal bond is cleaved upon ligand oxidation.

One of the new compounds synthesized in this work has two terminal alkynyl groups (**3**). Reaction of this compound with two equivalents of the Lewis acid B(C_6_F_5_)_3_ leads to migration of the two C−H protons to the guanidino groups and formation of two new C−B bonds by addition of two equivalents of the borane (**4**). Hence the combination **3**/ B(C_6_F_5_)_3_ acts as a frustrated Lewis pair that activates the terminal alkyne groups. The catalytic oxidation/deprotonation of **4** with dioxygen leads to the first redox‐active guanidine that is neutral (instead of dicationic) in its twofold oxidized state (**5**). Consequently, its reduction occurs at the lowest reduction potential ever measured for redox‐active tetrakis‐guanidine compounds.

The results of this study show that redox‐active 1,2,4,5‐tetrakis(tetramethylguanidino)‐3,6‐diethynyl‐benzenes display a diverse chemistry. The topic of ongoing research in our group is their use (after substitution of the protons in **3** by organic groups with suitable functionalities) as building blocks for the construction of metal–organic frameworks. We are also systematically studying how substituents at the alkynyl groups affect the redox‐state dependent fluorescence properties.

## Conflict of interest

The authors declare no conflict of interest.

## Supporting information

As a service to our authors and readers, this journal provides supporting information supplied by the authors. Such materials are peer reviewed and may be re‐organized for online delivery, but are not copy‐edited or typeset. Technical support issues arising from supporting information (other than missing files) should be addressed to the authors.

Supplementary
