# Supplementary material for: 1,2,4,5‐Tetrakis(tetramethylguanidino)‐3,6‐diethynyl‐benzenes: Fluorescent Probes, Redox‐Active Ligands and Strong Organic Electron Donors
Source: Chemistry. 2020 Jul 10;26(45):10336–47. doi: 10.1002/chem.202001557 (PMC7497081; doi:10.1002/chem.202001557)
Supplement: Supplementary file 1 — Supplementary [file CHEM-26-10336-s001.pdf]

Chemistry–A European Journal

Supporting Information

1,2,4,5-Tetrakis(tetramethylguanidino)-3,6-diethynyl-benzenes: Fluorescent Probes, Redox-Active Ligands and Strong Organic Electron Donors

Conrad Wagner, Franka Kreis, Dennis Popp, Olaf Hübner, Elisabeth Kaifer, and Hans-Jörg Himmel*[a]

Content

		page
1	General experimental information	2
2	Synthesis and characterization of all new compounds	4
2.1	1,2,4,5-tetrakis(dimethylethyleneguanidino)-3,6-bis((triisopropylsilyl)ethynyl)-benzene (**2b**)	4
2.2	Desilylation of 1,2,4,5-tetrakis(tetramethylguanidino)-3,6-bis((triisopropylsilyl)ethynyl)-benzene (**2a**) to 1,2,4,5-tetrakis(tetramethylguanidino)-3,6-bis(ethynyl)-benzene (**3**)	9
2.3	Oxidation of 1,2,4,5-tetrakis(tetramethylguanidino)-3,6-bis(ethynyl)-benzene (**3**) with ferrocenium hexafluorophosphate to give $\mathbf{3}^{2+}(PF_6^-)_2$	14
2.4	Reaction of 1,2,4,5-tetrakis(tetramethylguanidino)-3,6-bis((triisopropylsilyl)ethynyl)-benzene (**2a**) with two equivalents of $CuCl_2$ to give $\mathbf{2a}^{2+}(CuCl_2^-)_2$	19
2.5	Complexation of 1,2,4,5-tetrakis(tetramethylguanidino)-3,6-bis((triisopropylsilyl)ethynyl)-benzene (**2a**) with $Cu(OAc)_2$ to give [**2a**$(Cu(OAc)_2)_2$]	20
2.6	Complexation of 1,2,4,5-tetrakis(tetramethylguanidino)-3,6-bis((triisopropylsilyl)ethynyl)-benzene (**2a**) with CuI to give [**2a**$(CuI)_2$]	23
2.7	Oxidation of the complex **2a**$(CuI)_2$ with iodine to give $\mathbf{2a}^{2+}(I_3)_2$	27
2.8	Complexation of 1,2,4,5-tetrakis(tetramethylguanidino)-3,6-bis(ethynyl)-benzene (**3**) with CuI to give the complex [**3**$(CuI)_2$]	29
2.9	Complexation of 1,2,4,5-tetrakis(tetramethylguanidino)-3,6-bis(ethynyl)-benzene (**3**) with $Cu(OAc)_2$ to give [**3**$(Cu(OAc)_2)_2$]	33
2.10	Addition of tris(pentafluorophenyl)borane to 1,2,4,5-tetrakis(tetramethylguanidino)-3,6-bis(ethynyl)-benzene (**3**) to give compound **4**	36
2.11	Oxidation of **4** with O_2 to give compound **5**	42
3	Results of the quantum chemical calculations	48

1) General experimental information

All reactions were carried out under a dry argon atmosphere by using standard Schlenk technique. The solvents acetonitrile, toluene, dichloromethane, diethyl ether and tetrahydrofuran were dried with a MBraun Solvent Purification System and stored over molecular sieves (4 Å) after being degassed by the freeze-pump-thaw method. Other solvents were purchased from Acros Organics and degassed and stored similarly. Infrared spectra were recorded as KBr pellets with a BIORAD *Excalibur FTS 3000* or as solids on an ATR crystal with an AGILENT *Cary* 630 FTIR spectrometer. UV/Vis spectra were measured on a Varian Cary 5000 spectrophotometer. Fluorescence spectra were acquired with a Varian Cary Eclipse fluorescence spectrophotometer. Fluorescence quantum yields were determined using a PTI Quantum Master 40 with Ulbricht sphere. Cyclic voltammetry relied on a Metrohm Autolab PGSTAT 204 potentiostate/galvanostate and a Ag/AgCl reference electrode. The cyclic voltammograms were recorded at room temperature using $N(nBu)_4PF_6$ (electrochemical grade (≥99.0%), Fluka) as supporting electrolyte (c = 0.1 M). BRUKER *Avance DPX 200*, BRUKER *Avance II 400* and BRUKER *Avance III 600* devices were used for NMR spectroscopy. NMR spectra were recorded at 298 K. Elemental analysis were performed at the Microanalytical Laboratory of Heidelberg University using the *vario EL* and *vario MICRO cube* devices from Elementar Analysensysteme GmbH. ESI mass spectrometry relied on a BRUKER *ApexQe hybrid 9.4 T FT-ICR* at the MS laboratory of Heidelberg University.

Suitable crystals for single-crystal structure determination were taken directly from the mother liquor, taken up in perfluorinated polyether oil and fixed on a cryo loop. Full shells of intensity data were collected at low temperature with a Nonius Kappa CCD diffractometer (Mo-K_α radiation, sealed X-ray tube, graphite monochromator, **2b, 3**, **2a**$^{2+}$$(CuCl_2^-)_2$**,** [**2a**$(CuI)_2$], **2a**$^{2+}$$(I_3^-)_2$ and Bruker D8 Venture, dual source (Mo- or Cu-K_α radiation, microfocus X-ray tube, Photon III detector, compounds [**2**$(Cu(OAc)_2)_2$], **3**$^{2+}$$(PF_6^-)_2$, [**3**$\{Cu(OAc)_2\}_2$], **4**, **5** and [**3**$(CuI)_2$]). Data were processed with the standard Nonius and Bruker (SAINT, APEX3) software package.[1] Multiscan absorption correction was applied using the SADABS program.[2] The structures were solved by intrinsic phasing[3] and refined using the SHELXTL software package (Version 2014/6 and 2018/3).[4] Graphical handling of the structural data during solution and refinement

were performed with OLEX2.[5] All non-hydrogen atoms were given anisotropic displacement parameters. Hydrogen atoms bound to carbon were input at calculated positions and refined with a riding model. Hydrogen atoms bound to nitrogen were located in difference Fourier syntheses and refined, either fully or with appropriate distance and/or symmetry. Crystallographic data for the structures reported in this paper have been deposited in the Cambridge Crystallographic Data Centre (CCDC No. 1991506 for **2a**$^{2+}$($CuCl_2^-$)$_2$, 1991507 for **2b**, 1991508 for **3**, 1991509 for [**2a**{Cu(OAc)$_2$}$_2$], 1991510 for [**3**{Cu(OAc)$_2$}$_2$], 1991511 for **5**, 1991512 for [**3**(CuI)$_2$], 1991513 for [**2a**(CuI)$_2$], 1991514 for **2a**$^{2+}$(I_3^-)$_2$, 1991515 for **4**, and 1991528 for **3**$^{2+}$(PF_6^-)$_2$). These data can be obtained free of charge from The Cambridge Crystallographic Data Centre via www.ccdc.cam.ac.uk/data_request/cif.

[1] a) *DENZO-SMN*, Z. Otwinowski & W. Minor, Processing of X-ray Diffraction Data Collected in Oscillation Mode, Methods Enzymol. 1997, 276, Eds C. W. Carter, R. M. Sweet, Academic Press.; b) *SAINT*, Bruker AXS GmbH, Karlsruhe, Germany **2016.**

[2] a) G. M. Sheldrick, SADABS, Bruker AXS GmbH, Karlsruhe, Germany **2004-2014**; b) L. Krause, R. Herbst-Irmer, G. M. Sheldrick, D. Stalke, J*. Appl. Cryst*. **2015**, *48*, 3.

[3] a) G. M. Sheldrick, SHELXT, *Program for Crystal Structure Solution*, University of Göttingen, Germany **2014-2018**; b) G. M. Sheldrick, *Acta Cryst*. **2015**, *A71*, 3.

[4] a) G. M. Sheldrick, *SHELXL-20xx*, University of Göttingen and Bruker AXS GmbH, Karlsruhe,Germany **2012-2018**; b) W. Robinson, G. M. Sheldrick in: N. W. Isaaks, M. R. Taylor (eds.) „*Crystallographic Computing 4*“, Ch. 22, IUCr and Oxford University Press, Oxford, UK, **1988**; c) G. M. Sheldrick, *Acta Cryst*. **2008**, *A64*, 112; (d) G. M. Sheldrick, *Acta Cryst*. **2015**, *C71*, 3.

[5] O. V. Dolomanov, L. J. Bourhis, R. J. Gildea, J. A. K. Howard, H. Puschmann, OLEX2: A complete structure solution, refinement and analysis program, *J. Appl. Cryst.* **2009**, *42*, 339.

2) Synthesis and characterization of all new compounds

2.1) 1,2,4,5-tetrakis(dimethylethyleneguanidino)-3,6-bis((triisopropylsilyl)ethynyl)-benzene (**2b**)

Synthesis

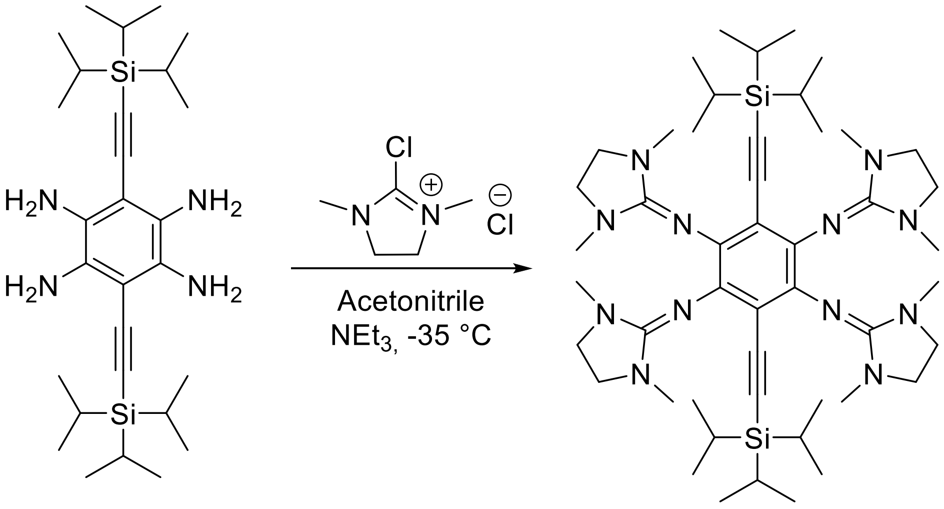

2b

To a solution of 1,3-dimethylimidazolidin-2-one (1.47 ml, 1.55 g, 13.6 mmol, 8 eq.) in chloroform (12 ml) is slowly added oxalyl chloride (5.80 ml, 8.66 g, 68.2 mmol). The reaction mixture is stirred for 16 h under reflux. The solvent is removed under reduced pressure and the residue is washed with diethyl ether. The resulting 2-chloro-1,3-dimethyl-4,5-dihydro-imidazolium chloride is dissolved in acetonitrile (9 ml). Upon cooling, the solution is slowly added to 1,2,4,5-tetra(amino)-3,6-bis-[(triisopropylsilyl)ethynyl]benzene[1] (848 mg, 1.70 mmol, 1 eq.) at –35 °C and the reaction mixture is stirred for 30 min. Then triethylamine (2.0 ml, 14.1 mmol, 15 eq.) is added dropwise, causing an immediate darkening of the suspension. The reaction mixture is stirred for 4 h at a temperature of –35 °C before being slowly warmed to ambient temperature overnight. The solvent is removed under reduced pressure. Then aqueous HCl (10 %, 15 ml) is added, resulting in an almost colourless solution. The solution is filtered and NaOH (25 %, approx. 15 ml) is added to the filtrate until pH 14

is reached. A light yellow solid precipitates, which is extracted under inert gas atmosphere with diethyl ether (approx. 3 × 50 ml) until the organic layer remains colourless. The combined organic layers are dried over K_2CO_3 and the solvent is removed under reduced pressure. The product is purified by recrystallization from acetonitrile. Yield: 203 mg (0.23 mmol, 14 %). Elemental analysis for $C_{48}H_{82}N_{12}Si_2$: calcd. C 65.26, H 9.36, N 19.03, Si 6.36; found C 64.97, H 9.32, N 19.16. ^{1}H NMR (399.89 MHz, THF-*d*8): δ = 3.08 (s, 16 H, CH_2), 2.63 (s, 24 H, CH_3), 1.10 (m, 42 H, $CH(CH_3)_2$) ppm. ^{13}C NMR (100.55 MHz, THF-d_8): δ = 152.0 (NCN_2), 138.7 (C Ar-N), 94.5, 49.4 (CH_2), 34.6 (NCH_3), 19.6 (CH_3), 12.8 (CH) ppm. HR-MS (ESI): $[M+H]^+$ calcd. (m/z) 883.6387, found 883.6404 (100%). UV-vis ($2.3 \cdot 10^{-5}$ M, THF): λ_{max} (ε in $M^{-1}cm^{-1}$) = 314 (23000), 330 (30000), 429 (10000) nm. IR (KBr): ν = 2938 (m, C-H), 2861 (m, C-H), 2140 (w, C≡C), 1654 (s, C=N), 946 (m) cm^{-1}.

Crystal data for $C_{48}H_{82}N_{12}Si_2$: M_r = 883.43, 0.50 × 0.50 × 0.50 mm^3, monoclinic, space group $P2_1/c$, a = 16.669(3), b = 12.601(3), c = 12.601(3) Å, β = 110.29(3)°, V = 2538.9(10) $Å^3$, Z = 2, d_{calc} = 1.156 Mg m^{-3}, Mo K_α radiation (graphite monochromated, λ = 0.71073 Å), T = 120 K, θ_{range} 2.076 to 30.096°, reflections measured: 7433, indep: 4806, R_{int} = 0.1042, final R indices [$I > 2\sigma(I)$]: R_1 = 0.0604, wR_2 = 0.1747.

[1] C. Wagner, O. Hübner, E. Kaifer, H.-J. Himmel, *Chem. Eur. J.* **2019**, *25*, 3781–3785.

Figure S1: ^{1}H NMR spectrum (399.89 MHz, THF-d_8) of **2b**

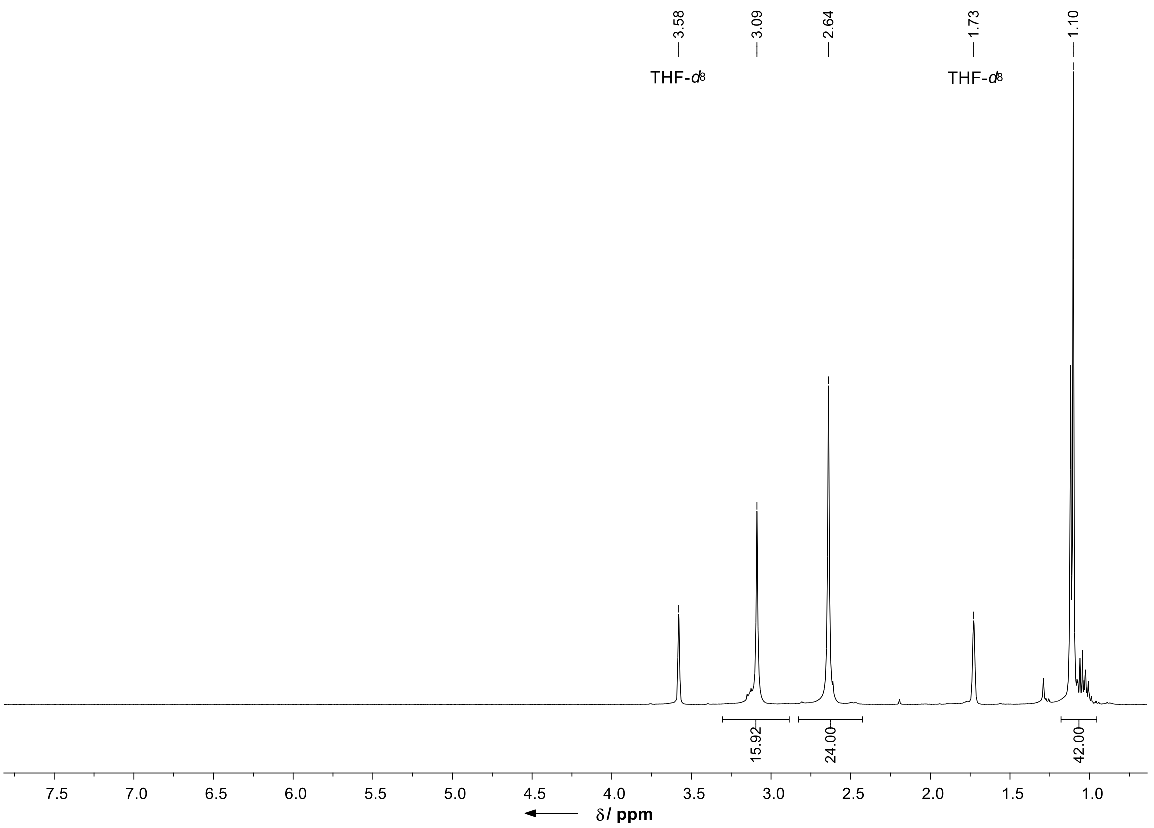

Figure S2: ^{13}C NMR spectrum (100.55 MHz, THF-d_8) of **2b**

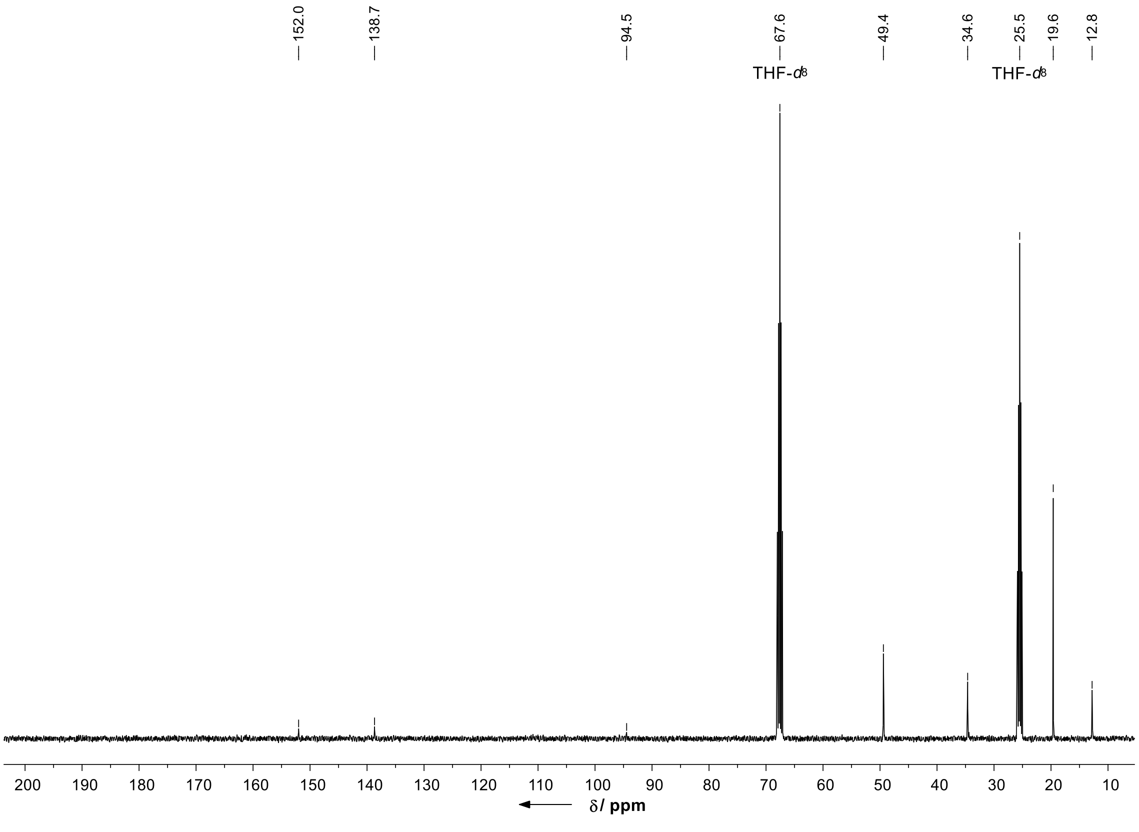

Figure S3: UV-vis spectrum and emission spectrum (2.3·10^{-5} M, THF)

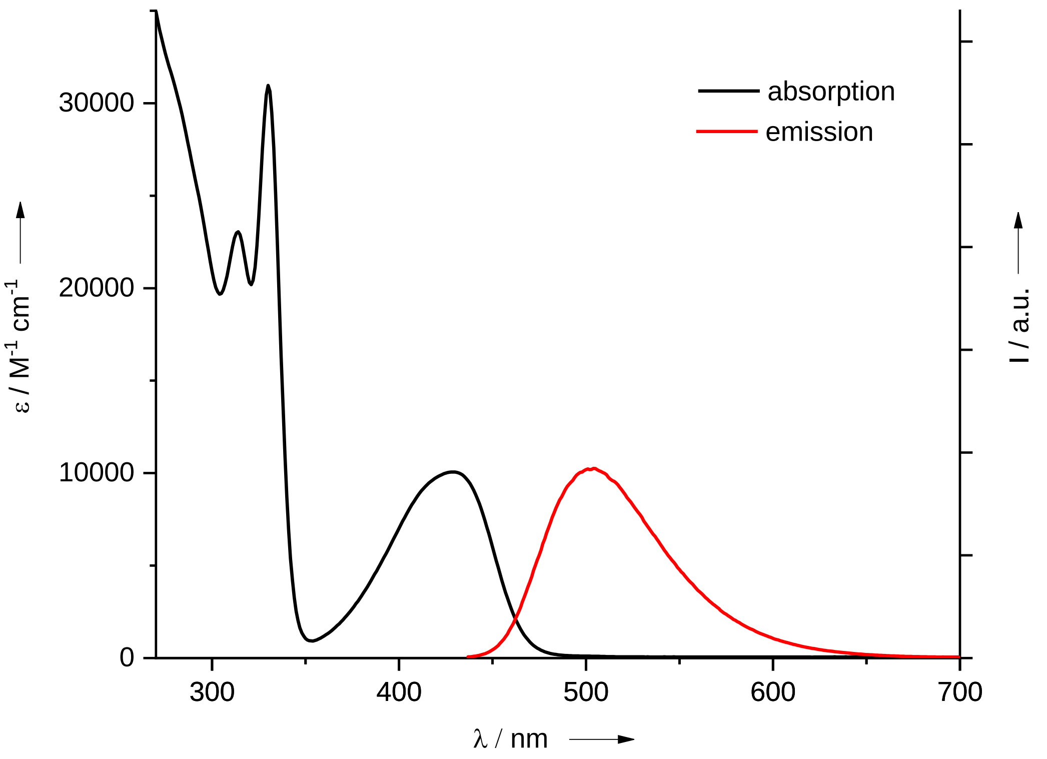

Figure S4: IR spectrum (KBr pellet)

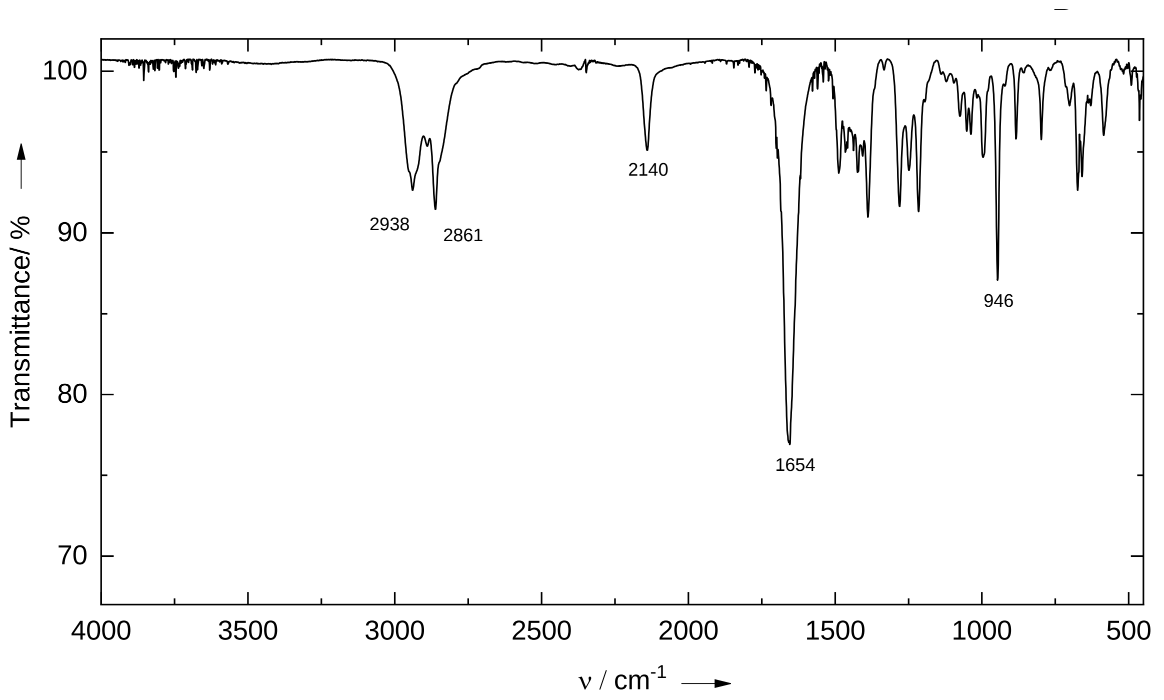

Figure S5: XRD solid state structure

Illustration of the solid-state structure of **2b**. Hydrogen atoms omitted. Displacement ellipsoids are drawn at the 50 % probability level. Color code: C grey, N blue, Si yellow.

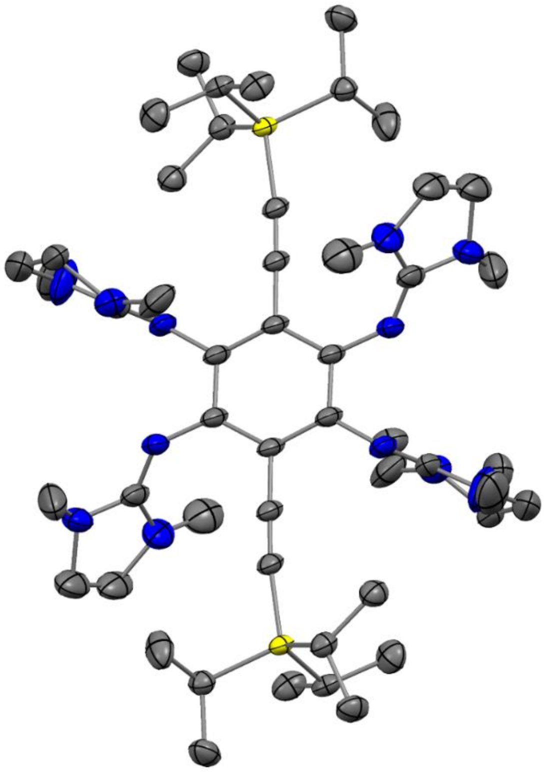

Figure S6: Cyclic voltammogram

Solution of **2b** (1 mM) in CH_2Cl_2, Ag/AgCl reference electrode, with N(*n*Bu)$_4$PF$_6$ (0.1 M) as supporting electrolyte, measured at a scan rate of 100 mV s^{-1}; potentials referenced to the ferrocenium/ferrocene (Fc^+/Fc) redox couple.

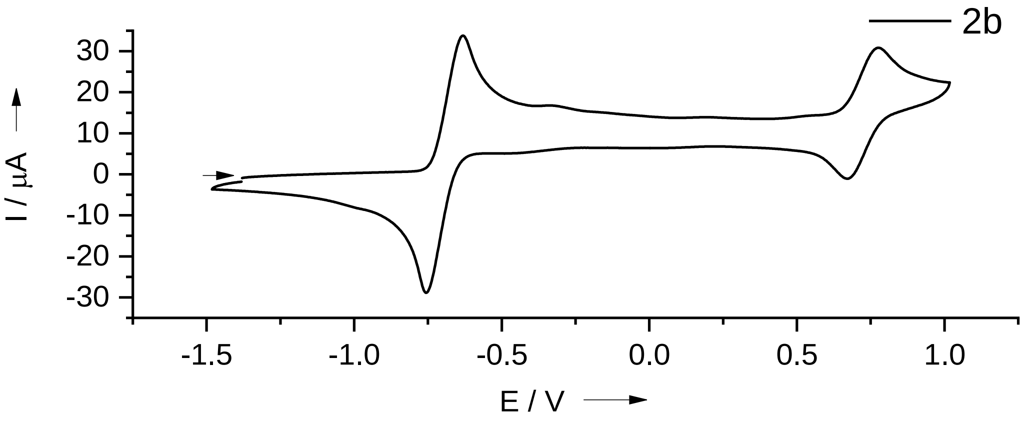

2.2) Desilylation of 1,2,4,5-tetrakis(tetramethylguanidino)-3,6-bis((triisopropylsilyl)ethynyl)-benzene (**2a**) to 1,2,4,5-tetrakis(tetramethylguanidino)-3,6-bis(ethynyl)-benzene (**3**)

Synthesis

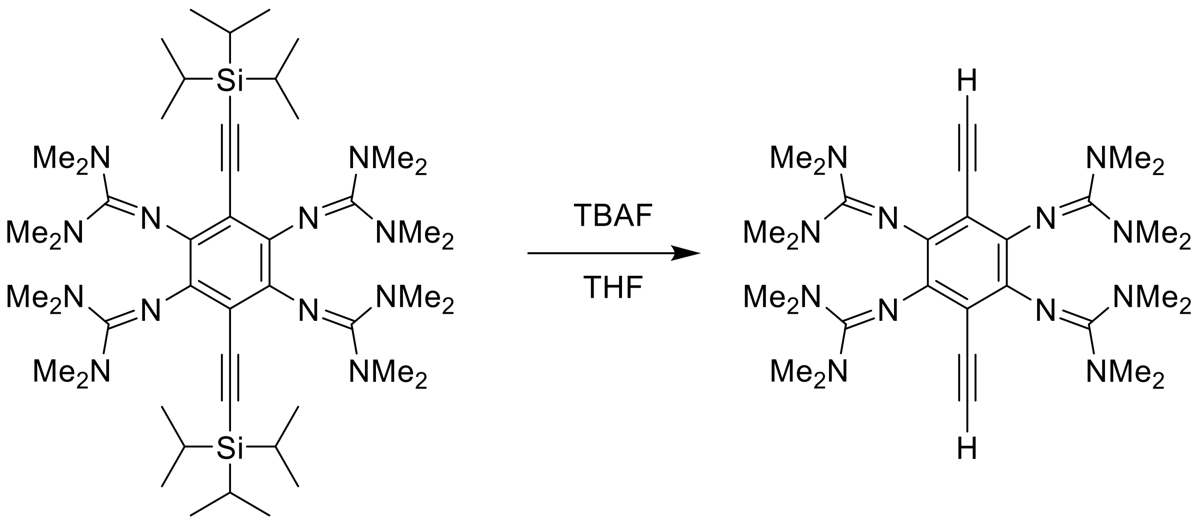

2a **3**

Tetrabutylammonium fluoride (1.0 ml, 1.0 mmol, 1 M in THF) is added to a solution of 400 mg (0.448 mmol) 1,2,4,5-tetrakis(tetramethylguanidino)-3,6-bis((triisopropyl-silyl)ethynyl)-benzene (**2a**) in THF (15 ml). The reaction mixture is stirred at 50 °C for 16 h resulting in a yellow suspension. The solution is filtered off and the precipitate is dried under reduced pressure. Recrystallization from toluene (aprox. 18 ml) yields 1,2,4,5-tetrakis(tetramethylguanidino)-3,6-bis(ethynyl)-benzene (**3**) as yellow crystals suitable for XRD (203 mg, 0.35 mmol, 78 %). Elemental analysis for $C_{30}H_{50}N_{12}$: calcd. C 62.25, H 8.71, N 29.04; found C 62.33, H 8.59, N 29.12. ^{1}H NMR (399.89 MHz, CD_2Cl_2): δ = 3.05 (s, 2 H, C≡CH), 2.74 (s, 48 H, CH_3) ppm. ^{1}H NMR (199.87 MHz, toluene-d_8): δ = 2.74 (s, 2 H, C≡CH), 2.71 (s, 48 H, CH_3) ppm ^{13}C NMR (100.55 MHz, CD_2Cl_2): δ = 39.2 (CH_3) ppm. Due to the low solubility, the intensity of the signals in the ^{13}C NMR spectrum is too low for further signal detection. HR-MS (ESI): $[M+H]^+$ calcd. (m/z) 579.4354, found 579.4357 (100%). UV-vis ($2.3 \cdot 10^{-5}$ M, toluene): λ_{max} (ε in

$M^{-1}cm^{-1}$) = 313 (12500), 420 (6200) nm. IR (KBr): ν = 3260 (w, C≡C-H), 2928 (w, C-H), 2084 (w, C≡C), 1592 (s, C=N), 1374 (m), 1139 (m) cm^{-1}.

Crystal data for $C_{30}H_{50}N_{12}$: M_r = 578.82, 0.40 × 0.40 × 0.25 mm^3, monoclinic, space group $P2_1/n$, a = 11.042(2), b = 13.544(3), c = 21.997(4) Å, β = 96.36(3)°, V = 3269.5(11) $Å^3$, Z = 4, d_{calc} = 1.176 Mg m^{-3}, Mo K_α radiation (graphite monochromated, λ = 0.71073 Å), T = 120 K, θ_{range} 1.769 to 30.085°, reflections measured: 64616, indep: 9576, R_{int} = 0.0758, final R indices [I >2σ(I)]: R_1 = 0.0575, wR_2 = 0.1551.

Figure S7: ^{1}H NMR spectrum (199.87 MHz, toluene-d_8) of **3**

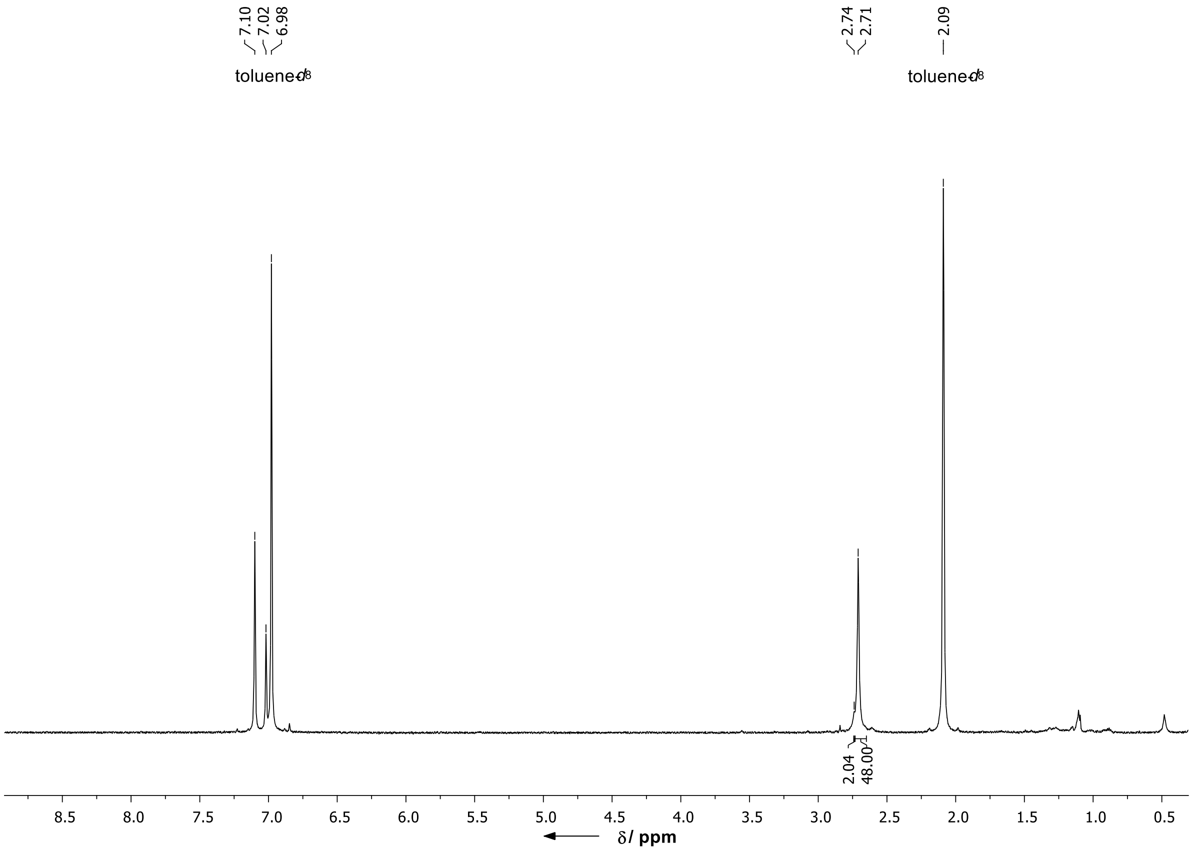

<u>**Figure S8**: UV-vis spectrum and emission spectrum ($2.3\cdot10^{-5}$ M, toluene) of **3**</u>

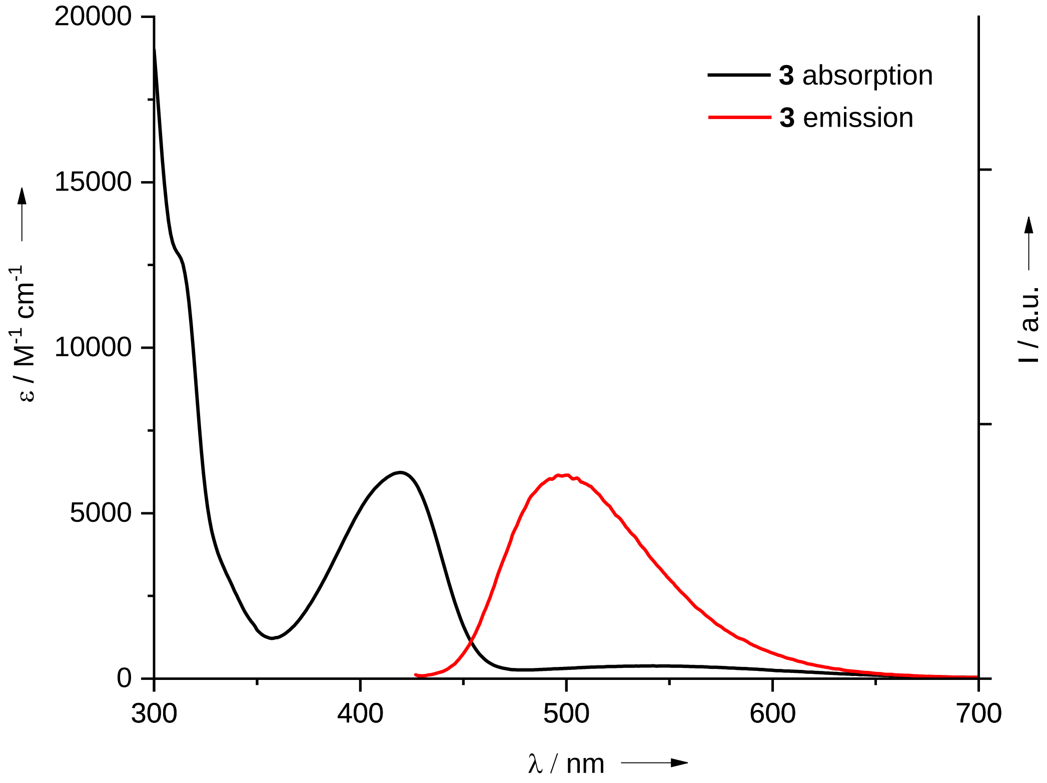

<u>**Figure S9**: IR spectrum (KBr pellet) of **3**</u>

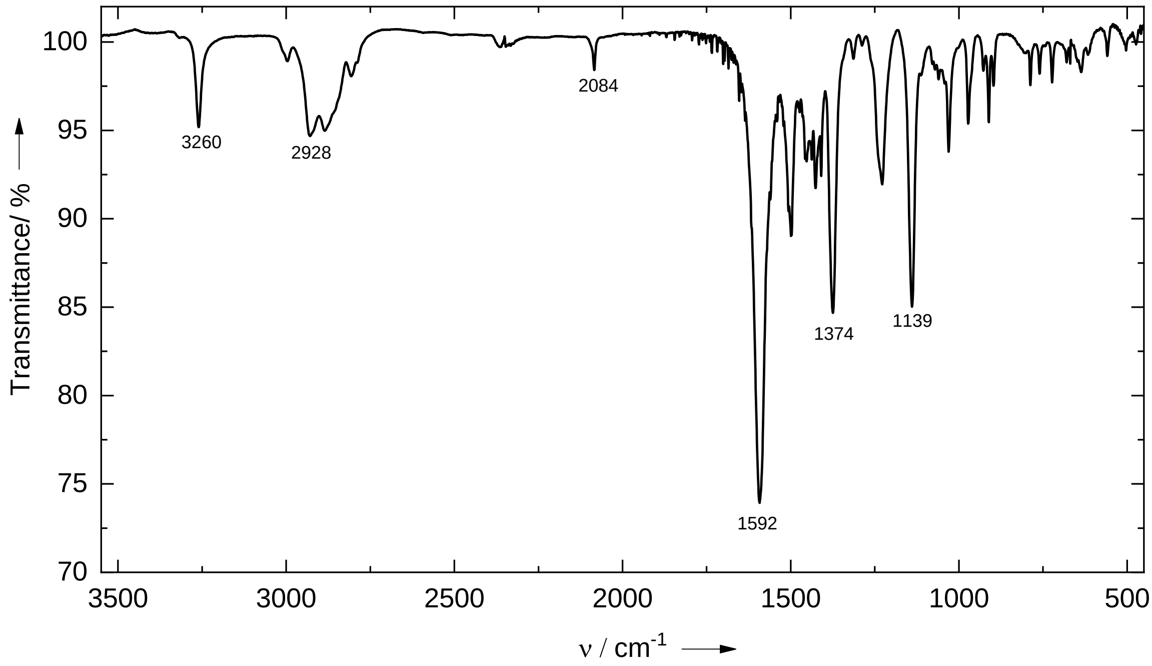

Figure S10: XRD solid state structure of **3**

Illustration of the solid state structure of **3** from two perspectives. Hydrogen atoms (except for terminal alkyne H atoms) are omitted in both representations. Displacement ellipsoids are drawn at the 50 % probability level. Color code: C grey, N blue, H green.

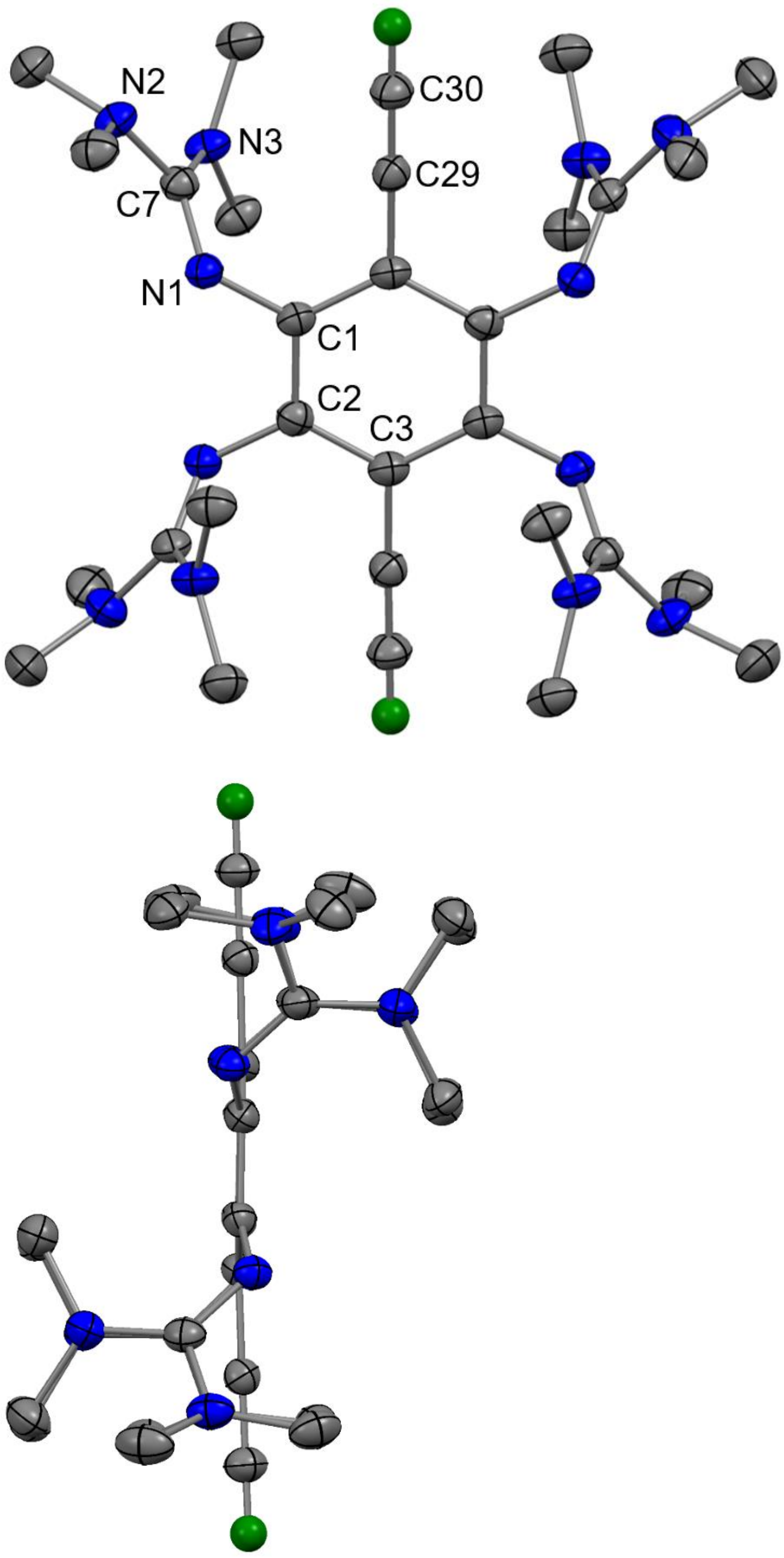

Figure S11: Cyclic voltammograms of **3**

Solution of **3** (1 mM) in CH_2Cl_2, Ag/AgCl reference electrode, with N(*n*Bu)$_4$PF$_6$ (0.1 M) as supporting electrolyte, measured at a scan rate of 100 mV s^{-1}; potentials referenced to the ferrocenium/ferrocene (Fc^+/Fc) redox couple.

a) Voltammogram in the potential window from −2.3 V to +0.3 V.

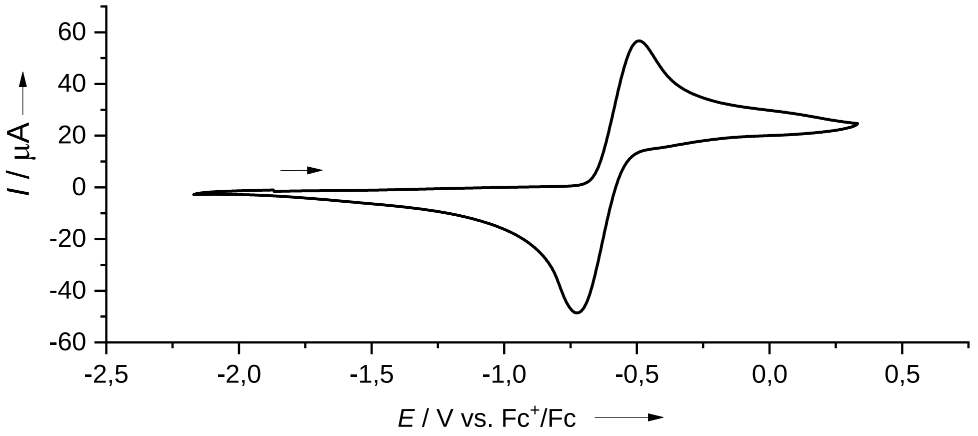

b) Voltammogram in the potential window from −2.3 V to +0.82 V.

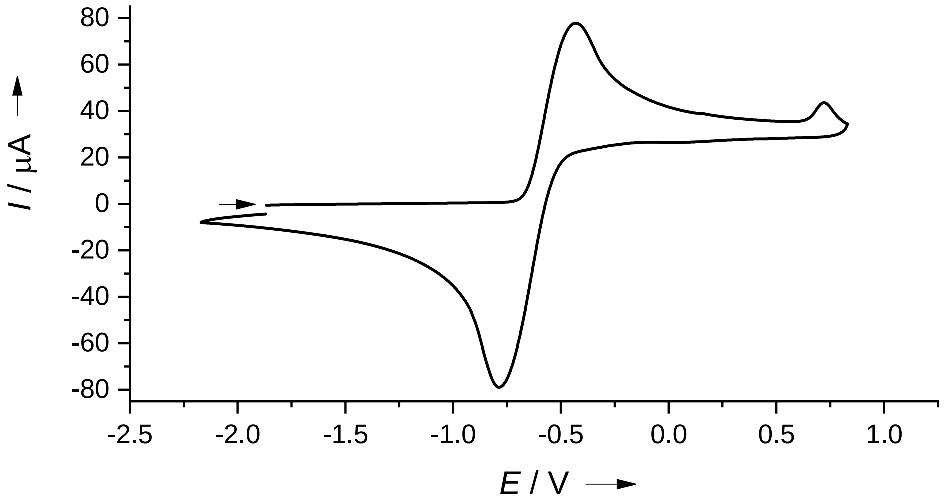

2.3) Oxidation of 1,2,4,5-tetrakis(tetramethylguanidino)-3,6-bis(ethynyl)-benzene (**3**) with ferrocenium hexafluorophosphate to give **3**$^{2+}$(PF_6^-)$_2$

Synthesis

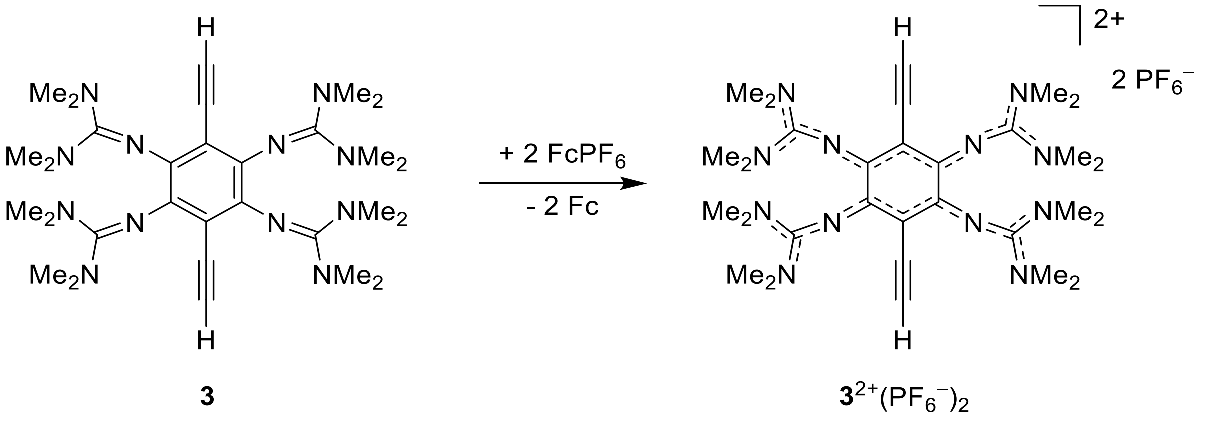

The starting reagents, 20 mg (0.035 mmol, 1.00 eq.) of 1,2,4,5-tetrakis(tetramethylguanidino)-3,6-bis(ethynyl)-benzene (**3**) and 23 mg (0.069 mmol, 1.97 eq.) of ferrocenium hexafluorophosphate, are dissolved in acetonitrile (3 ml). The reaction mixture is stirred at room temperature for 16 h resulting in a deep green solution. After filtration, the solvent is removed under reduced pressure and the residue is washed with diethyl ether (4 × 3 ml). The green solid is dried under reduced pressure (27 mg, 0.031 mmol, 89 %). Crystals suitable for XRD were obtained by slow diffusion of diethyl ether into an acetonitrile solution. Elemental analysis for $C_{30}H_{50}F_{12}N_{12}P_2$: calcd. C 41.48, H 5.80, N 19.35, P 7.13, F 26.24; found C 42.22, H 6.19, N 19.61. ^{1}H NMR (399.89 MHz, CD_3CN): δ = 3.60 (s, 2 H, C≡CH), 2.88 (s, 48 H, CH_3) ppm. ^{13}C NMR (100.55 MHz, CD_3CN): δ = 167.7 (NCN_2), 157.1 (C Ar-N), 95.5 (C Ar-C≡C), 86.2 (C≡C), 78.3(C≡C), 40.7 (CH_3) ppm. ^{19}F NMR (376.27 MHz, CD_3CN): δ = –72.9 (d, *J* = 706 Hz) ppm. ^{31}P NMR (161.88 MHz, CD_3CN): δ = –144.6 (sept, *J* = 706 Hz) ppm. HSQC NMR (CD_3CN): δ = 3.60/86.2, 2.88/40.7 ppm. HMBC NMR (CD_3CN): δ = 3.60/157.1, 3.60/95.5, 2.88/167.7, 2.88/157.1, 2.88/40.7 ppm. HR-MS (ESI): [M-PF_6]$^+$ calcd. (m/z) 723.3918, found 723.3927 (100%). UV-vis ($2.0\cdot10^{-5}$ M, acetonitrile): λ_{max}

(ε in $M^{-1}cm^{-1}$) = 219 (39200), 274 (17700), 330 (11600), 433 (32300) nm. IR (KBr): ν = 3284 (w, C≡C-H), 2959 (w, C-H), 1508 (m), 841 (s, νPF_6^-), 558 (s, δPF_6^-) cm^{-1}.

Crystal data for $C_{30}H_{50}F_{12}N_{12}P_2$: M_r = 868.76, 0.37 × 0.16 × 0.03 mm^3, triclinic, space group $P\bar{1}$, a = 8.1867(3), b = 9.6449(4), c = 12.7910(5) Å, α = 98.638(2), β = 93.595(2), γ = 93.039(2)°, V = 994.56(7) $Å^3$, Z = 1, d_{calc} = 1.450 Mg m^{-3}, Mo K_α radiation (graphite monochromated, λ = 0.71073 Å), T = 120 K, θ_{range} 2.140 to 35.070°, reflections measured: 56442, indep: 8752, R_{int} = 0.0457, final R indices [$I > 2\sigma(I)$]: R_1 = 0.0394, wR_2 = 0.1158.

Figure S12: ^{1}H NMR spectrum (399.89 MHz, CD_3CN) of $\mathbf{3}^{2+}(PF_6^-)_2$

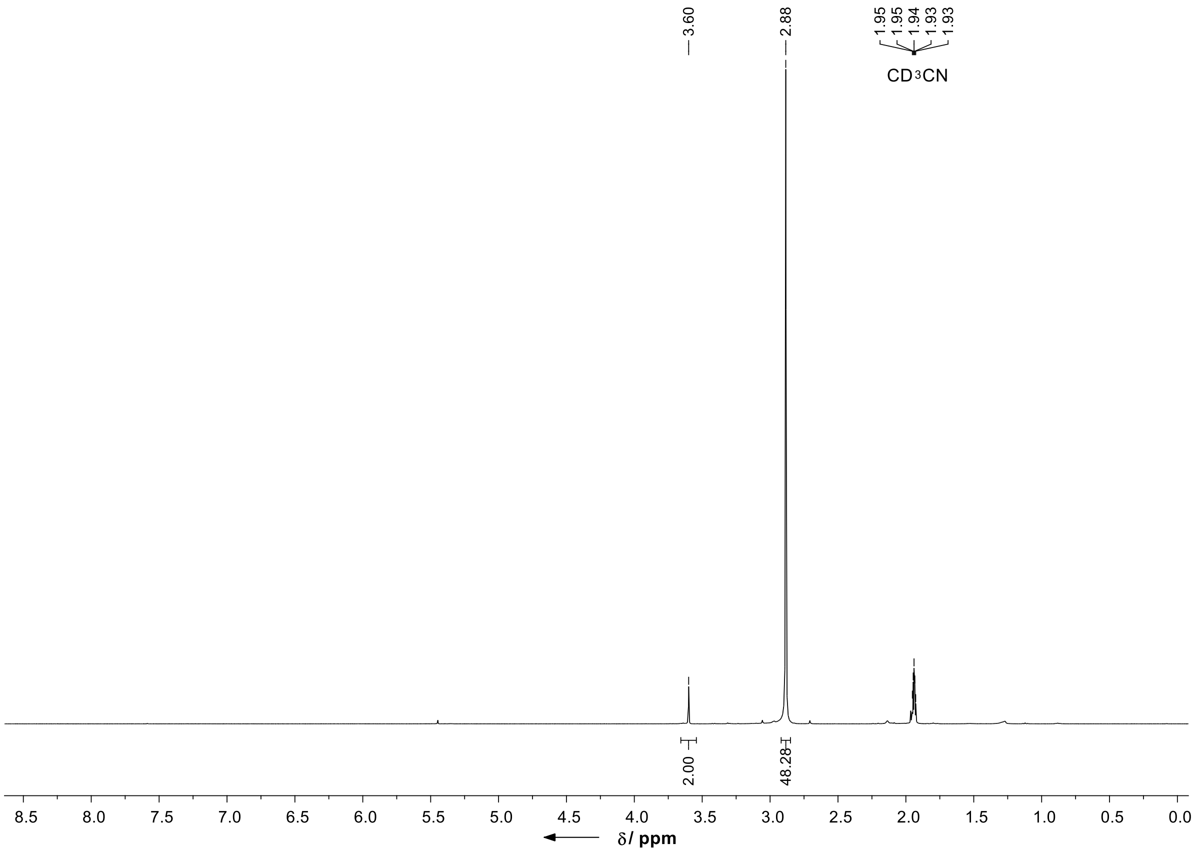

Figure S13: ^{13}C NMR spectrum (100.55 MHz, CD_3CN) of $\mathbf{3}^{2+}(PF_6^-)_2$

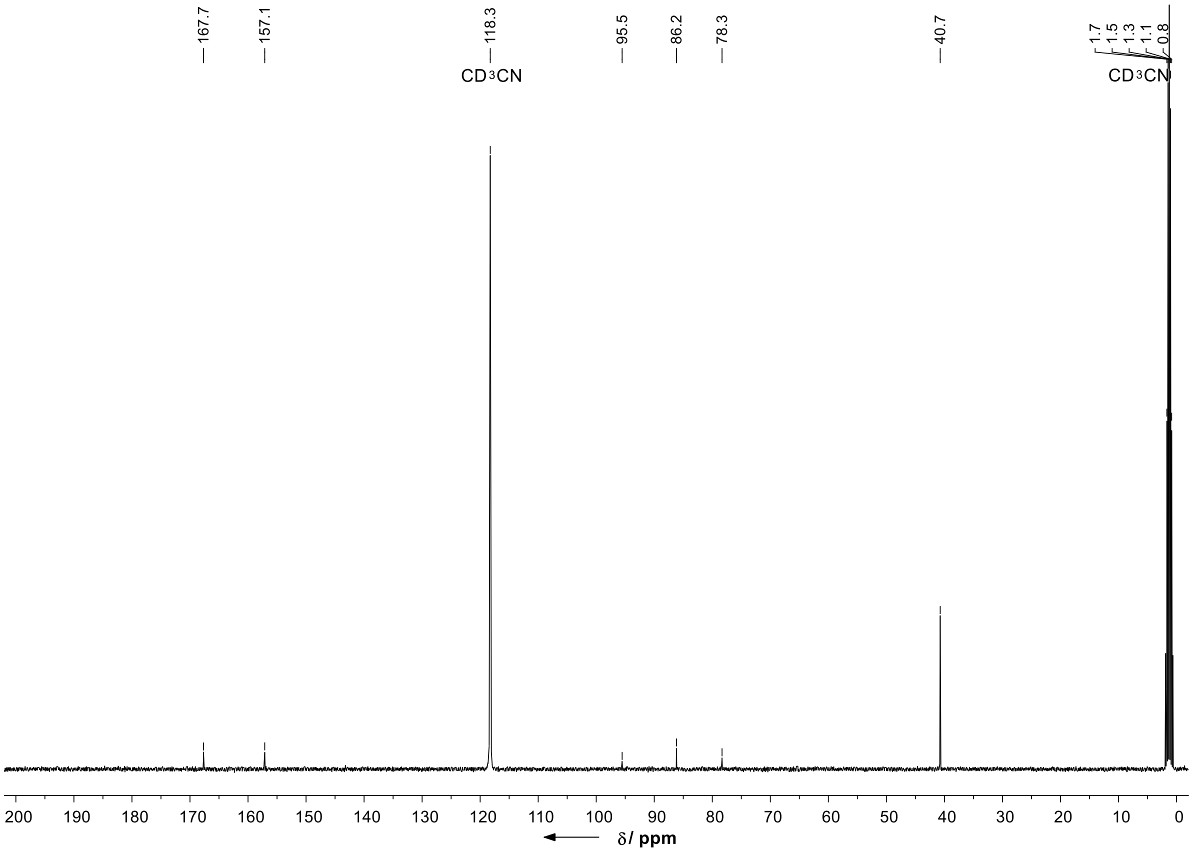

Figure S14: UV-vis spectrum (CH_3CN) of **3**$^{2+}$(PF_6^-)$_2$

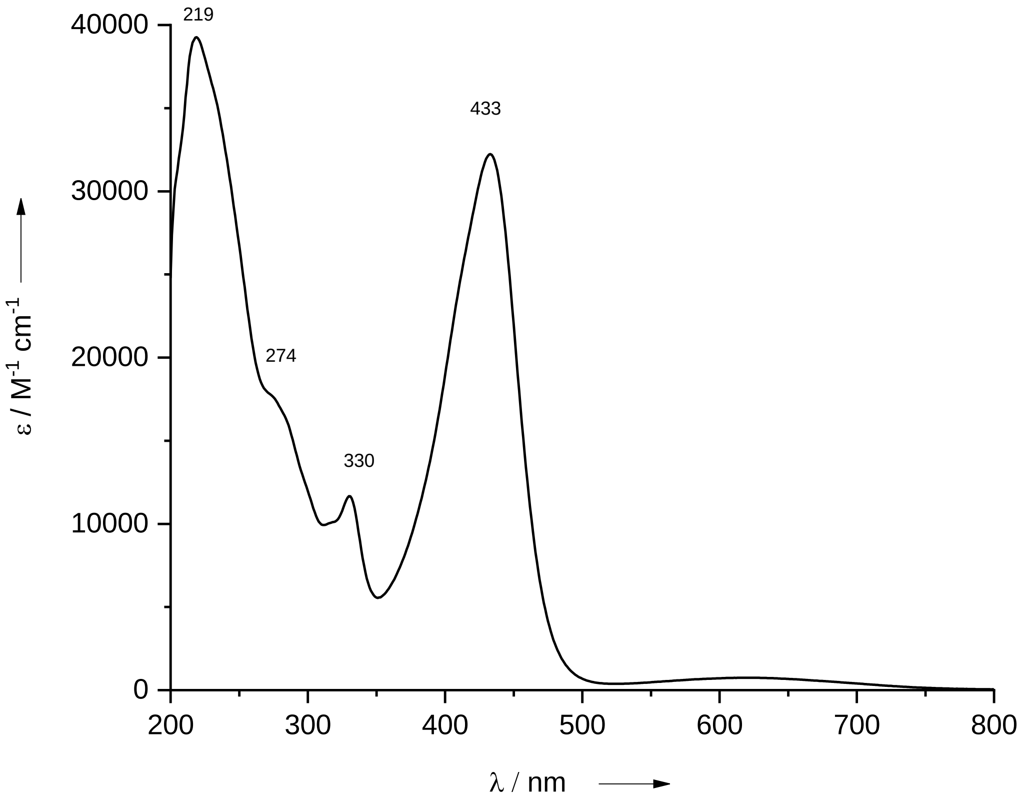

A weak, very broad band is visible at ca. 610 nm. This band might be due to the HOMO → LUMO transition (calculated at 675.9 nm with TD-DFT), which is symmetry-forbidden according to the calculations (since HOMO and LUMO both exhibit a_g symmetry).

Figure S15: IR spectrum (KBr pellet) of **3**$^{2+}$(PF_6^{-})$_2$

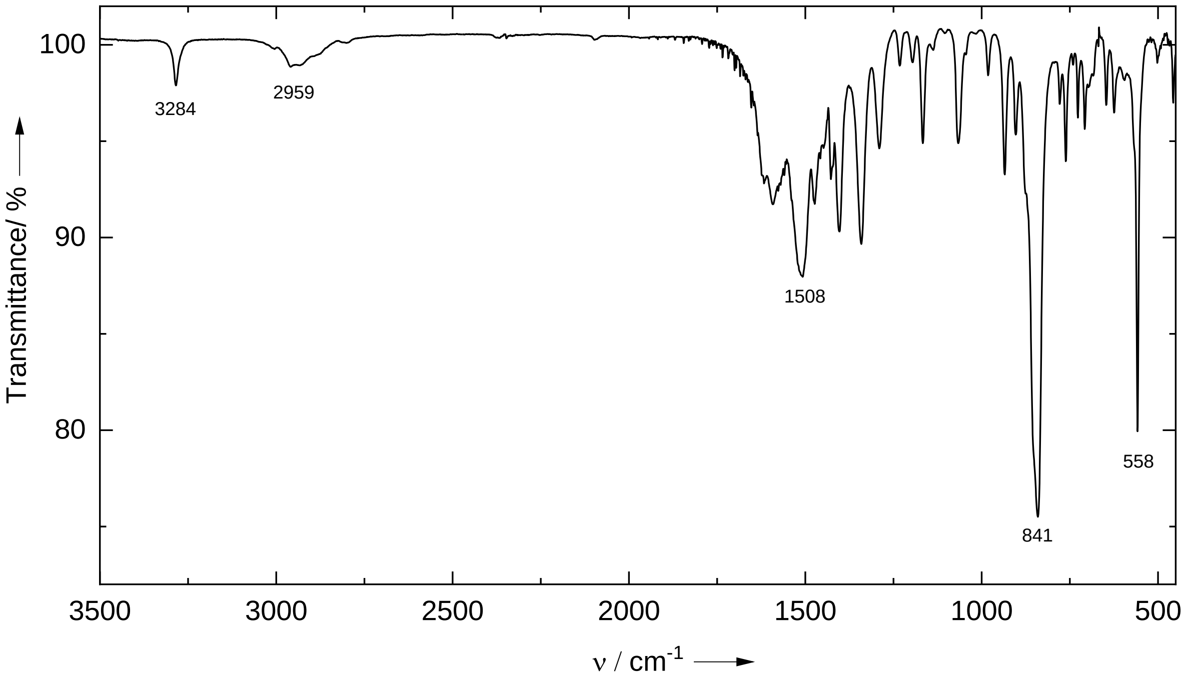

Figure S16: XRD solid state structure of **3**$^{2+}$(PF_6^{-})$_2$

Illustration of the solid state structure of **3**$^{2+}$(PF_6^{-})$_2$. Hydrogen atoms are omitted. Displacement ellipsoids are drawn at the 50 % probability level. Color code: C grey, N blue, P orange, F yellow.

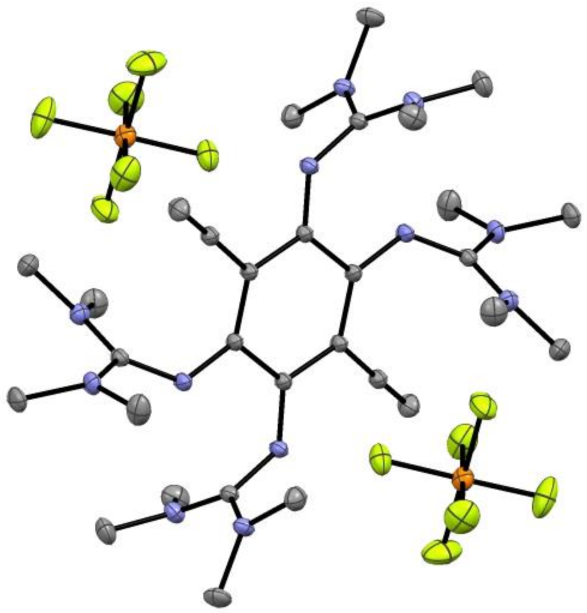

2.4) Reaction of 1,2,4,5-tetrakis(tetramethylguanidino)-3,6-bis((triisopropylsilyl)ethynyl)-benzene (**2a**) with two equivalents of $CuCl_2$ to give **2a**$^{2+}$($CuCl_2^-$)$_2$

Synthesis

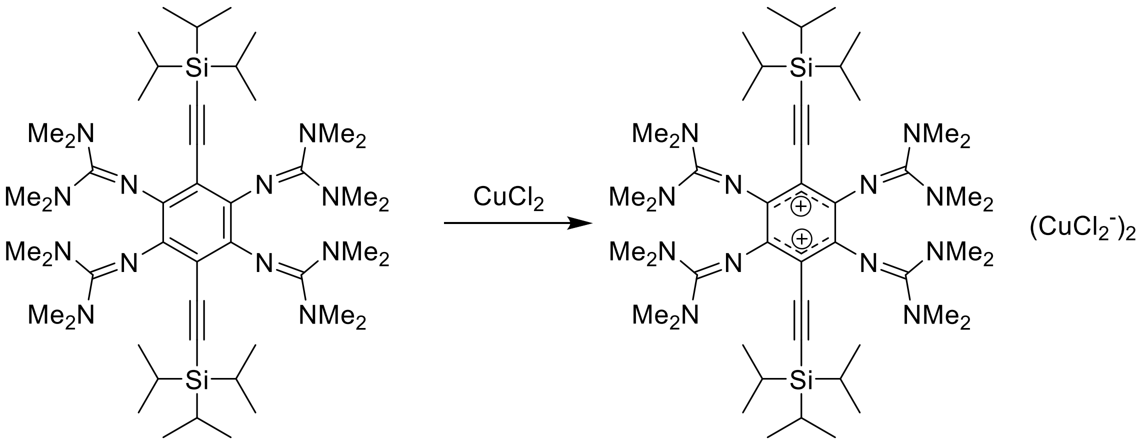

2a **2a^{2+}($CuCl_2^-$)$_2$**

To a solution of $CuCl_2$ (14.3 mg, 0.107 mmol, 1.9 eq.) in acetonitrile (10 ml) **2** (50 mg, 0.056 mmol, 1.0 eq.) is added. The reaction mixture turns green immediately. After stirring at room temperature for 6 h the deep green solution is overlaid with diethyl ether (40 ml) and kept at –20 °C for 6 days resulting in the formation of green needles, suitable for XRD. The solvent is filtered off and the green needles washed with diethyl ether (2 × 10 ml). Elemental analysis for $C_{48}H_{90}N_{12}Cu_2Cl_4Si_2$: calcd. C 49.68, H 7.82, N 14.49, Cu 10.95, Cl 12.22, Si 4.84; found C 47.55, H 7.32, N 14.23.

Crystal data for [$C_{48}H_{90}N_{12}Si_2$] [$CuCl_2$]$_2$: M_r = 1160.37, 0.25 × 0.20 × 0.15 mm^3, triclinic, space group $P\bar{1}$, a = 10.077(2), b = 12.699(3), c = 13.607(3) Å, α = 115.31(3), β = 107.04(3), γ = 92.61(3)°, V = 1475.3(6) Å^3, Z = 1, d_{calc} = 1.306 Mg m^{-3}, Mo K$_\alpha$ radiation (graphite monochromated, λ = 0.71073 Å), T = 120 K, θ_{range} 1.765 to 30.078°, reflections measured: 25134, indep: 8616, R_{int} = 0.0983, final R indices [I >2σ(I)]: R_1 = 0.0452, wR_2 = 0.1095.

2.5) Complexation of 1,2,4,5-tetrakis(tetramethylguanidino)-3,6-bis((triisopropylsilyl)ethynyl)-benzene (**2a**) with $Cu(OAc)_2$ to give [**2a**$(Cu(OAc)_2)_2$]

Synthesis

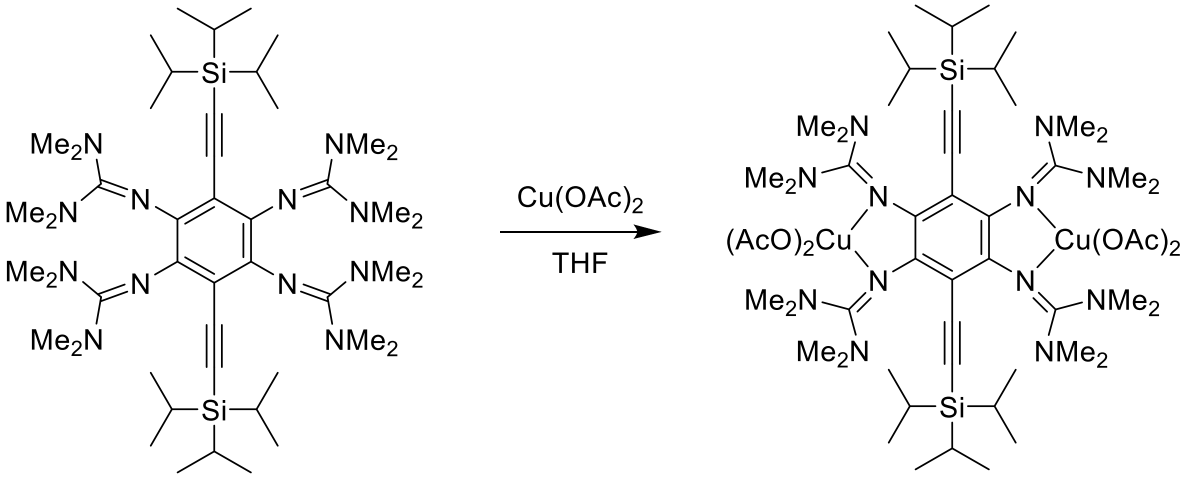

THF (5 ml) is added to **2** (40 mg, 0.045 mmol, 1 eq.) and $Cu(OAc)_2$ (16 mg, 0.090 mmol, 2 eq.). The solution turns green upon addition and is stirred at room temperature for 12 h. The solvent is removed under reduced pressure and the residue extracted with dichloromethane (3 x 3 ml). After filtration, the solvent is removed under reduced pressure before washing the product with pentane (3 x 2 ml). Crystals suitable for XRD were obtained from a saturated acetonitrile solution. Yield 42 mg (25.4 mmol, 55 %). Elemental analysis for $C_{56}H_{102}N_{12}Cu_2O_8Si_2$: calcd. C 53.60, H 8.19, N 13.40, Cu 10.13, O 10.20, Si 4.48; found C 53.61, H 8.69, N 13.31. HR-MS (ESI): [M-$Cu(OAc)_3]^+$ calcd. (m/z) 1012.6379, found 1012.6410 (100 %). UV-vis ($1.13\cdot10^{-5}$ M, THF): λ_{max} (ε in $M^{-1}cm^{-1}$) = 277 (67600), 313 (47000), 331 (33300), 438 (15600) nm. IR (ATR): ν = 2935 (m, C-H), 2864 (m, C-H), 2110 (w, C≡C), 1507 (s), 1373 (s), 1258 (s), 996 (s), 797 (s), 673 (s) cm^{-1}.

Crystal data for $C_{56}H_{102}N_{12}Si_2Cu_2O_8\cdot4(C_2H_3N)$: M_r = 1418.97, 0.29 × 0.28 × 0.18 mm^3, triclinic, space group $P\bar{1}$, a = 10.7704(10), b = 14.1329(12), c = 14.6383(11) Å, α = 104.953(3), β = 97.771(3), γ = 111.910(3)°, V = 1929.5(3) $Å^3$, Z = 1, d_{calc} = 1.221 Mg m^{-3}, Mo K_α radiation (graphite monochromated, λ = 0.71073 Å), T = 102 K, θ_{range} 2.54

to 29.13°, reflections measured: 117076, indep: 10370, R_{int} = 0.0306, final R indices [I >2σ(I)]: R_1 = 0.0274, wR_2 = 0.0747

Figure S17: UV-vis spectrum (THF) of [**2a**(Cu(OAc)$_2$)$_2$]

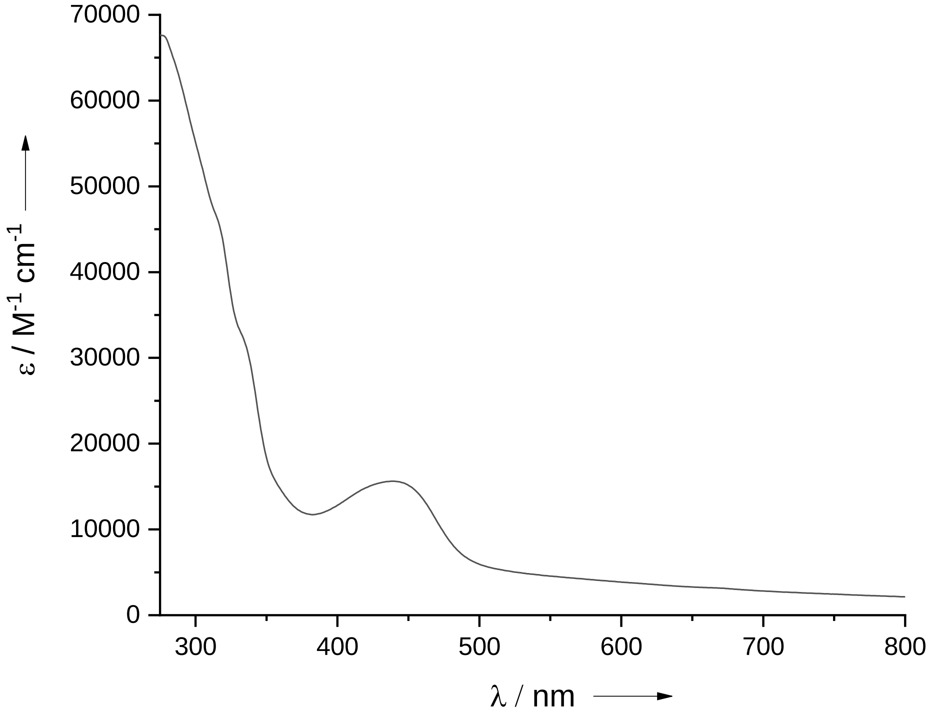

Figure S18: IR spectrum (ATR) of [**2a**(Cu(OAc)$_2$)$_2$]

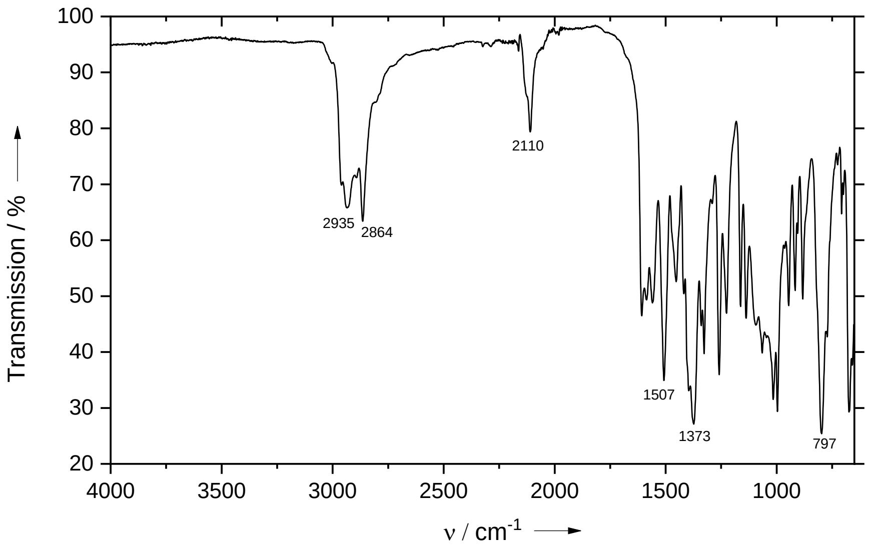

Figure S19: Cyclic voltammogram of [**2a**(Cu(OAc)$_2$)$_2$]

Solution of [**2a**(Cu(OAc)$_2$)$_2$] (1 mM) in CH_2Cl_2, Ag/AgCl reference electrode, with 0.1 M N(*n*Bu)$_4$PF$_6$ as supporting electrolyte, measured at a scan rate of 50 mV s^{-1}. Potentials referenced to the ferrocenium/ferrocene (Fc$^+$/Fc) redox couple.

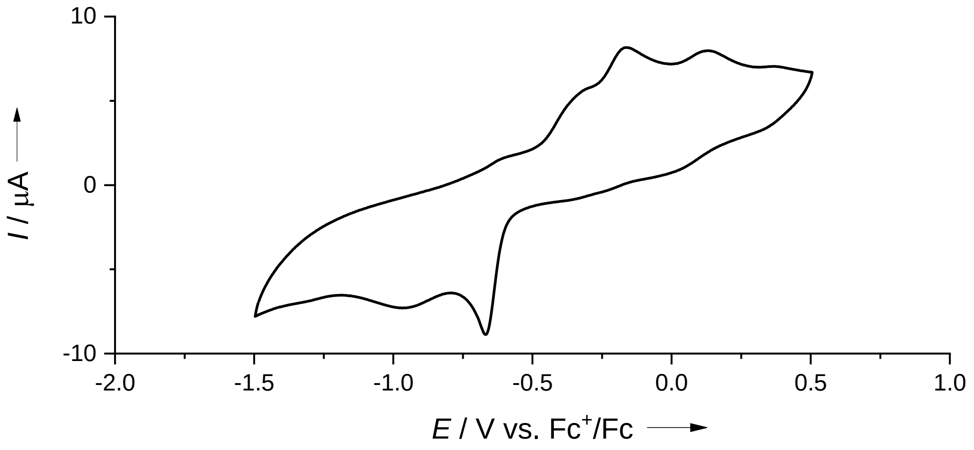

2.6) Complexation of 1,2,4,5-tetrakis(tetramethylguanidino)-3,6-bis((triisopropylsilyl)ethynyl)-benzene (**2a**) with CuI to give [**2a**(CuI)$_2$]

Synthesis

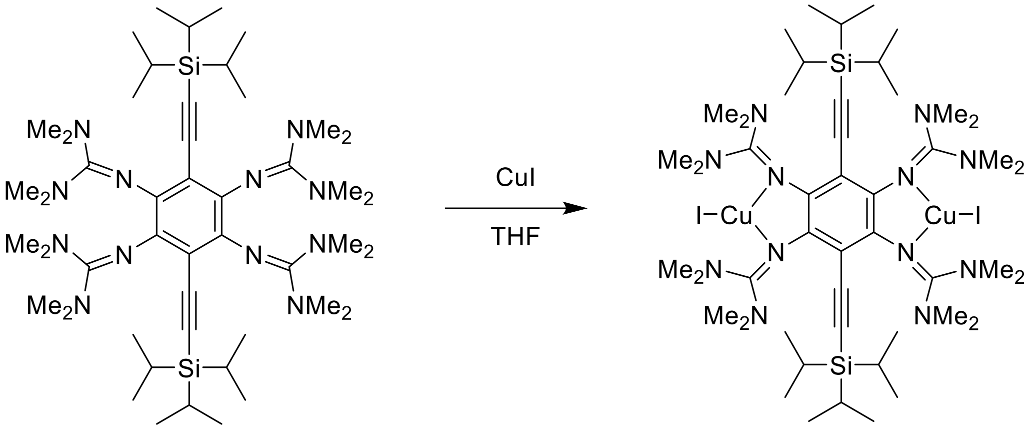

2a [**2a**(CuI)$_2$]

THF (7 ml) is added to CuI (21.4 mg, 112 µmol, 2 eq) and **2** (50.0 mg, 56 µmol, 1 eq). The reaction mixture is stirred at room temperature for 65 h, resulting in a green suspension. The solvent is filtered off and the precipitate is washed with CH_3CN (3 x 5 ml). The product is dried under reduced pressure, yielding 58.4 mg (46 µmol, 82 %). Yellow crystals suitable for XRD were obtained by diffusion of hexane into a solution of [**2a**(CuI)$_2$] in THF. Elemental analysis for $C_{48}H_{90}N_{12}Cu_2I_2Si_2$: calcd. C 45.31, H 7.13, N 13.21, Cu 9.99, I 19.95, Si 4.41; found C 45.52, H 7.31, N 12.91. ^{1}H NMR (600.13 MHz, CD_2Cl_2): δ = 2.84 (s, 48 H, CH_3), 1.10 (m, 42 H $CH(CH_3)_2$) ppm. ^{13}C NMR (150.90 MHz, CD_2Cl_2): δ = 40.4 (N-CH_3), 19.2 (CH_3), 12.7 (CH) ppm. HR-MS (ESI): $[M+H]^+$ calcd. (m/z) 1271.3704, found 1271.3805 (1%), $[M\text{-}CuI+H]^+$ calcd. 1081.5363, found 1081.5381 (100%), $[M\text{-}2CuI+H]^+$ calcd. 891.7023, found 891.7039 (47%). UV-vis ($4.5\cdot10^{-5}$ M, dichloromethane): λ_{max} (ε in $M^{-1}cm^{-1}$) = 236 (38900), 280 (17800, shoulder), 325 (9600), 342 (9300), 447 (10400) nm. IR (KBr): ν = 2939 (m, C-H), 2865 (m, C-H), 2119 (m, C≡C), 1521 (s, C=N), 1465 (m), 1419 (s), 1407 (s), 1387 (s), 1155 (m) cm^{-1}.

Crystal data for $C_{48}H_{90}N_{12}Si_2Cu_2I_2$: M_r = 1272.37, 0.50 × 0.25 × 0.15 mm^3, triclinic, space group $P\bar{1}$, a = 9.3390(19), b = 11.092(2), c = 15.089(3) Å, α = 86.77(3), β = 72.18(3)°, γ = 88.47(3), V = 1485.6(6) Å^3, Z = 1, d_{calc} = 1.422 Mg m^{-3}, Mo K$_\alpha$ radiation (graphite monochromated, λ = 0.71073 Å), T = 120 K, θ_{range} 2.266 to 30.122°,

reflections measured: 25190, indep: 8691, R_{int} = 0.0684, final R indices [I >2σ(I)]: R_1 = 0.0386, wR_2 = 0.0835.

Figure S20: ^{1}H NMR spectrum (600.13 MHz, CD_2Cl_2) of [**2a**(CuI)$_2$]

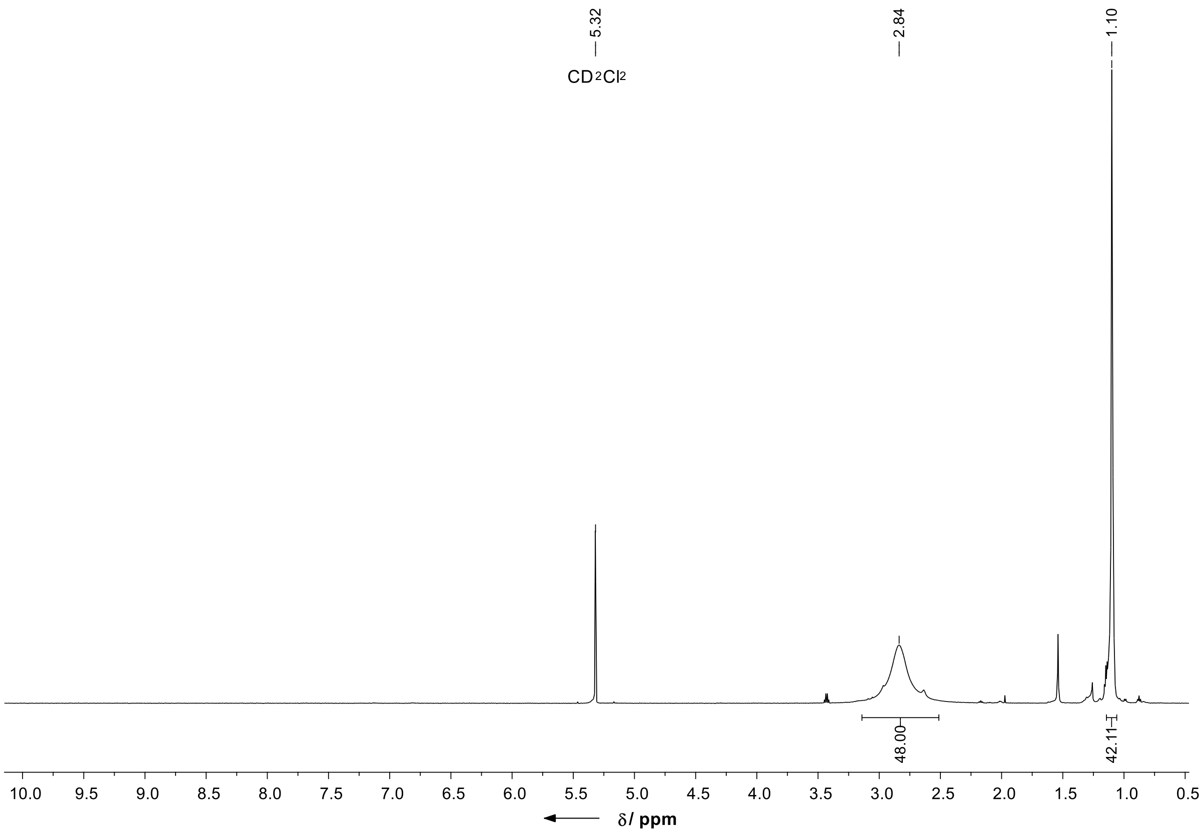

Figure S21: ^{13}C NMR (150.90 MHz, CD_2Cl_2) of [**2a**(CuI)$_2$]

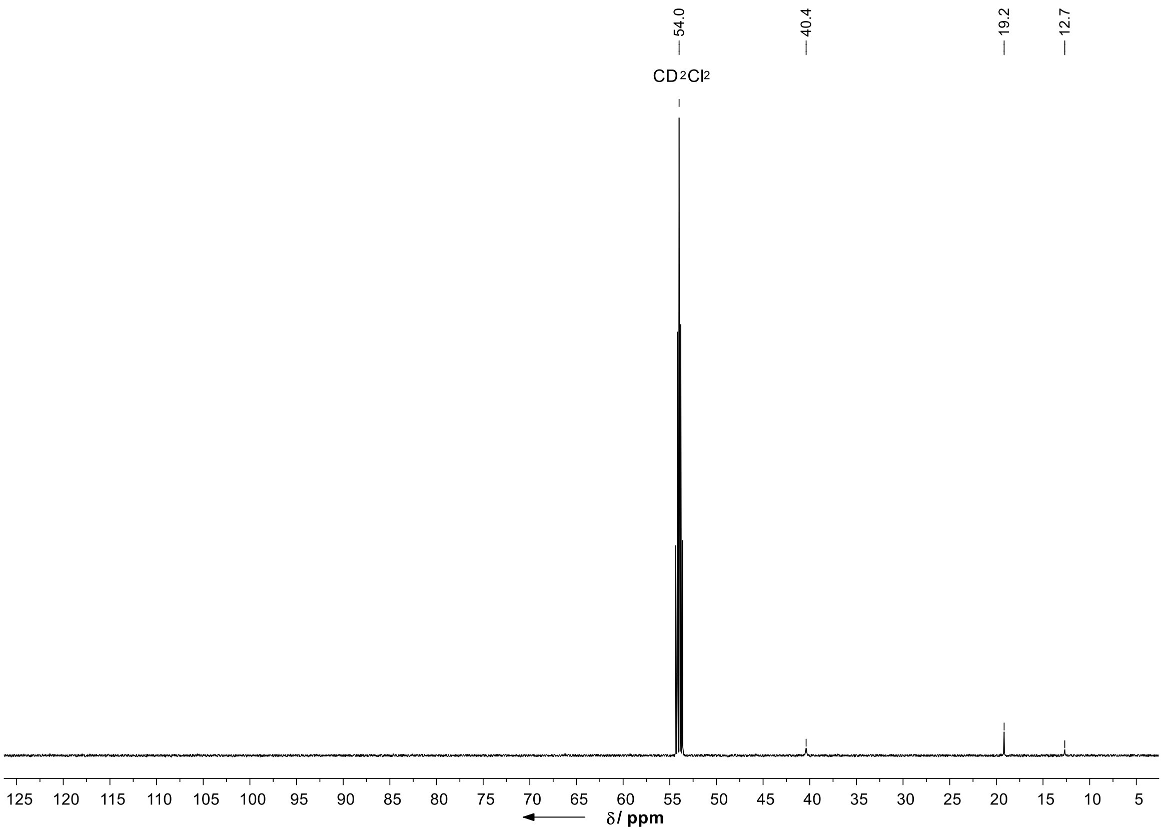

Figure S22: UV-vis spectrum (CH_2Cl_2) of [**2a**(CuI)$_2$]

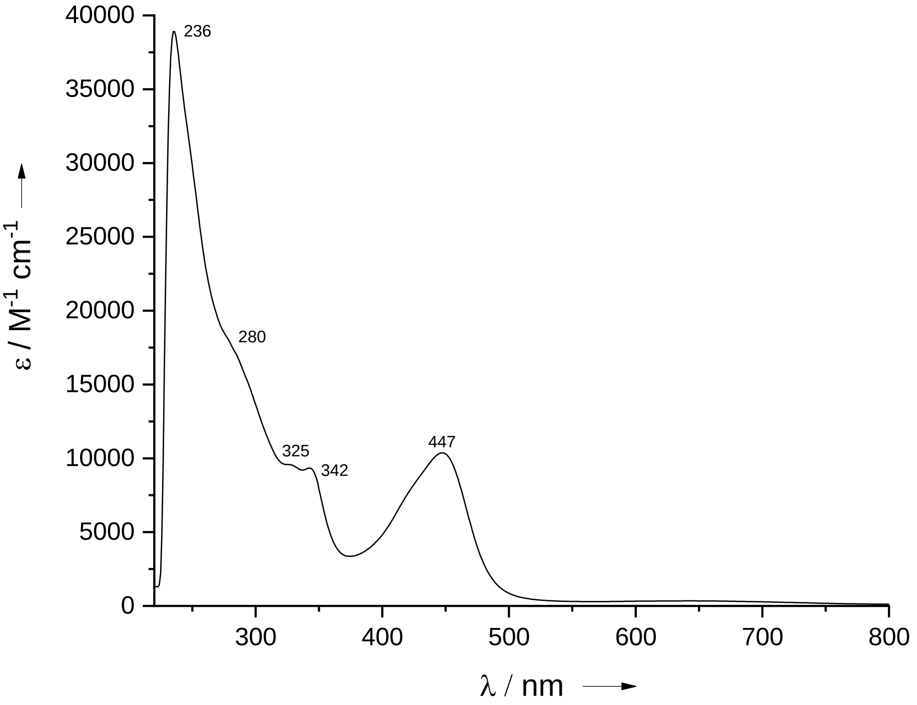

Figure S23: IR spectrum (KBr pellet) of [**2a**($CuI)_2$]

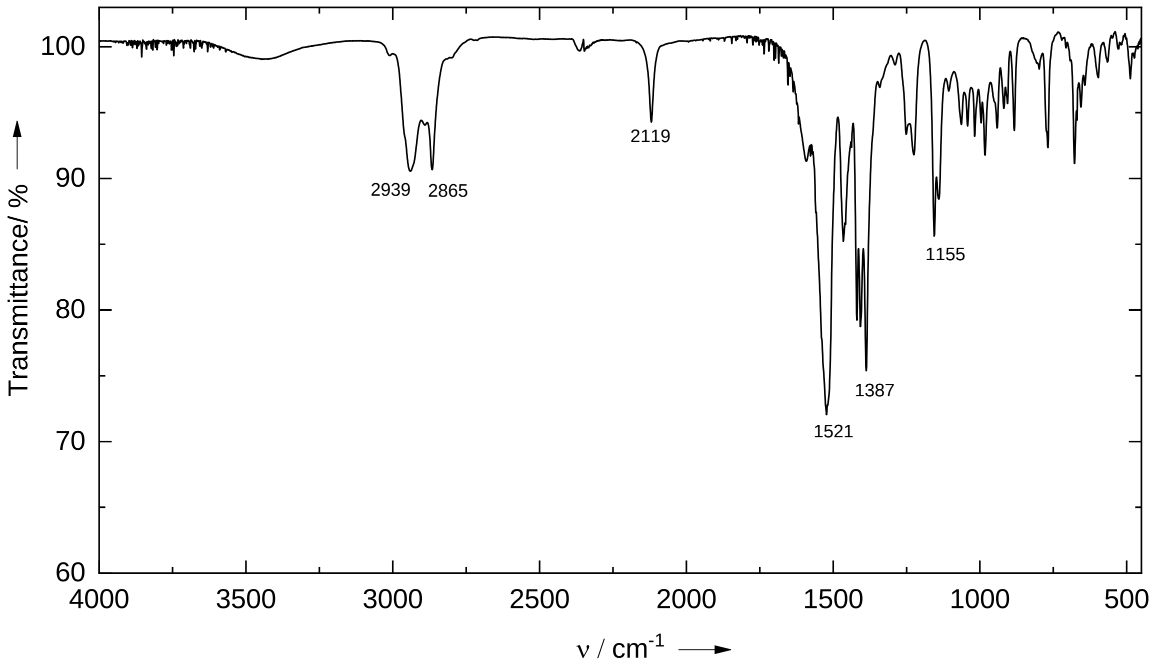

2.7) Oxidation of the complex **2a**($CuI)_2$ with iodine to give **2a**$^{2+}(I_3)_2$

Synthesis

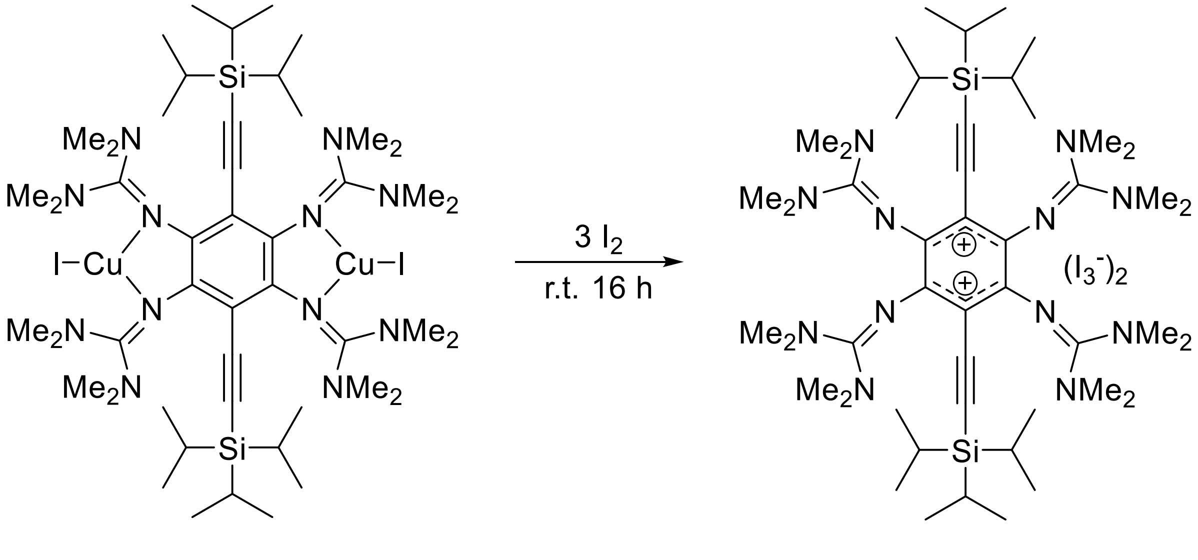

[**2a**(CuI)$_2$] **2a**($I_3)_2$

Complex **3**(CuI)$_2$ (58.4 mg, 45.9 µmol, 1 eq), iodine (36.9 mg, 137.7 µmol, 3 eq) and dichloromethane (5 ml) were stirred for 16 h at room temperature, resulting in a deep green solution. Removal of the solvent under reduced pressure gives a dark oil, which is dissolved in acetonitrile. Dark crystals of **2**$^{2+}$ with I_3^- as counterions were obtained by slow diffusion of diethyl ether into the acetonitrile solution. Yield: 42 mg (25.4 µmol, 55 %). Elemental analysis for $C_{48}H_{90}N_{12}I_6Si_2$: calcd. C 34.88, H 5.49, N 10.17, I 46.07, Si 3.40; found C 34.59, H 5.35, N 10.18. UV-vis ($1.0\cdot10^{-5}$ M, acetonitrile): λ_{max} (ε in $M^{-1}cm^{-1}$) = 211 (71000), 254 (62000), 290 (110000), 348 (56600), 443 (40500) nm. IR (KBr Pellet): ν = 2942 (m, C-H), 2863 (m, C-H), 2129 (w, C≡C), 1506 (s, C=N), 1329 (s), 961 (m), 777 (m) cm^{-1}.

Crystal data for $C_{48}H_{90}N_{12}Si_2I_6$: M_r = 1652.89, 0.50 × 0.20 × 0.15 mm^3, triclinic, space group $P\bar{1}$, *a* = 12.901(3), *b* = 15.182(3), *c* = 16.818(3) Å, α = 92.40(3), *β* = 90.45(3), γ = 101.66(3)°, *V* = 3222.7(12) Å^3, *Z* = 2, d_{calc} = 1.702 Mg m^{-3}, Mo K_α radiation (graphite monochromated, *λ* = 0.71073 Å), *T* = 120 K, θ_{range} 1.212 to 28.999°, reflections

measured: 51687, indep: 8578, R_{int} = 0.1177, final R indices [I >2σ(I)]: R_1 = 0.0433, wR_2 = 0.1158.

<u>Figure S24</u><u>: UV-vis (CH_3CN) of **2a**$^{2+}$(I_3)$_2$</u>

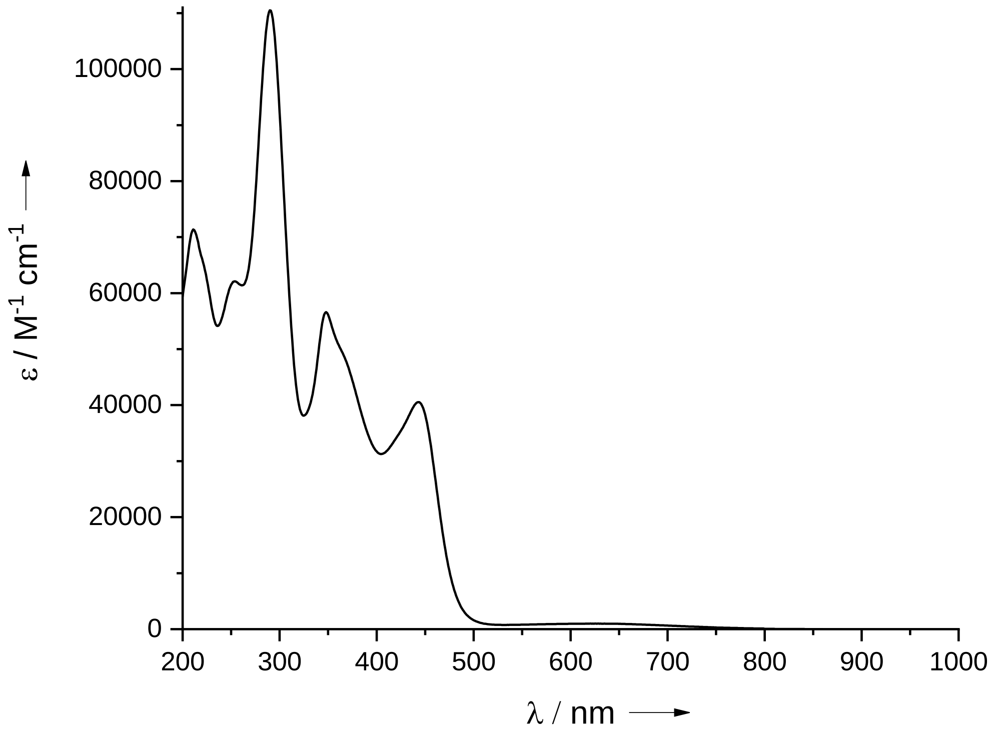

<u>Figure S25</u><u>: IR spectrum (KBr pellet) of **2a**$^{2+}$(I_3)$_2$</u>

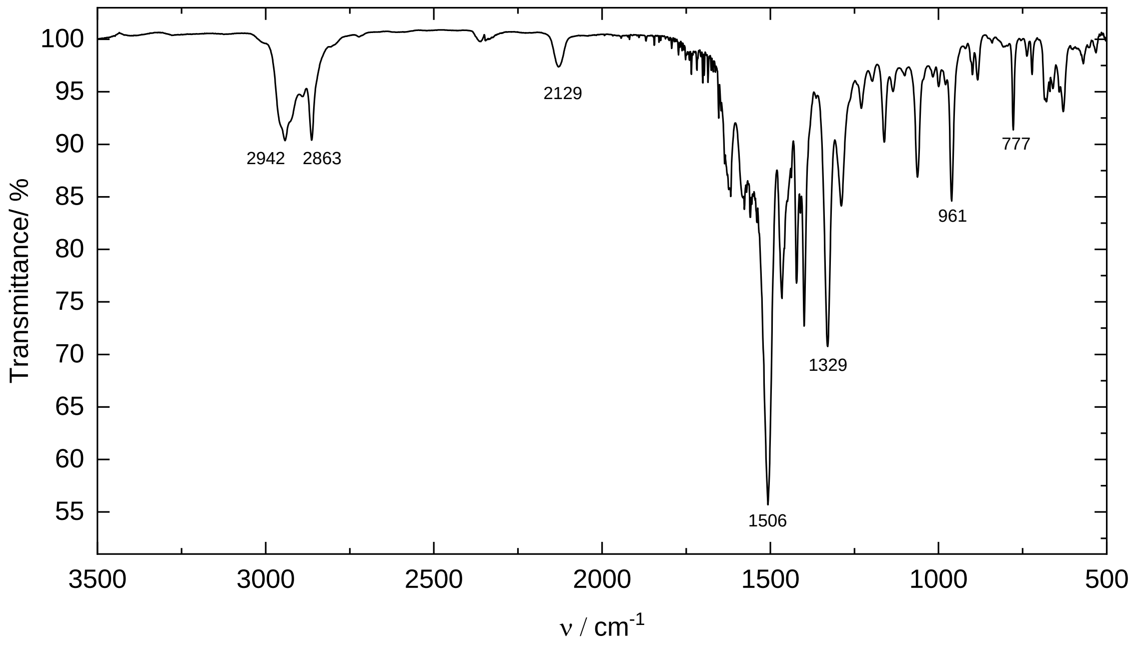

2.8) Complexation of 1,2,4,5-tetrakis(tetramethylguanidino)-3,6-bis(ethynyl)-benzene (**3**) with CuI to give the complex [**3**(CuI)$_2$]

Synthesis

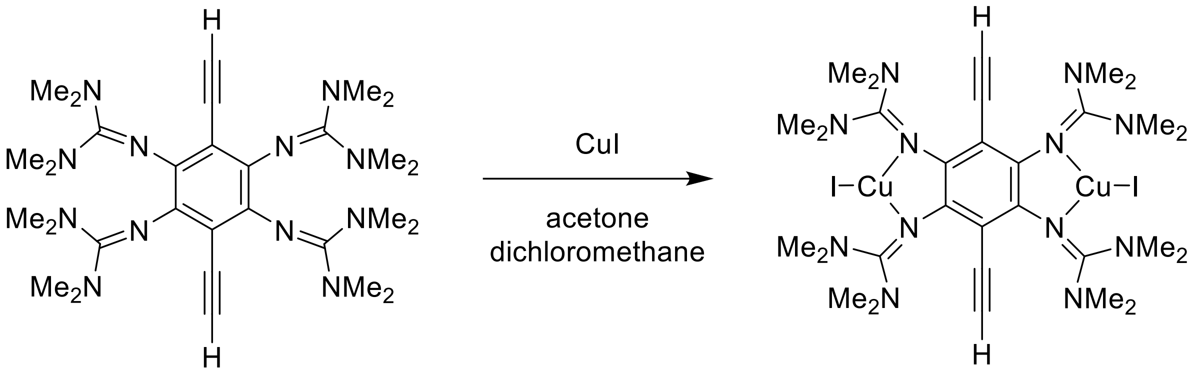

3 [**3**(CuI)$_2$]

To **3** (20 mg, 0.035 mmol, 1 eq.) and CuI (13 mg, 0.070 mmol, 2 eq.) acetone (4 ml) and dichloromethane (1.5 ml) is added. The yellow solution is stirred at room temperature for 2 h and stored at –20 °C overnight, giving crystals suitable for XRD directly from the reaction mixture. The solution is filtered off and the residue is washed with diethyl ether (3 × 3 ml) to yield [**3**(CuI)$_2$] in the form of a yellow solid (21 mg, 0.022 mmol, 63 %). Elemental analysis for $C_{30}H_{50}N_{12}Cu_2I_2\cdot(CH_2Cl_2)$: calcd. C 35.64, H 5.02, N 16.09, Cu 12.17, I 24.30, Cl 6.79; found C 36.22, H 5.02, N 16.74. ^{1}H NMR (600.13 MHz, CD_2Cl_2): δ = 5.33 (s, 2 H, CH_2Cl_2, from crystals), 3.09 (s, 2 H, C≡CH), 2.85 (s, 48 H, CH_3) ppm. ^{13}C NMR (150.90 MHz, CD_2Cl_2): δ = 164.8 (NCN_2), 139.5 (C Ar-N), 105.9 (C Ar-CCH), 81.7 (C≡C), 80.7 (C≡C), 40.0 (CH_3) ppm. HR-MS (ESI): [M-CuI+H]$^+$ calcd. (m/z) 769.2695, found 769.2697 (13%), [M-Cu]$^+$ calcd. (m/z) 831.1918, found 831.2287 (46%), [M-(CuI)$_2$]$^+$ calcd. (m/z) 578.4281, found 578.4275 (100%). UV-vis ($1.9\cdot10^{-5}$ M, CH_2Cl_2): λ_{max} (ε in $M^{-1}cm^{-1}$) = 229 (59500), 294 (34000), 451 (8600) nm. IR (ATR): ν = 3228 (m, C≡C-H), 2933 (m, C-H), 2872 (m, C-H), 1515 (s, C=N), 1386 (s), 1152 (s) cm^{-1}.

Crystal data for $C_{30}H_{50}N_{12}Cu_2I_2\cdot(CH_2Cl_2)$: M_r = 1044.62, 0.104 × 0.77 × 0.71 mm^3, orthorhombic, space group *Pccn*, a = 14.841(3), b = 19.992(4), c = 13.952(3) Å, α = 90, β = 90, γ = 90°, V = 4139.6(15) Å^3, Z = 4, d_{calc} = 1.676 Mg m^{-3}, Mo K$_\alpha$ radiation (graphite monochromated, λ = 0.71073 Å), T = 100 K, θ_{range} 2.037 to 24.998°,

reflections measured: 137851, indep: 3653, R_{int} = 0.1216, final R indices [I >2σ(I)]: R_1 = 0.0379, wR_2 = 0.1158

Figure S26: ^{1}H NMR spectrum (600.13 MHz, CD_2Cl_2) of [**3**(CuI)$_2$]

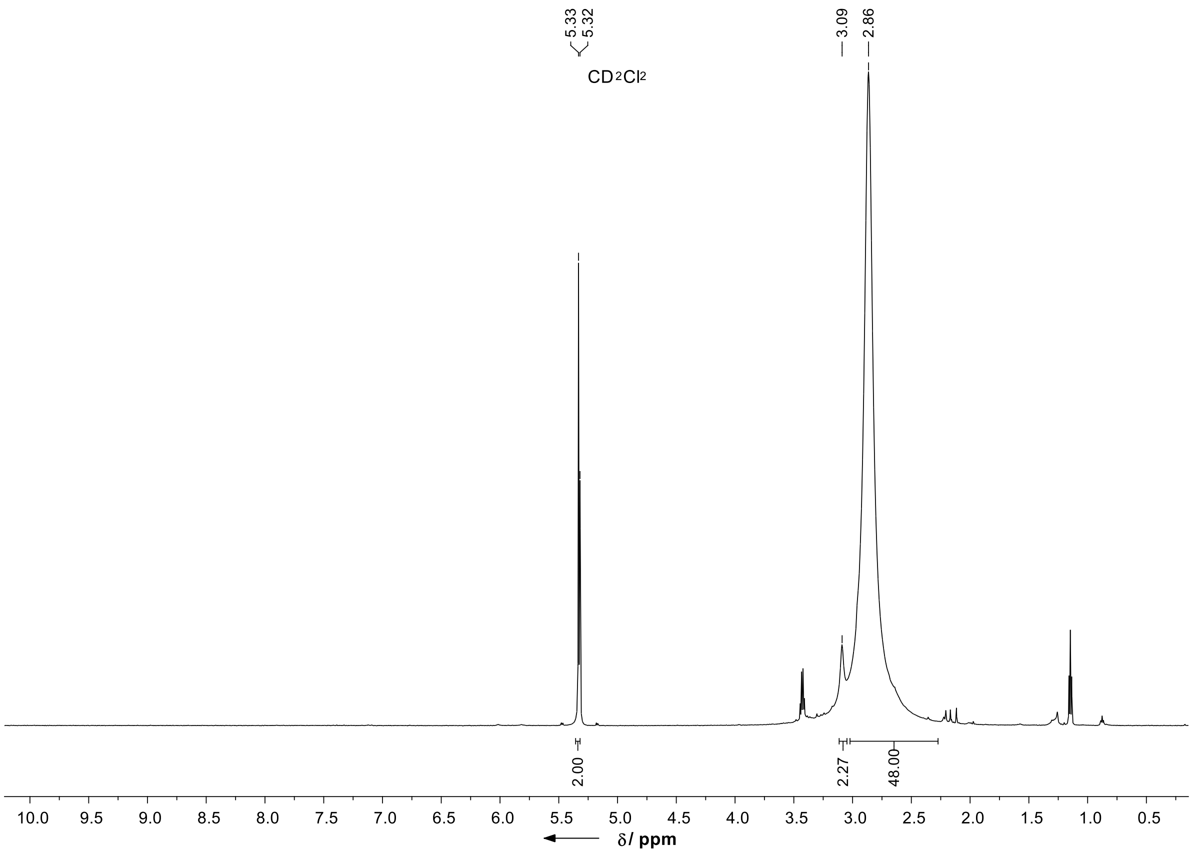

Figure S27: ^{13}C NMR spectrum (150.90 MHz, CD_2Cl_2) of [**3**(CuI)$_2$]

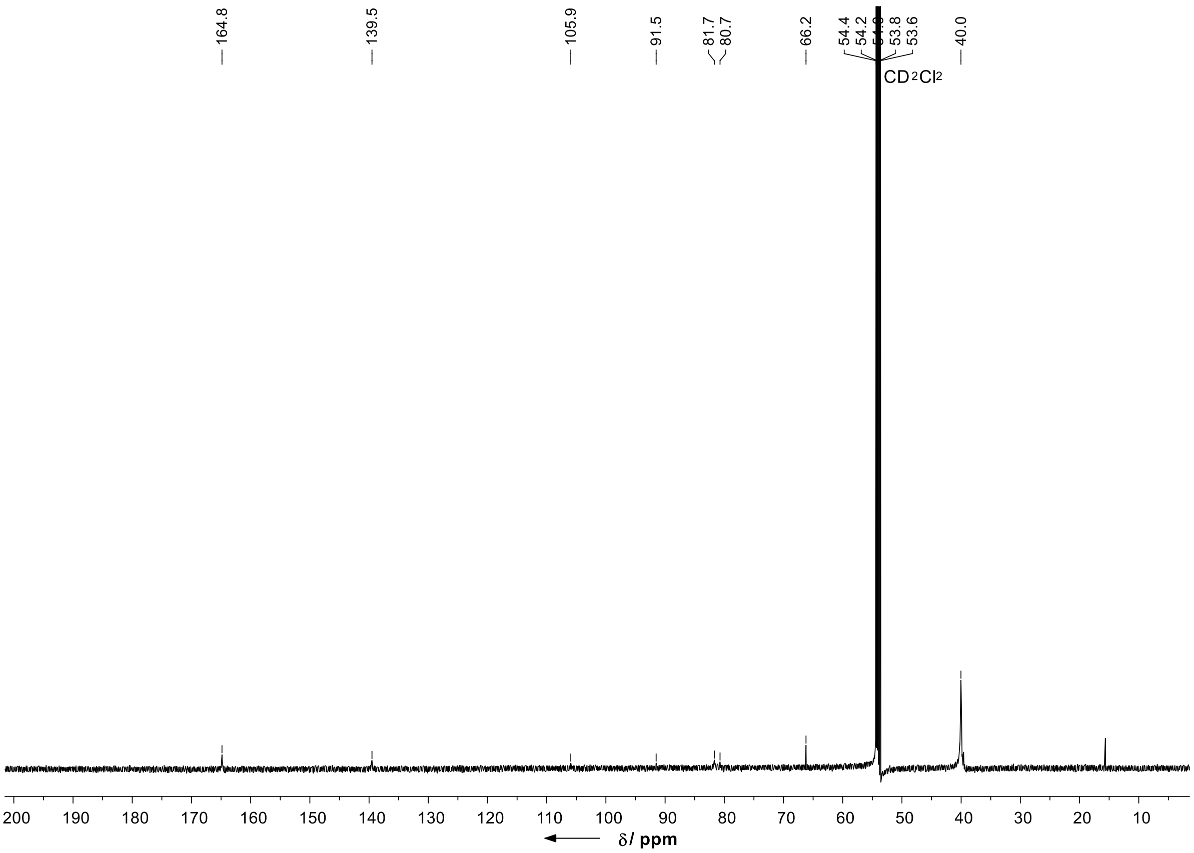

Figure S28: UV-vis spectrum (CH_2Cl_2) of [**3**(CuI)$_2$]

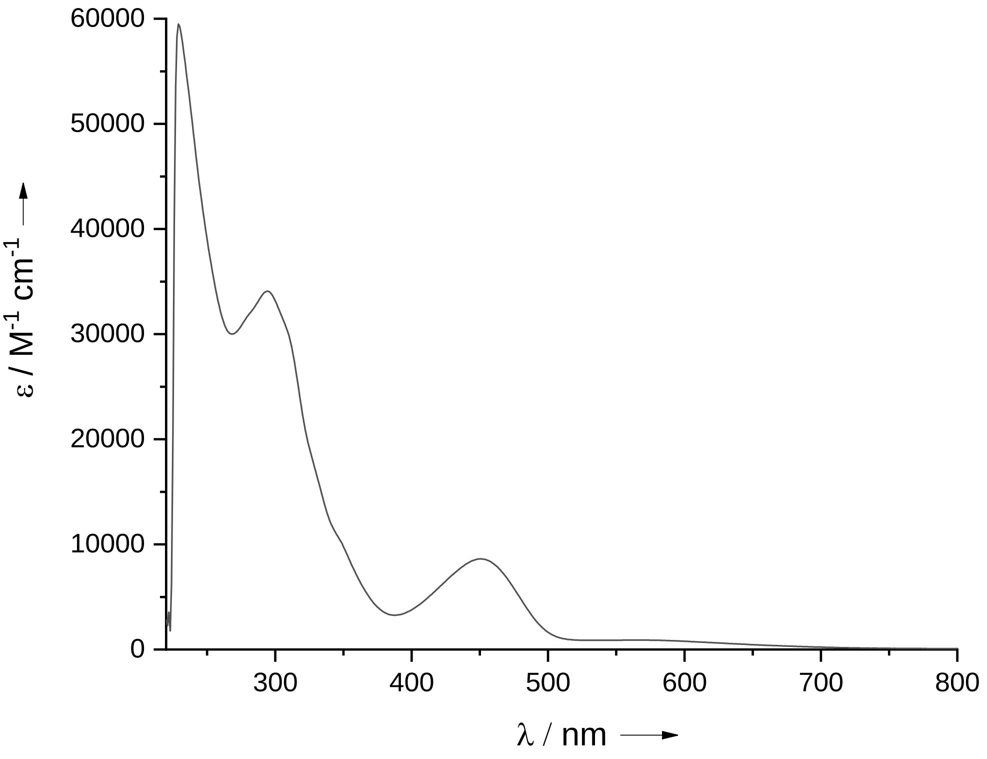

Figure S29: IR spectrum (ATR) of [**3**(CuI)$_2$]

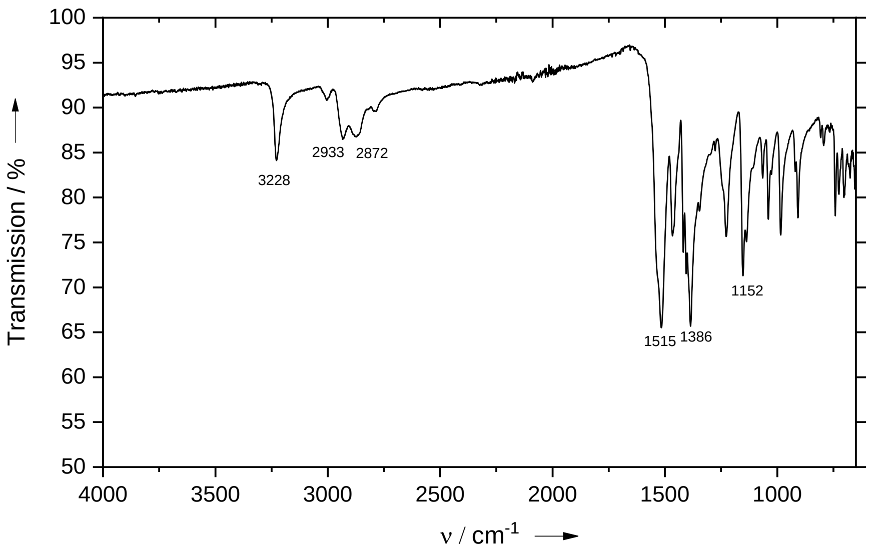

Figure S30: UV-vis spectra recorded for the reaction of [**3**(CuI)$_2$] with dioxygen

The characteristic transition of [**3**(CuI)$_2$] at 451 nm disappears upon exposure to dioxygen. A dark solid, insoluble in standard organic solvents, precipitates from solution.

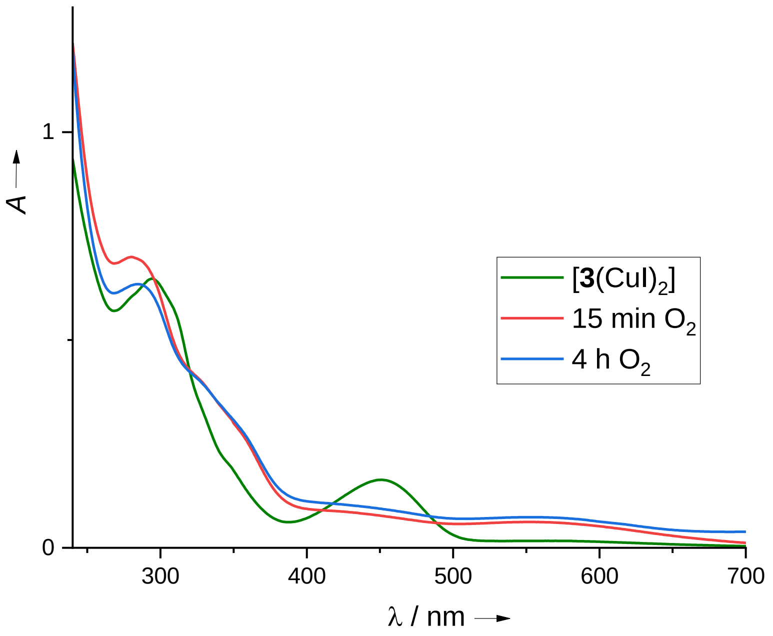

2.9) Complexation of 1,2,4,5-tetrakis(tetramethylguanidino)-3,6-bis(ethynyl)-benzene (**3**) with $Cu(OAc)_2$ to give [**3**$(Cu(OAc)_2)_2$]

Synthesis

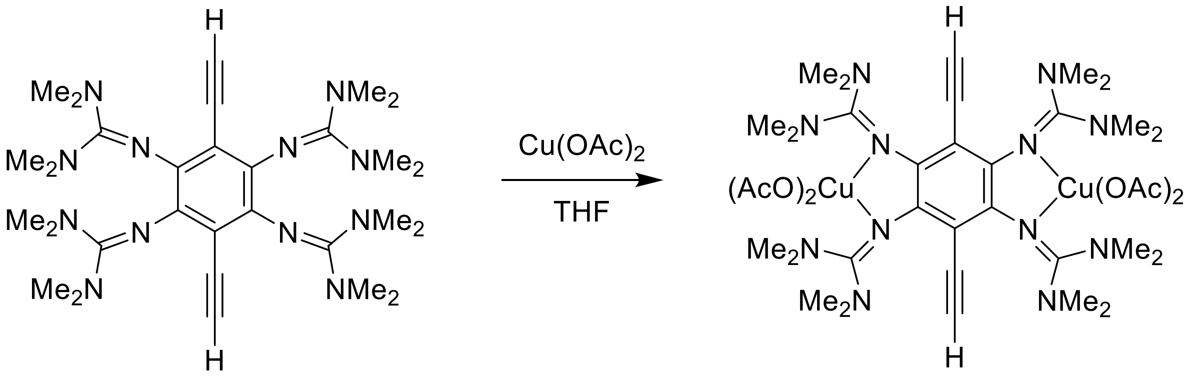

To **3** (20 mg, 0.035 mmol, 1 eq.) and $Cu(OAc)_2$ (12.5 mg, 0.070 mmol, 2 eq.) THF (5 ml) is added. The solution turns brown and is stirred at r.t. for 6 h. Crystals suitable for XRD were obtained directly from the reaction mixture after storage at –20 °C overnight. The solvent is removed under reduced pressure and the residue is washed with pentane (2 × 2 ml) and diethyl ether (3 × 2 ml). Yield: 18 mg (0.019 mmol, 55 %). Elemental analysis for $C_{38}H_{62}N_{12}Cu_2O_8$: calcd. C 48.45, H 6.63, N 17.84, Cu 13.49, O 13.59; found C 48.58, H 6.57, N 17.91. HR-MS (ESI): $[M-Cu(OAc)_3]^+$ calcd. (m/z) 700.3710, found 700.3713 (100%). UV-vis ($1.85 \cdot 10^{-5}$ M, THF): λ_{max} (ε in $M^{-1}cm^{-1}$) = 290 (33800), 350 (9600), 452 (8300) nm. IR (ATR): ν = 3304 (w, C≡C-H), 2927 (m, C-H), 2088 (w, C≡C), 1508 (s, C=N), 1379 (s), 1327 (s), 1160 (s), 994 (s), 673 (s) cm^{-1}.

Crystal data for $C_{38}H_{62}N_{12}Cu_2O_8 \cdot 2(C_4H_8O)$: M_r = 1086.28, 0.18 × 0.17 × 0.12 mm^3, monoclinic, space group $P2_1/n$, a = 17.0480(18), b = 16.536(17), c = 18.512(2) Å, α = 90, β = 90.629(4), γ = 90°, V = 5211.2(10) $Å^3$, Z = 4, d_{calc} = 1.385 Mg m^{-3}, Mo K_α radiation (graphite monochromated, λ = 0.71073 Å), T = 100 K, θ_{range} 2.74 to 28.26°, reflections measured: 287303, indep: 12956, R_{int} = 0.0505, final R indices [$I > 2\sigma(I)$]: R_1 = 0.0402, wR_2 = 0.1028

Figure S31: UV-vis spectrum (THF) of [**3**($Cu(OAc)_2)_2$]

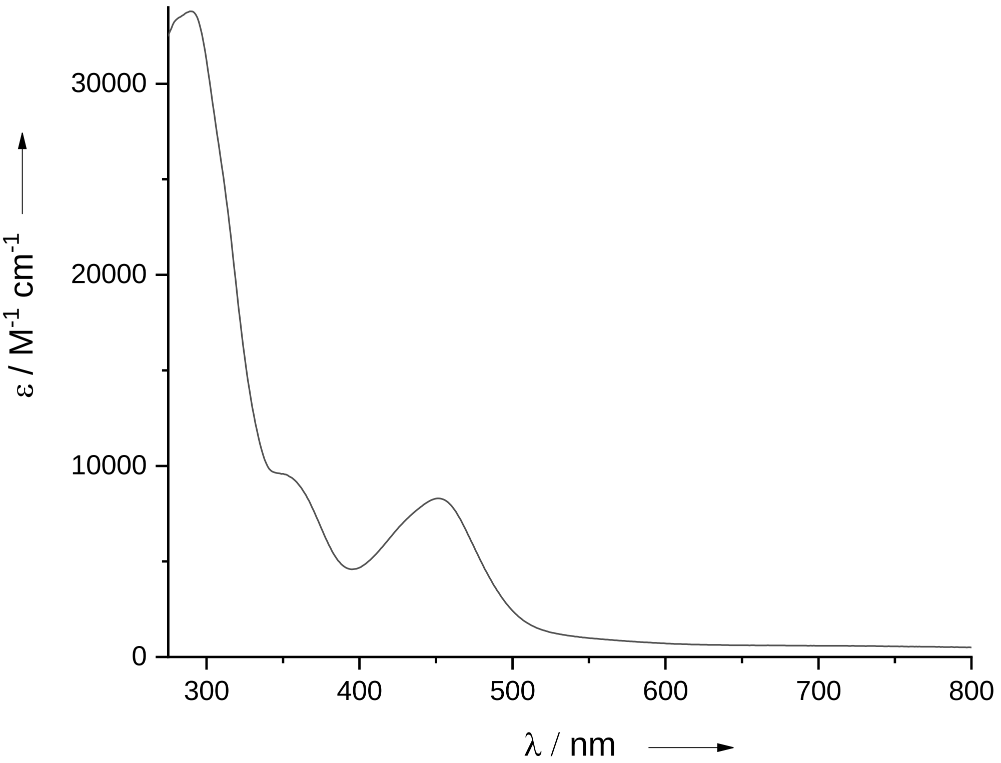

Figure S32: IR spectrum (ATR) of [**3**($Cu(OAc)_2)_2$]

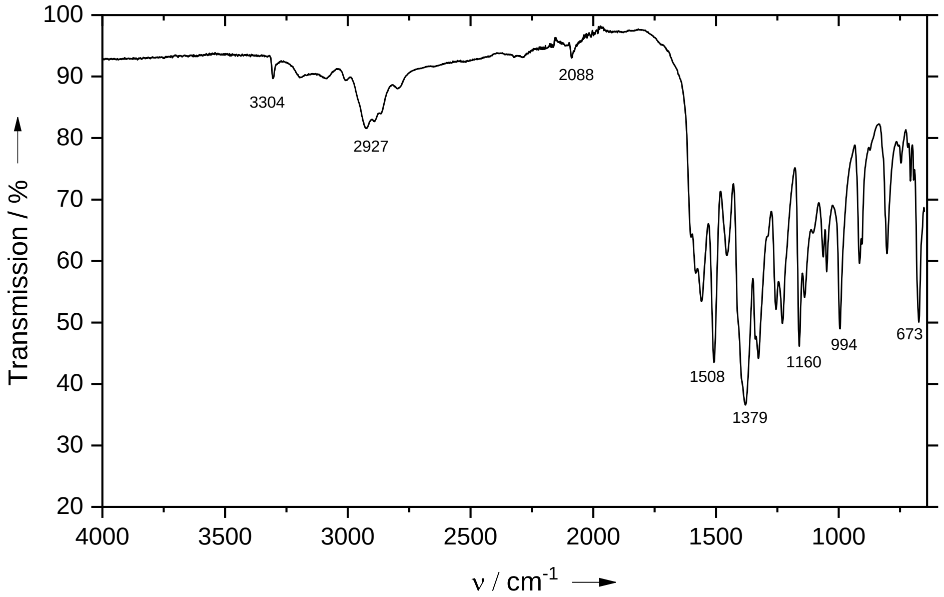

Figure S33: Cyclic voltammogram of [**3**(Cu(OAc)$_2$)$_2$]

Solution of [**3**(Cu(OAc)$_2$)$_2$] (1 mM) in CH_2Cl_2, Ag/AgCl reference electrode, with 0.1 M N(*n*Bu)$_4$PF$_6$ as supporting electrolyte, measured at a scan rate of 100 mV s^{-1}. Potentials referenced to the ferrocenium/ferrocene (Fc^+/Fc) redox couple.

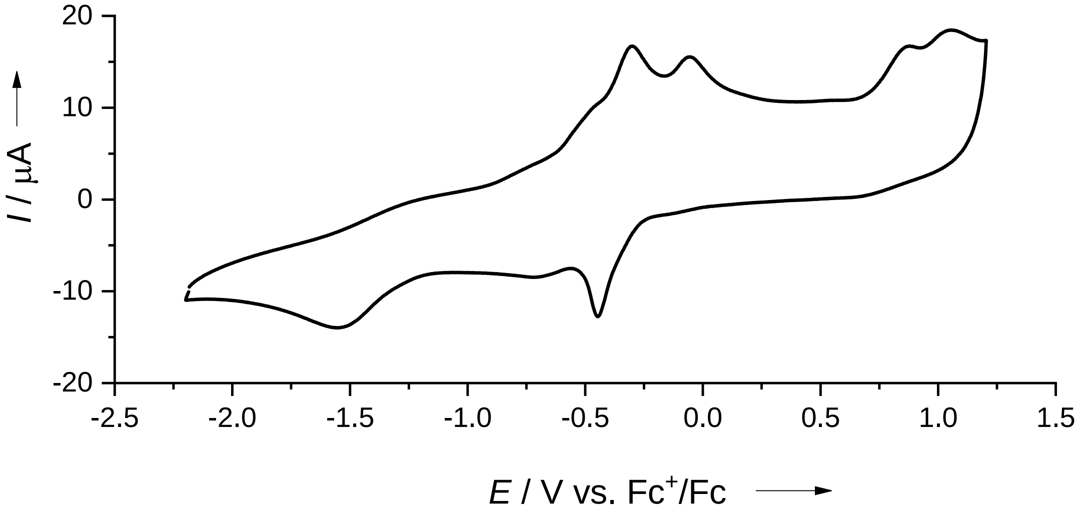

2.10) Addition of tris(pentafluorophenyl)borane to 1,2,4,5-tetrakis(tetramethylguanidino)-3,6-bis(ethynyl)-benzene (**3**) to give compound **4**

Synthesis

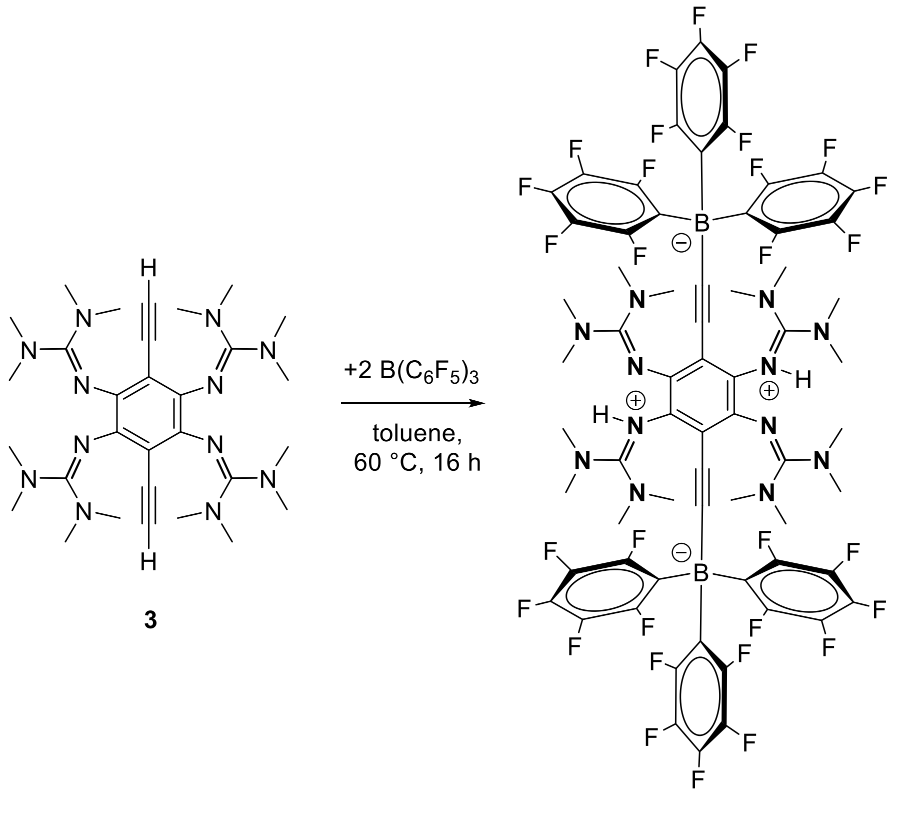

The starting reagents, 30 mg (0.052 mmol) of 1,2,4,5-tetrakis(tetramethylguanidino)-3,6-bis(ethynyl)-benzene (**3**) and 53 mg (0.104 mmol, 2.0 eq.) of tris(pentafluorophenyl)borane, are dissolved in toluene (5 ml). The reaction mixture is stirred at 60 °C for 16 h. An orange precipitate over yellow solution is formed. The solution is filtered off and the residue is washed with toluene (2 × 5 ml) and diethyl ether (2 × 4ml). The product is obtained as a yellow solid after drying under reduced pressured. Crystals suitable for XRD were obtained from a saturated dichloromethane solution at room temperature. Yield: 54 mg (0.034 mmol, 65 %). Elemental analysis for $C_{66}H_{50}B_2F_{30}N_{12}$: calcd. C 49.46, H 3.14, N 10.49, B 1.35, F 35.56; found C 48.55, H 3.60, N 10.43. ^{1}H NMR (399.89 MHz, acetone-d_6): δ = 3.02 (s, 24 H), 2.45 (s, 24 H) ppm. ^{11}B NMR (128.30 MHz, acetone-d_6): δ = –20.75 ppm. ^{19}F NMR (376.27 MHz,

acetone-d_6): δ = –132.4, –164.3, –167.8 ppm. Due to the low solubility, it was not possible to record the signals in the ^{13}C NMR spectrum. HR-MS (ESI): $[M+H]^+$ calcd. (m/z) 1603.4061, found 1603.4059 (100 %). UV-vis ($4.4 \cdot 10^{-5}$ M, dichloromethane): λ_{max} (ε in $M^{-1}cm^{-1}$) = 260 (20400), 299 (23600), 315 (20300), 401 (8300) nm. IR (KBr): ν = 3353 (w, N-H), 2927 (w, C-H), 1559 (s), 1457 (s), 1086 (m), 977 (m) cm^{-1}.

Crystal data for $C_{66}H_{50}B_2F_{30}N_{12} \cdot 4(CH_2Cl_2)$: M_r = 1942.50, 0.23 × 0.19 × 0.13 mm^3, triclinic, space group $P\bar{1}$, a = 12.1206(8), b = 13.7496(8), c = 14.4682(8) Å, α = 110.328(2), β = 90.630(3), γ = 113.604(2)°, V = 2040.0(2) $Å^3$, Z = 1, d_{calc} = 1.581 Mg m^{-3}, Mo K_α radiation (graphite monochromated, λ = 0.71073 Å), T = 120 K, θ_{range} 2.191 to 29.584°, reflections measured: 68628, indep: 11429, R_{int} = 0.0395, final R indices [$I > 2\sigma(I)$]: R_1 = 0.0486, wR_2 = 0.1471.

Figure S34: ^{1}H NMR spectrum (399.89 MHz, acetone-d_6) of **4**

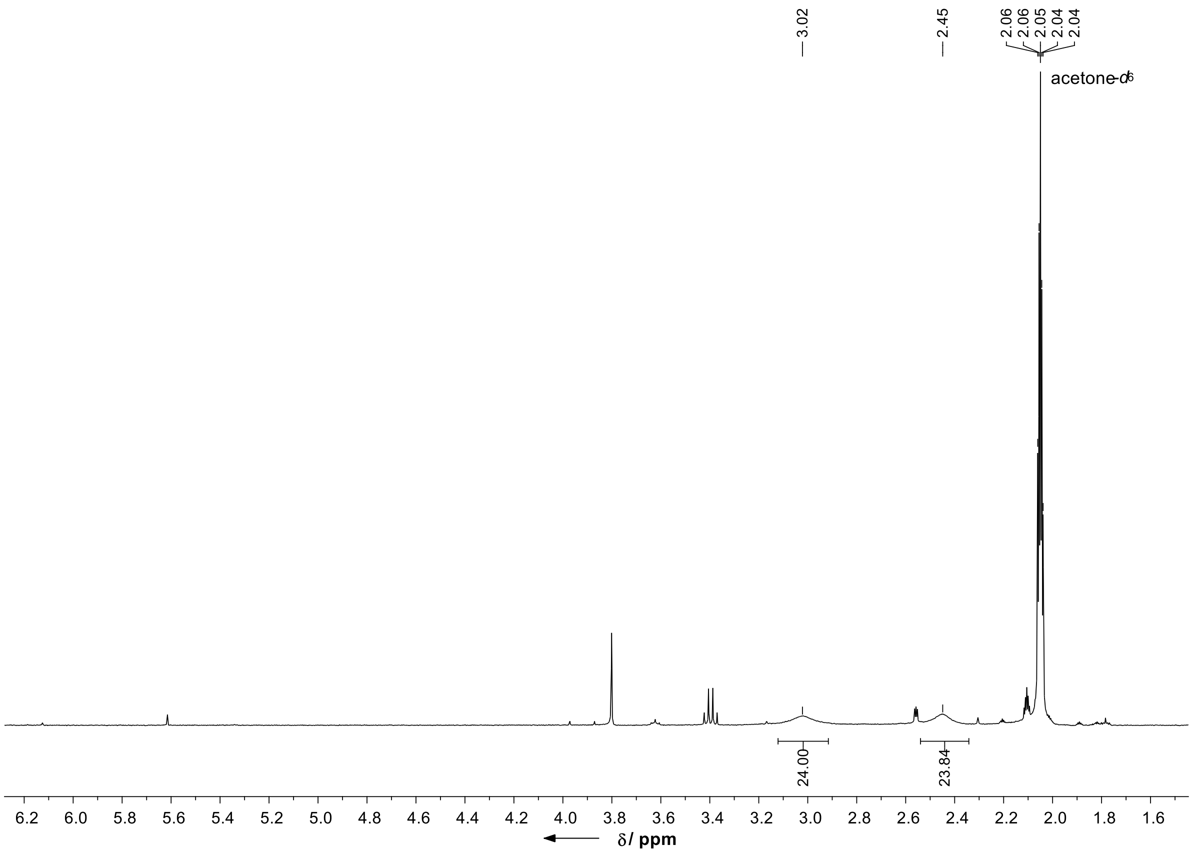

Figure S35: ^{11}B NMR spectrum (128.30 MHz, acetone-d_6) of **4**

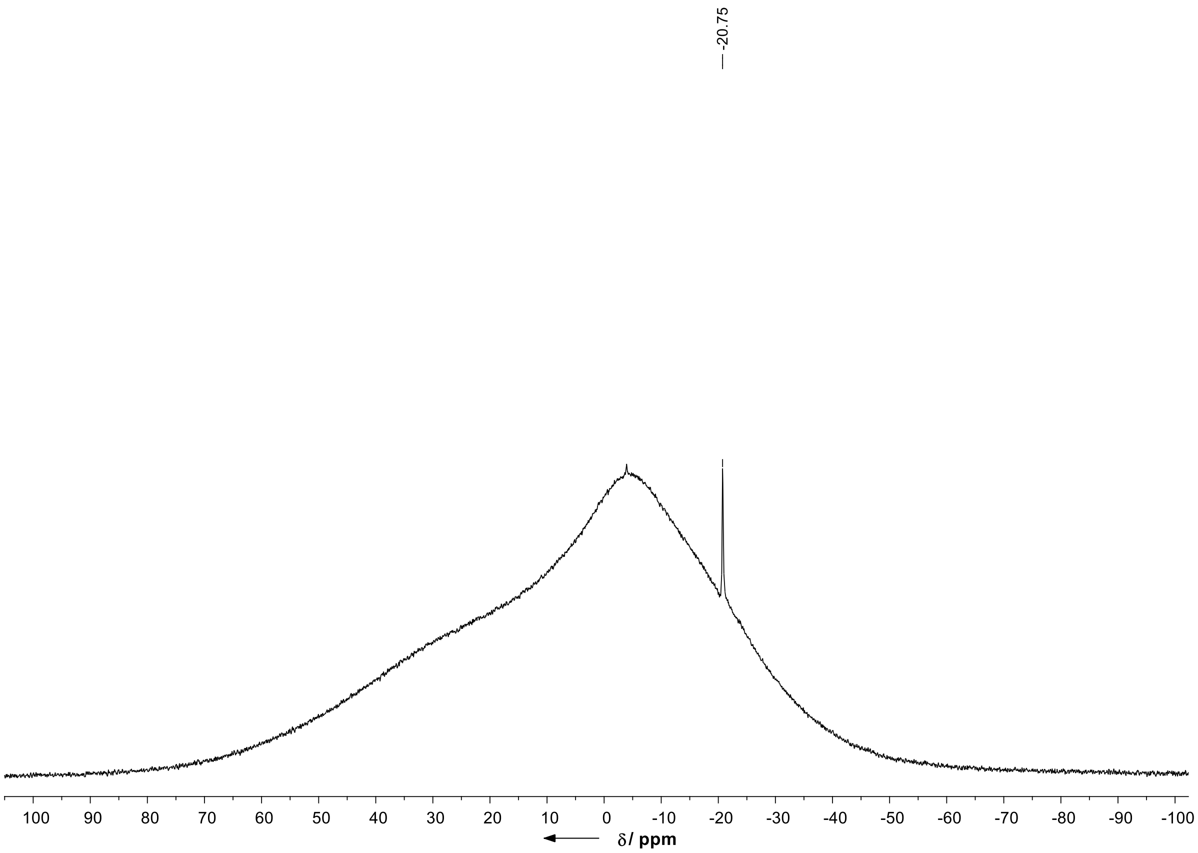

Figure S36: UV-vis spectrum (CH_2Cl_2) of **4**

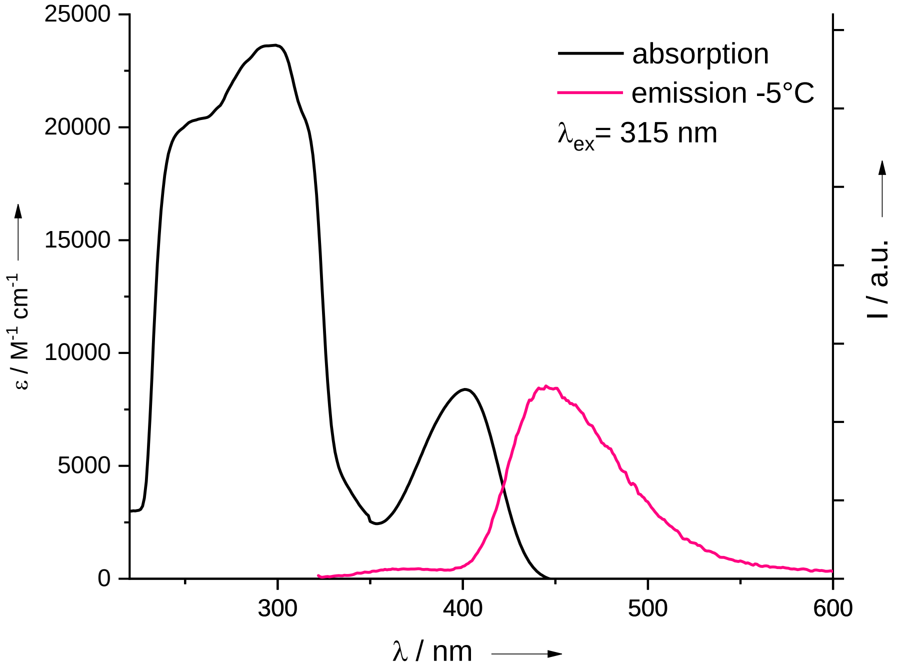

Figure S37: Temperature-dependent fluorescence spectra (CH_2Cl_2) of **4**, excited at 315 nm

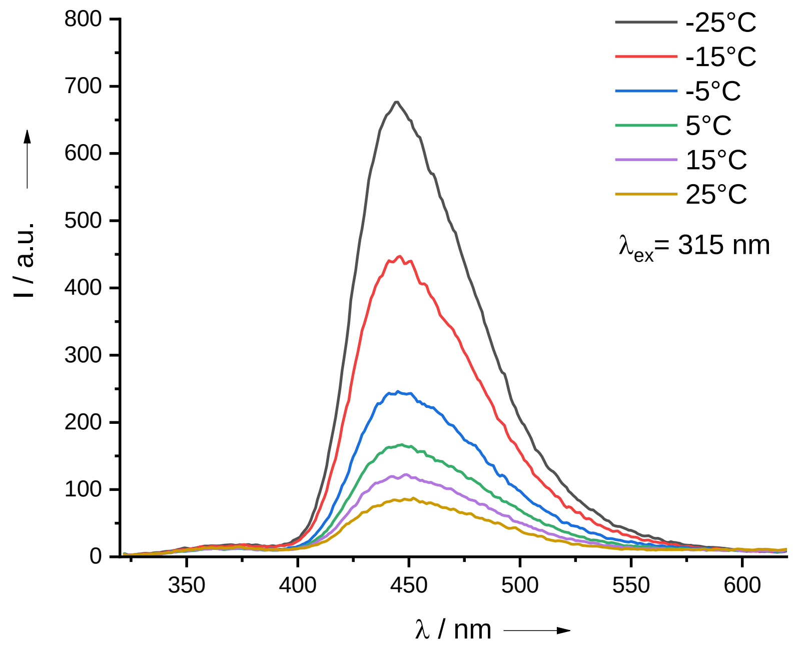

<u>Figure S38</u>: IR spectrum (KBr pellet) of **4**

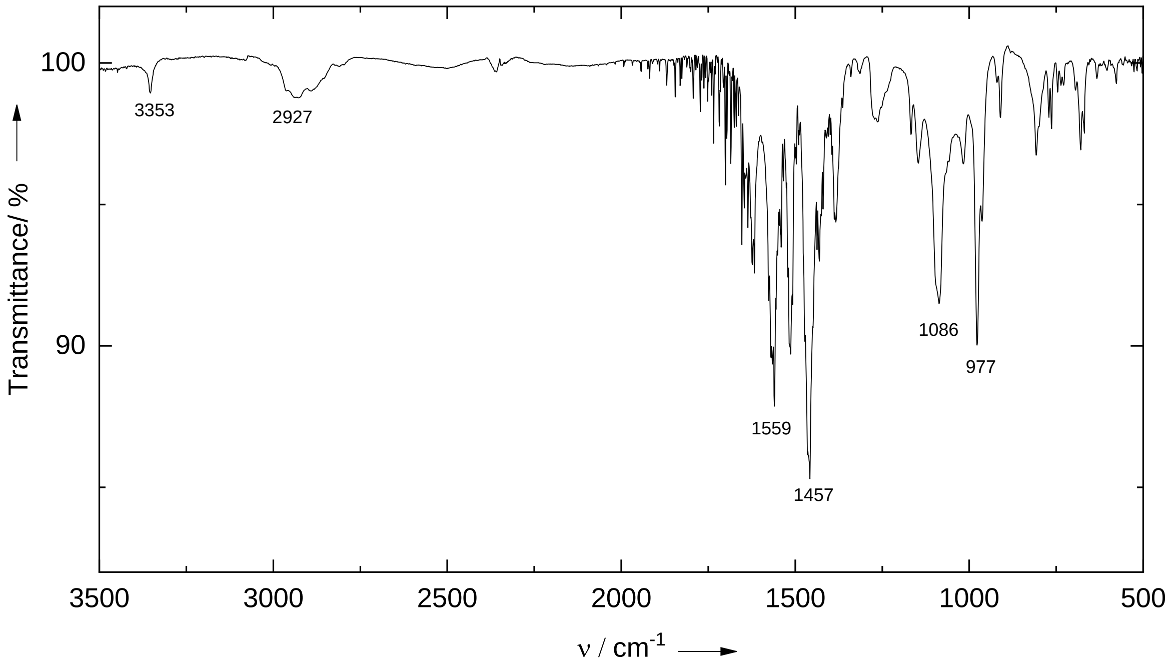

<u>Figure S39</u><u>: XRD solid state structure</u>

Illustration of the solid state structure of compound **4**. Methyl hydrogen atoms are omitted. Displacement ellipsoids are drawn at the 50 % probability level. Color code: C grey, N blue, B pink, F yellow, H green.

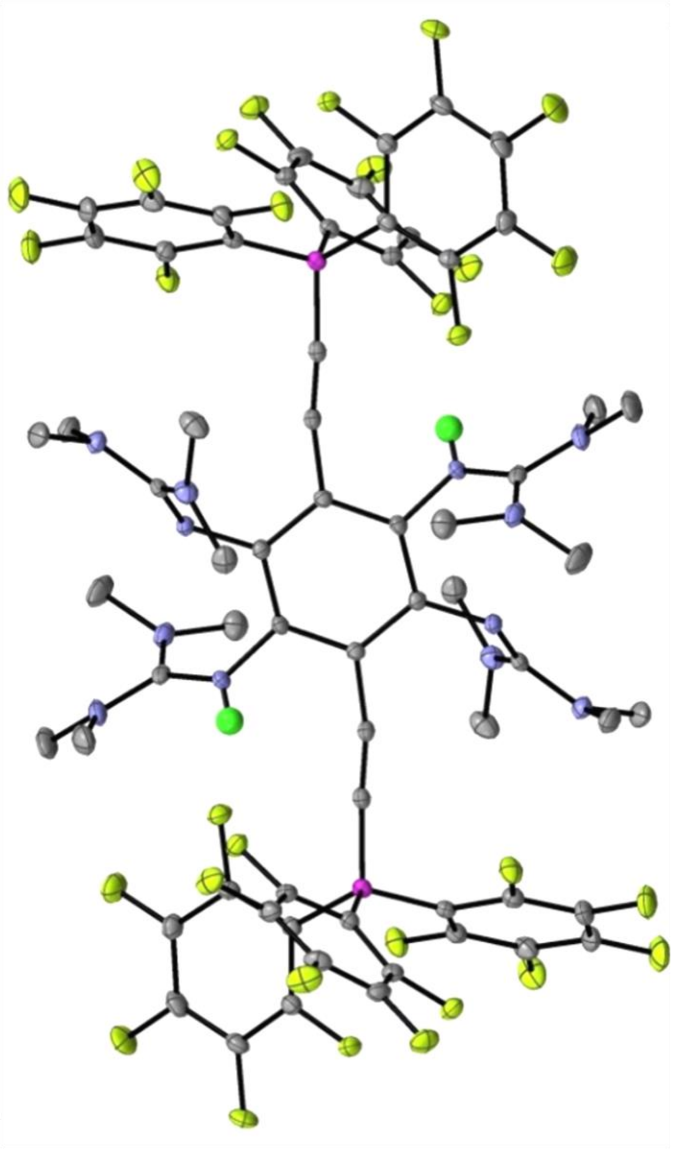

2.11) Oxidation of **4** with O_2 to give compound **5**

Synthesis

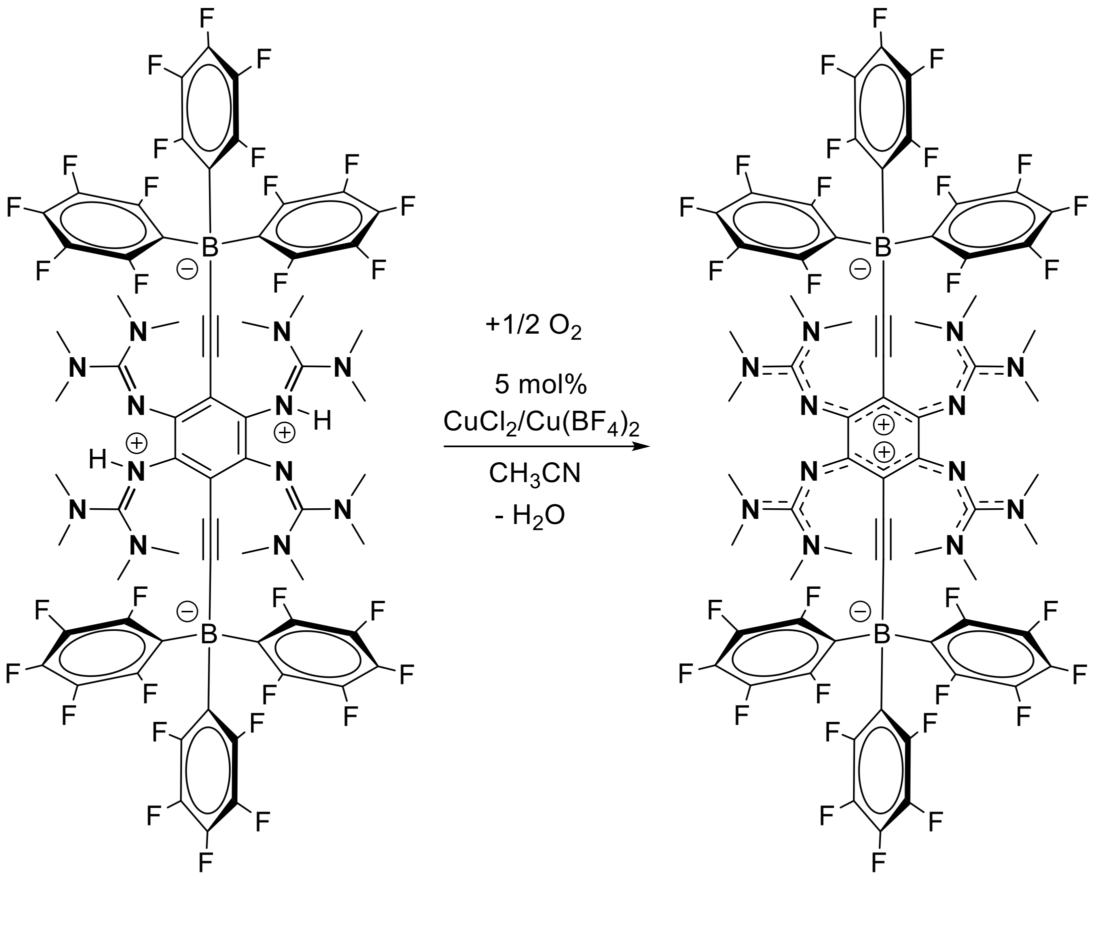

4 **5**

Dichloromethane (5 ml) and acetone (8 ml) are added to **4** (54 mg, 34 μmol), resulting in a yellow solution. Then, 0.4 ml of a solution of $Cu(BF_4)_2{\cdot}6H_2O$ in acetonitrile (18 mg in 10 ml) and 0.4 ml of a solution of $CuCl_2$ in acetonitrile (7 mg in 10 ml) are added, corresponding to an addition of 1.7 μmol (0.05 eq.) of the copper salts. The reaction mixture is saturated with O_2 gas, giving a green solution. The reaction mixture is stirred at 50 °C for 3 h. The solvent is removed under reduced pressure. The crude product is washed with diethyl ether (3 x 5 ml) and dichloromethane (2 x 1 ml). Crystals suitable for XRD could be obtained by slow diffusion of diethyl ether into an acetone solution. Yield 24 mg (15 μmol, 44 %). Elemental analysis for $C_{66}H_{48}B_2F_{30}N_{12}$: calcd. C 49.52, H 3.02, N 10.50, B 1.35, F 35.60; found C 49.94, H 3.28, N 10.68. ^1H NMR (600.13

MHz, THF-d_8): δ = 2.76 (s, 48 H) ppm. ^{13}C NMR (150.92 MHz, THF-d_8): δ = 150.2, 148.6, 140.1, 138.4, 136.7, 105.7, 40.1 (CH_3) ppm. ^{11}B NMR (128.30 MHz, THF-d_8): δ = –20.71 ppm. ^{19}F NMR (376.27 MHz, THF-d_8): δ = –131.7, –164.7, –168.3 ppm. HR-MS (ESI): $[M+H]^+$ calcd. (m/z) 1601.3905, found 1601.3891 (12%). UV-vis ($1.4 \cdot 10^{-5}$ M, THF): λ_{max} (ε in $M^{-1}cm^{-1}$) = 299 (43700), 323 (23800), 455 (29600) nm. IR spectrum (KBr pellet): ν = 2928 (w, C-H), 1642 (m), 1512 (s), 1462 (s), 1382 (m), 1092 (m), 973 (m) cm^{-1}.

Crystal data for $C_{66}H_{48}N_{12}B_2F_{30} \cdot 2(C_4H_8O)$: M_r = 1600.78, 0.37 × 0.268 × 0.257 mm^3, triclinic, space group $P\bar{1}$, a = 13.5320(10), b = 16.2347(12), c = 16.3380(11) Å, α = 68.479(2), β = 89.404(3), γ = 87.235(3)°, V = 3335.0(4) $Å^3$, Z = 2, d_{calc} = 1.594 Mg m^{-3}, Mo K_α radiation (graphite monochromated, λ = 0.71073 Å), T = 100 K, θ_{range} 2.54 to 27.39°, reflections measured: 129445, indep: 15394, R_{int} = 0.0579, final R indices [$I > 2\sigma(I)$]: R_1 = 0.0457, wR_2 = 0.1225.

Figure S40: ^{1}H NMR spectrum (600.13 MHz, THF-d_8) of **5**

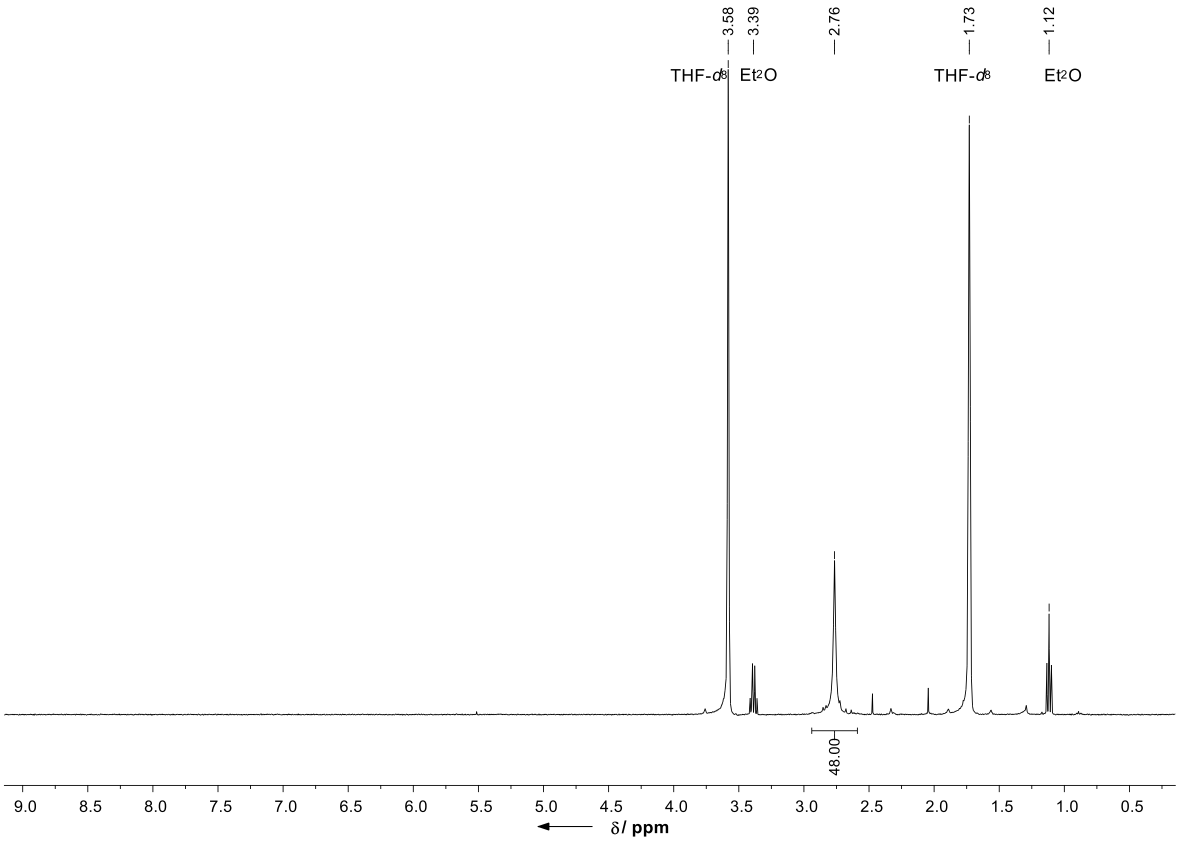

Figure S41: ^{13}C NMR spectrum (150.92 MHz, THF-d_8) of **5**

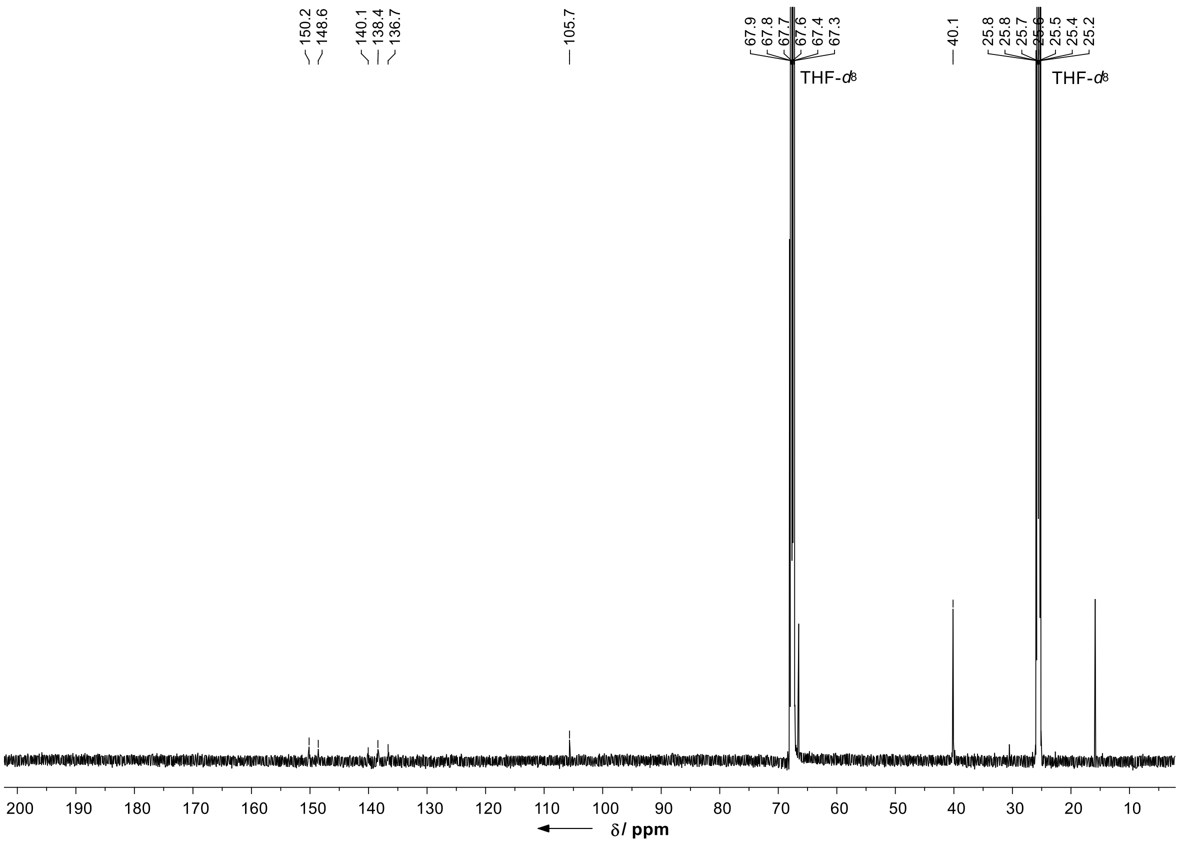

Figure S42: ^{11}B NMR spectrum (128.30 MHz, THF-d_8) of **5**

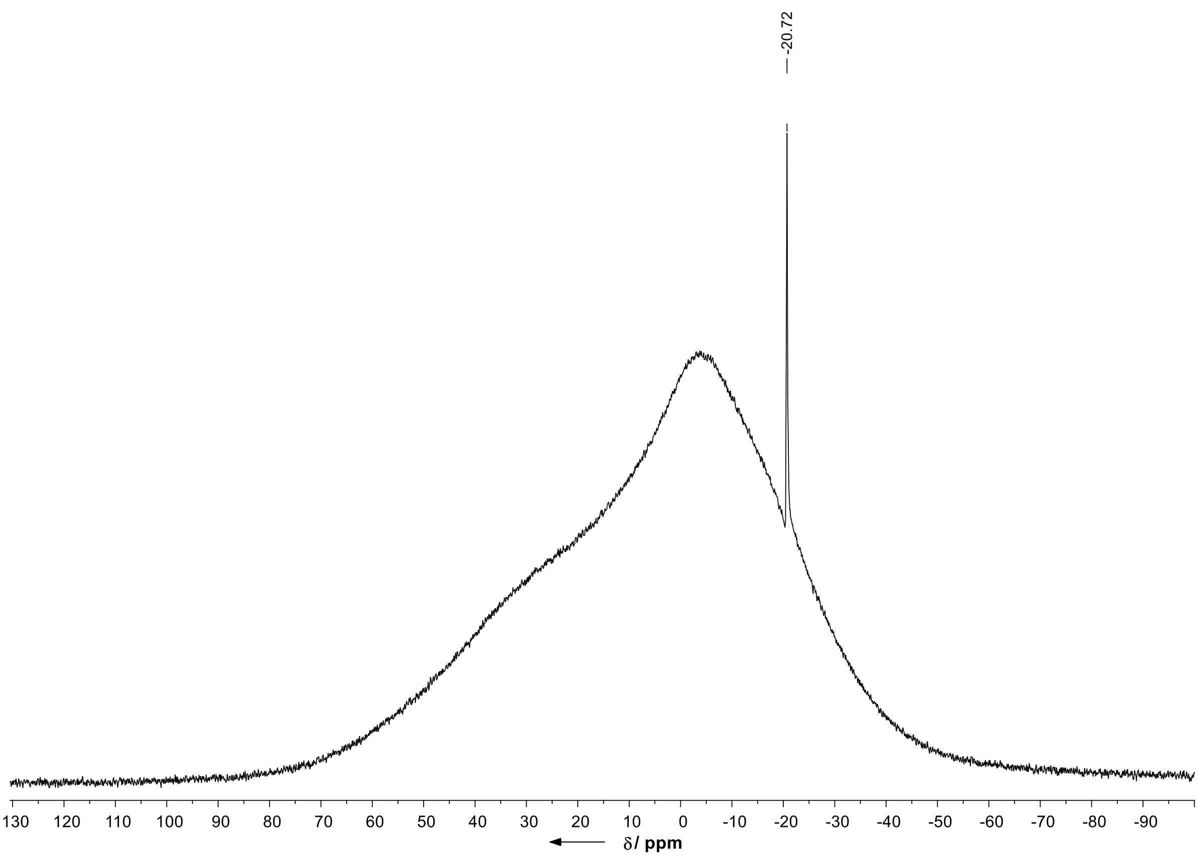

Figure S42: UV-vis spectrum (THF) of **5**

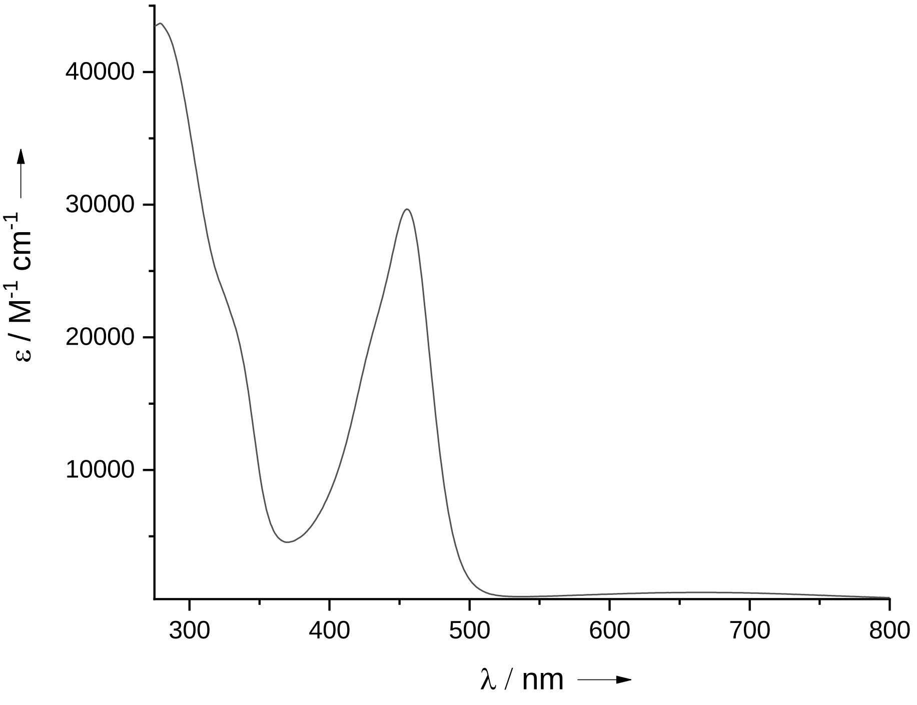

Figure S43: IR spectrum (KBr pellet) of **5**

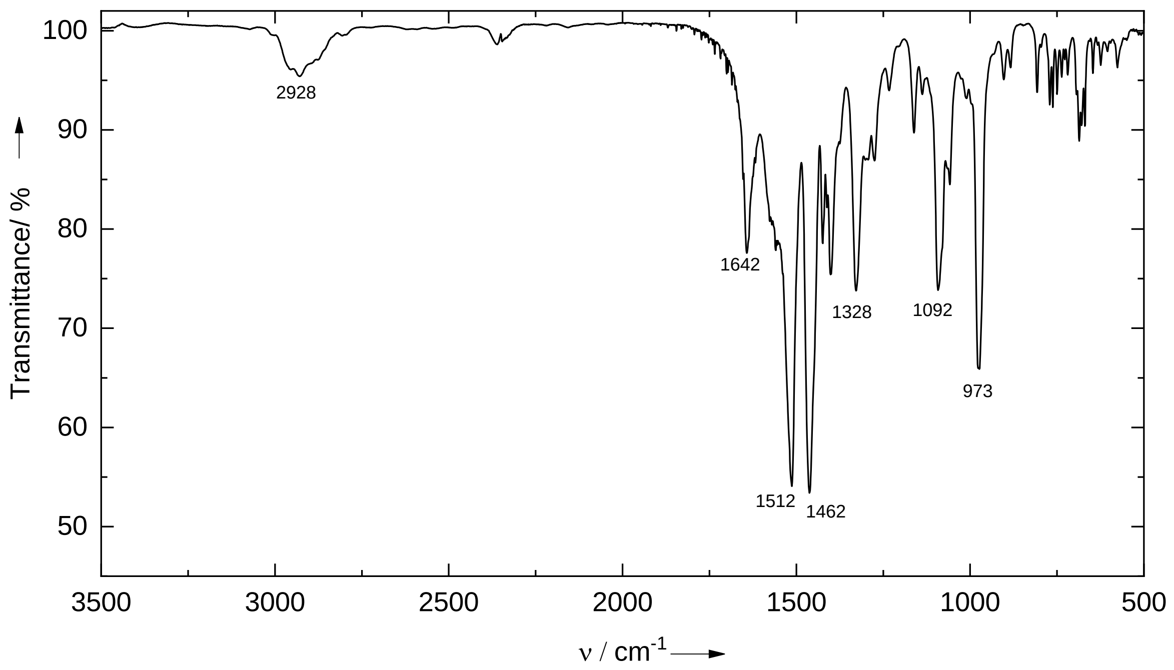

Figure S44: Cyclic voltammograms of **5**

Solution of **5** (1 mM) in DMF, Ag/AgCl reference electrode, with $N(nBu)_4PF_6$ (0.1 M) as supporting electrolyte, measured at a scan rate of 100 mV s^{-1}. Potentials referenced to the ferrocenium/ferrocene (Fc^+/Fc) redox couple

a) Voltammogram in the potential window from −2 V to 0 V.

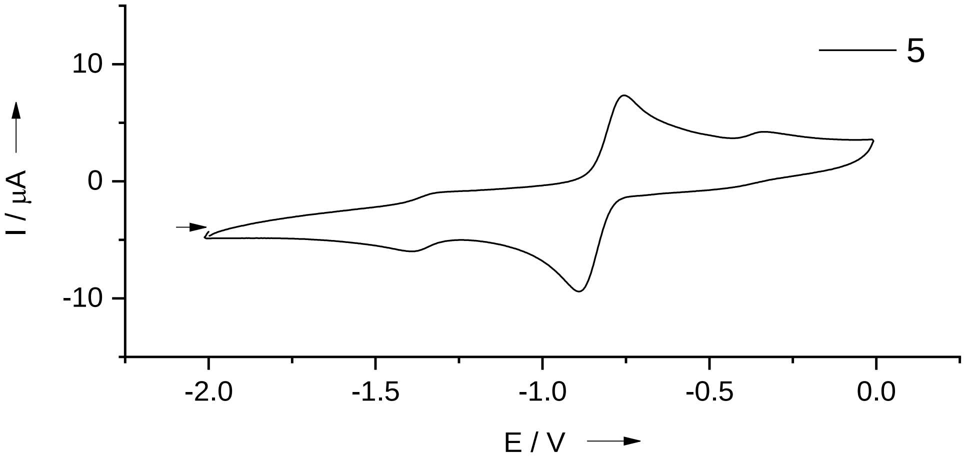

b) Voltammogram in the potential window from −1.5 V to +1.5 V.

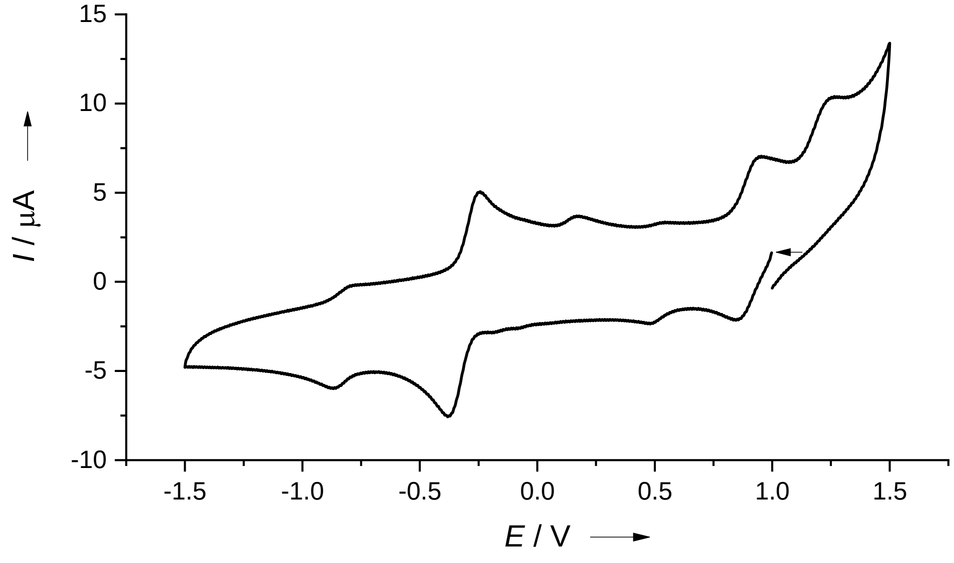

3) Results of the quantum chemical calculations

Quantum-chemical calculations are carried out for compounds **3**, **3**$^{2+}$, [**2a**(CuI)$_2$] and [**3**(CuI)$_2$]. These density functional calculations are performed with the program TURBOMOLE.[1-3] The B3LYP functional [4,5] is used in connection with the def2-SV(P) and def2-TZVP basis sets.[6] For the calculation of the two-electron integrals, the resolution-of-the-identity (RI) approximation [7] is used with the appropriate def2-SV(P) and def2-TZVP auxiliary basis set.[8] Structure optimizations are performed with the def2-SV(P) and def2-TZVP basis sets, the determination of the harmonic vibrational frequencies with the def2-SV(P) basis set only. The electronic excitation energies are obtained by time-dependent density functional calculations.[9,10]

[1] TURBOMOLE V7.2 2017, a development of University of Karlsruhe and Forschungszentrum Karlsruhe GmbH, 1989-2007, TURBOMOLE GmbH, since 2007; available from http://www.turbomole.com.

[2] R. Ahlrichs, M. Bär, M. Häser, H. Horn, C. Kölmel, *Chem. Phys. Lett.* **1989**, *162*, 165–169.

[3] O. Treutler, R. Ahlrichs, *J. Chem. Phys.* **1995**, *102*, 346–354.

[4] A. D. Becke, *J. Chem. Phys.* **1993**, *98*, 5648–5652.

[5] P. J. Stephens, F. J. Devlin, C. F. Chabalowski, M. J. Frisch, *J. Phys. Chem.* **1994**, *98*, 11623–11627.

[6] F. Weigend, R. Ahlrichs, *Phys. Chem. Chem. Phys.* **2005**, *7*, 3297–3305.

[7] K. Eichkorn, O. Treutler, H. Öhm, M. Häser, R. Ahlrichs, *Chem. Phys. Lett.* **1995**, *242*, 652–660.

[8] F. Weigend, *Phys. Chem. Chem. Phys.* **2006**, *8*, 1057–1065.

[9] R. Bauernschmitt, R. Ahlrichs, *Chem. Phys. Lett.* **1996**, *256*, 454–464.

[10] R. Bauernschmitt, M. Häser, O. Treuler, R. Ahlrichs, *Chem. Phys. Lett.* **1997**, *264*, 573–578.

Coordinates and electronic energy for the structure of **3** optimized with B3LYP/def2-SV(P)

Energy = -1827.519822105 Hartree

N	1.3857110	-0.1460542	2.4500340
N	3.0930246	0.3253025	3.9366471
N	2.1297625	2.1089591	2.7576741
N	-1.3857110	0.1460542	2.4500340
N	-3.0930246	-0.3253025	3.9366471
N	-2.1297625	-2.1089591	2.7576741
N	-1.3857110	0.1460542	-2.4500340
N	-2.1297625	-2.1089591	-2.7576741
N	-3.0930246	-0.3253025	-3.9366471
N	1.3857110	-0.1460542	-2.4500340
N	2.1297625	2.1089591	-2.7576741
N	3.0930246	0.3253025	-3.9366471
C	0.7066232	0.0116480	1.2409937
C	-0.7066232	-0.0116480	1.2409937
C	-1.4041468	-0.0178652	0.0000000
C	-0.7066232	-0.0116480	-1.2409937

C	0.7066232	0.0116480	-1.2409937
C	1.4041468	0.0178652	0.0000000
C	2.1507103	0.7360600	2.9967373
C	3.4148045	-1.0865288	4.0092235
H	2.6749479	-1.6591228	4.6082587
H	4.4096415	-1.2056866	4.4784589
H	3.4352115	-1.5144282	2.9964989
C	3.2649992	1.0513044	5.1830465
H	2.8559287	2.0673700	5.0905094
H	4.3355712	1.1239757	5.4567914
H	2.7358167	0.5439363	6.0192412
C	0.9062928	2.7776223	2.3581060
H	0.8477217	2.9424854	1.2623595
H	0.8575921	3.7678096	2.8547516
H	0.0318343	2.1847627	2.6689171
C	3.3529086	2.8627071	2.5601627
H	4.2242269	2.2266281	2.7734323
H	3.3937025	3.7562474	3.2170581
H	3.4327888	3.2114559	1.5087613
C	-2.1507103	-0.7360600	2.9967373
C	-3.2649992	-1.0513044	5.1830465
H	-2.8559287	-2.0673700	5.0905094
H	-4.3355712	-1.1239757	5.4567914
H	-2.7358167	-0.5439363	6.0192412
C	-3.4148045	1.0865288	4.0092235
H	-2.6749479	1.6591228	4.6082587
H	-4.4096415	1.2056866	4.4784589
H	-3.4352115	1.5144282	2.9964989
C	-0.9062928	-2.7776223	2.3581060
H	-0.8477217	-2.9424854	1.2623595
H	-0.8575921	-3.7678096	2.8547516
H	-0.0318343	-2.1847627	2.6689171
C	-3.3529086	-2.8627071	2.5601627
H	-4.2242269	-2.2266281	2.7734323
H	-3.3937025	-3.7562474	3.2170581
H	-3.4327888	-3.2114559	1.5087613
C	-2.1507103	-0.7360600	-2.9967373
C	-3.3529086	-2.8627071	-2.5601627
H	-3.4327888	-3.2114559	-1.5087613
H	-3.3937025	-3.7562474	-3.2170581
H	-4.2242269	-2.2266281	-2.7734323
C	-0.9062928	-2.7776223	-2.3581060
H	-0.0318343	-2.1847627	-2.6689171
H	-0.8575921	-3.7678096	-2.8547516
H	-0.8477217	-2.9424854	-1.2623595
C	-3.2649992	-1.0513044	-5.1830465
H	-2.7358167	-0.5439363	-6.0192412
H	-4.3355712	-1.1239757	-5.4567914
H	-2.8559287	-2.0673700	-5.0905094
C	-3.4148045	1.0865288	-4.0092235
H	-3.4352115	1.5144282	-2.9964989
H	-4.4096415	1.2056866	-4.4784589
H	-2.6749479	1.6591228	-4.6082587
C	2.1507103	0.7360600	-2.9967373
C	0.9062928	2.7776223	-2.3581060
H	0.0318343	2.1847627	-2.6689171

H	0.8575921	3.7678096	-2.8547516
H	0.8477217	2.9424854	-1.2623595
C	3.3529086	2.8627071	-2.5601627
H	4.2242269	2.2266281	-2.7734323
H	3.4327888	3.2114559	-1.5087613
H	3.3937025	3.7562474	-3.2170581
C	3.4148045	-1.0865288	-4.0092235
H	3.4352115	-1.5144282	-2.9964989
H	4.4096415	-1.2056866	-4.4784589
H	2.6749479	-1.6591228	-4.6082587
C	3.2649992	1.0513044	-5.1830465
H	2.7358167	0.5439363	-6.0192412
H	4.3355712	1.1239757	-5.4567914
H	2.8559287	2.0673700	-5.0905094
C	-2.8339067	0.0313441	0.0000000
C	-4.0504498	0.0856063	0.0000000
H	-5.1232320	0.1362330	0.0000000
C	2.8339067	-0.0313441	0.0000000
C	4.0504498	-0.0856063	0.0000000
H	5.1232320	-0.1362330	0.0000000

<u>Coordinates and electronic energy for the structure of **3** optimized with B3LYP/def2-TZVP</u>

Energy = -1829.629738329 Hartree

N	1.3847178	-0.1333310	2.4420895
N	3.0882692	0.3245295	3.9246719
N	2.1253097	2.1130927	2.7729752
N	-1.3847178	0.1333310	2.4420895
N	-3.0882692	-0.3245295	3.9246719
N	-2.1253097	-2.1130927	2.7729752
N	-1.3847178	0.1333310	-2.4420895
N	-2.1253097	-2.1130927	-2.7729752
N	-3.0882692	-0.3245295	-3.9246719
N	1.3847178	-0.1333310	-2.4420895
N	2.1253097	2.1130927	-2.7729752
N	3.0882692	0.3245295	-3.9246719
C	0.7020370	0.0220853	1.2348727
C	-0.7020370	-0.0220853	1.2348727
C	-1.3945604	-0.0408014	0.0000000
C	-0.7020370	-0.0220853	-1.2348727
C	0.7020370	0.0220853	-1.2348727
C	1.3945604	0.0408014	0.0000000
C	2.1454823	0.7435285	2.9908808
C	3.3790615	-1.0959245	3.9968320
H	2.6166389	-1.6496750	4.5599299
H	4.3428147	-1.2302776	4.4944583
H	3.4285534	-1.5121507	2.9945039
C	3.1986690	1.0120525	5.2025064
H	2.8659505	2.0417823	5.1113518
H	4.2360886	1.0081636	5.5475230
H	2.5846271	0.5211408	5.9707087
C	0.9149702	2.7864895	2.3429906

H	0.8925541	2.9622951	1.2612011
H	0.8578204	3.7590615	2.8434437
H	0.0404301	2.2020130	2.6204120
C	3.3471701	2.8843335	2.6402158
H	4.2084377	2.2624326	2.8674631
H	3.3467961	3.7497139	3.3134923
H	3.4549206	3.2570750	1.6144302
C	-2.1454823	-0.7435285	2.9908808
C	-3.1986690	-1.0120525	5.2025064
H	-2.8659505	-2.0417823	5.1113518
H	-4.2360886	-1.0081636	5.5475230
H	-2.5846271	-0.5211408	5.9707087
C	-3.3790615	1.0959245	3.9968320
H	-2.6166389	1.6496750	4.5599299
H	-4.3428147	1.2302776	4.4944583
H	-3.4285534	1.5121507	2.9945039
C	-0.9149702	-2.7864895	2.3429906
H	-0.8925541	-2.9622951	1.2612011
H	-0.8578204	-3.7590615	2.8434437
H	-0.0404301	-2.2020130	2.6204120
C	-3.3471701	-2.8843335	2.6402158
H	-4.2084377	-2.2624326	2.8674631
H	-3.3467961	-3.7497139	3.3134923
H	-3.4549206	-3.2570750	1.6144302
C	-2.1454823	-0.7435285	-2.9908808
C	-3.3471701	-2.8843335	-2.6402158
H	-3.4549206	-3.2570750	-1.6144302
H	-3.3467961	-3.7497139	-3.3134923
H	-4.2084377	-2.2624326	-2.8674631
C	-0.9149702	-2.7864895	-2.3429906
H	-0.0404301	-2.2020130	-2.6204120
H	-0.8578204	-3.7590615	-2.8434437
H	-0.8925541	-2.9622951	-1.2612011
C	-3.1986690	-1.0120525	-5.2025064
H	-2.5846271	-0.5211408	-5.9707087
H	-4.2360886	-1.0081636	-5.5475230
H	-2.8659505	-2.0417823	-5.1113518
C	-3.3790615	1.0959245	-3.9968320
H	-3.4285534	1.5121507	-2.9945039
H	-4.3428147	1.2302776	-4.4944583
H	-2.6166389	1.6496750	-4.5599299
C	2.1454823	0.7435285	-2.9908808
C	0.9149702	2.7864895	-2.3429906
H	0.0404301	2.2020130	-2.6204120
H	0.8578204	3.7590615	-2.8434437
H	0.8925541	2.9622951	-1.2612011
C	3.3471701	2.8843335	-2.6402158
H	4.2084377	2.2624326	-2.8674631
H	3.4549206	3.2570750	-1.6144302
H	3.3467961	3.7497139	-3.3134923
C	3.3790615	-1.0959245	-3.9968320
H	3.4285534	-1.5121507	-2.9945039
H	4.3428147	-1.2302776	-4.4944583
H	2.6166389	-1.6496750	-4.5599299
C	3.1986690	1.0120525	-5.2025064
H	2.5846271	0.5211408	-5.9707087

H	4.2360886	1.0081636	-5.5475230
H	2.8659505	2.0417823	-5.1113518
C	-2.8180918	-0.0122098	0.0000000
C	-4.0232208	0.0280256	0.0000000
H	-5.0843300	0.0644506	0.0000000
C	2.8180918	0.0122098	0.0000000
C	4.0232208	-0.0280256	0.0000000
H	5.0843300	-0.0644506	0.0000000

Electronic transitions for **3** found in the TD-DFT calculations

λ/nm	oscillator strength	leading contribution		
402.7	0.155	$36b_g \rightarrow 43b_u$	HOMO → LUMO	96.4%
314.6	0.022	$43a_g \rightarrow 43b_u$	HOMO-1 → LUMO	63.3%
296.9	0.359	$36b_g \rightarrow 37a_u$	HOMO → LUMO+1	59.9%
268.2	0.096	$36b_g \rightarrow 38a_u$	HOMO → LUMO+5	94.6%
260.3	0.233	$43a_g \rightarrow 37a_u$	HOMO-1 → LUMO+1	83.4%
240.1	0.131	$35b_g \rightarrow 37a_u$	HOMO-2 → LUMO+1	75.4%

Figure S45. Simulation of the electronic excitation spectrum of **3** on the basis of the TD-DFT calculations

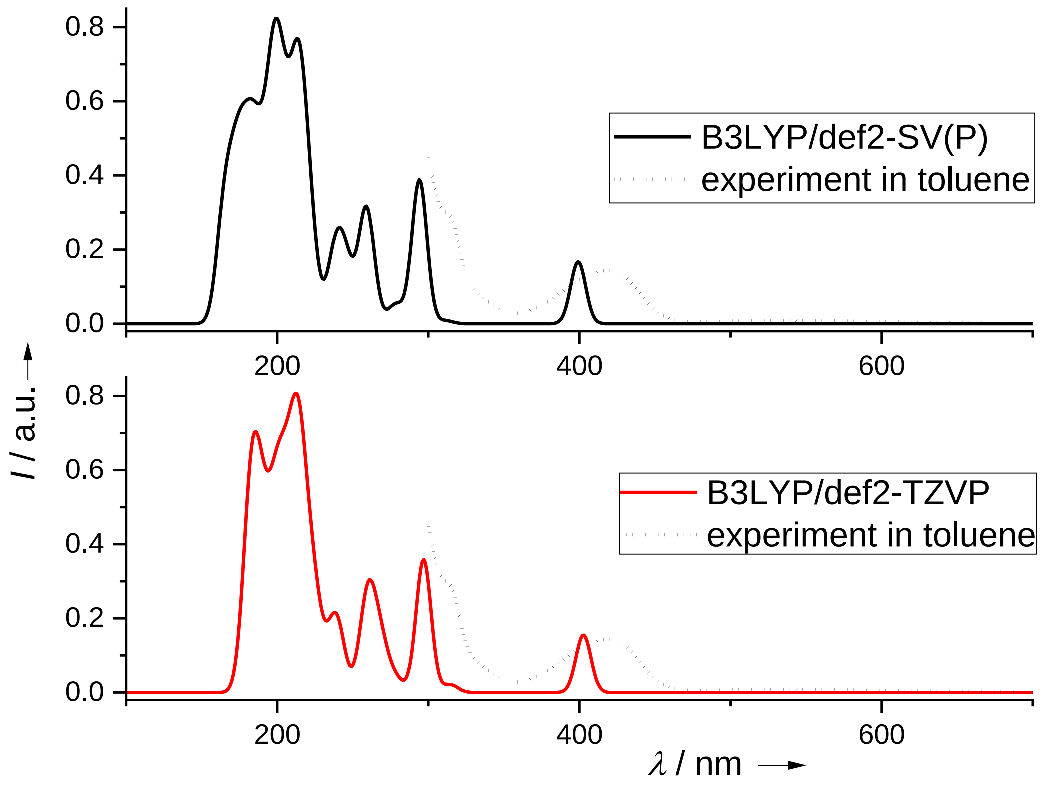

Figure S46. Isodensity plots for the relevant orbitals of **3.** Contour values for the isodensity plots are ±0.02 Bohr$^{(-3/2)}$.

λ = 402.7 nm (96.4% HOMO → LUMO)

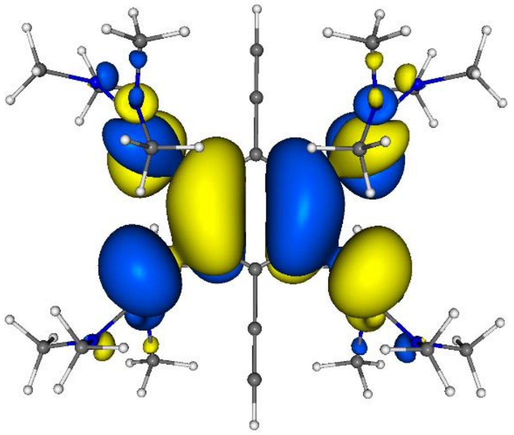

HOMO

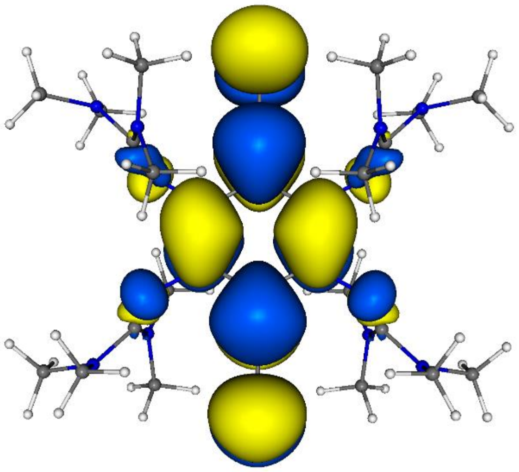

LUMO

λ = 314.6 nm (63.3% HOMO-1 → LUMO)

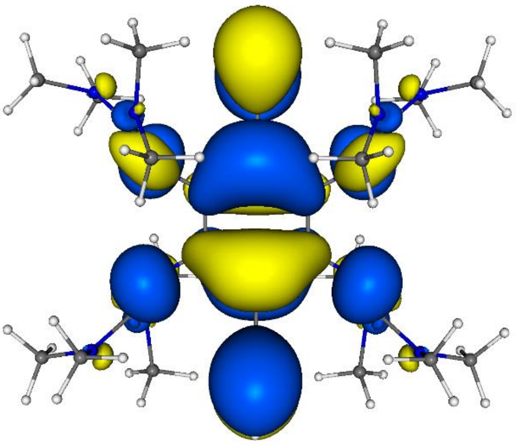

HOMO-1

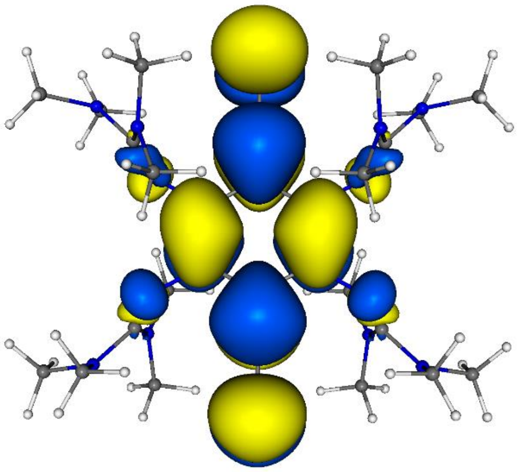

LUMO

λ = 296.9 nm (59.9% HOMO → LUMO+1)

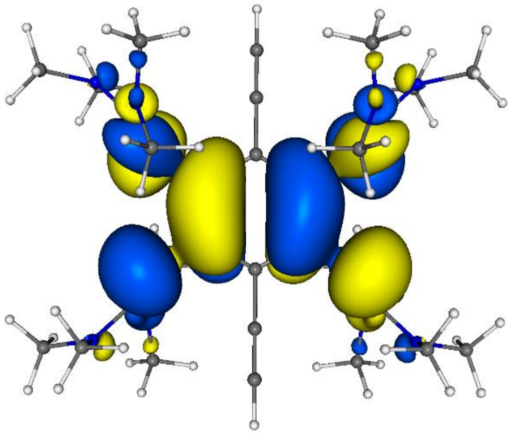

HOMO

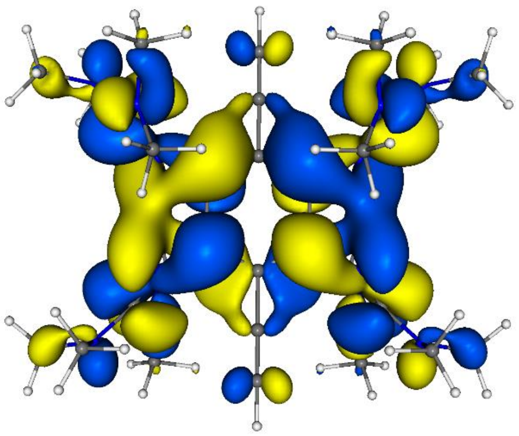

LUMO+1

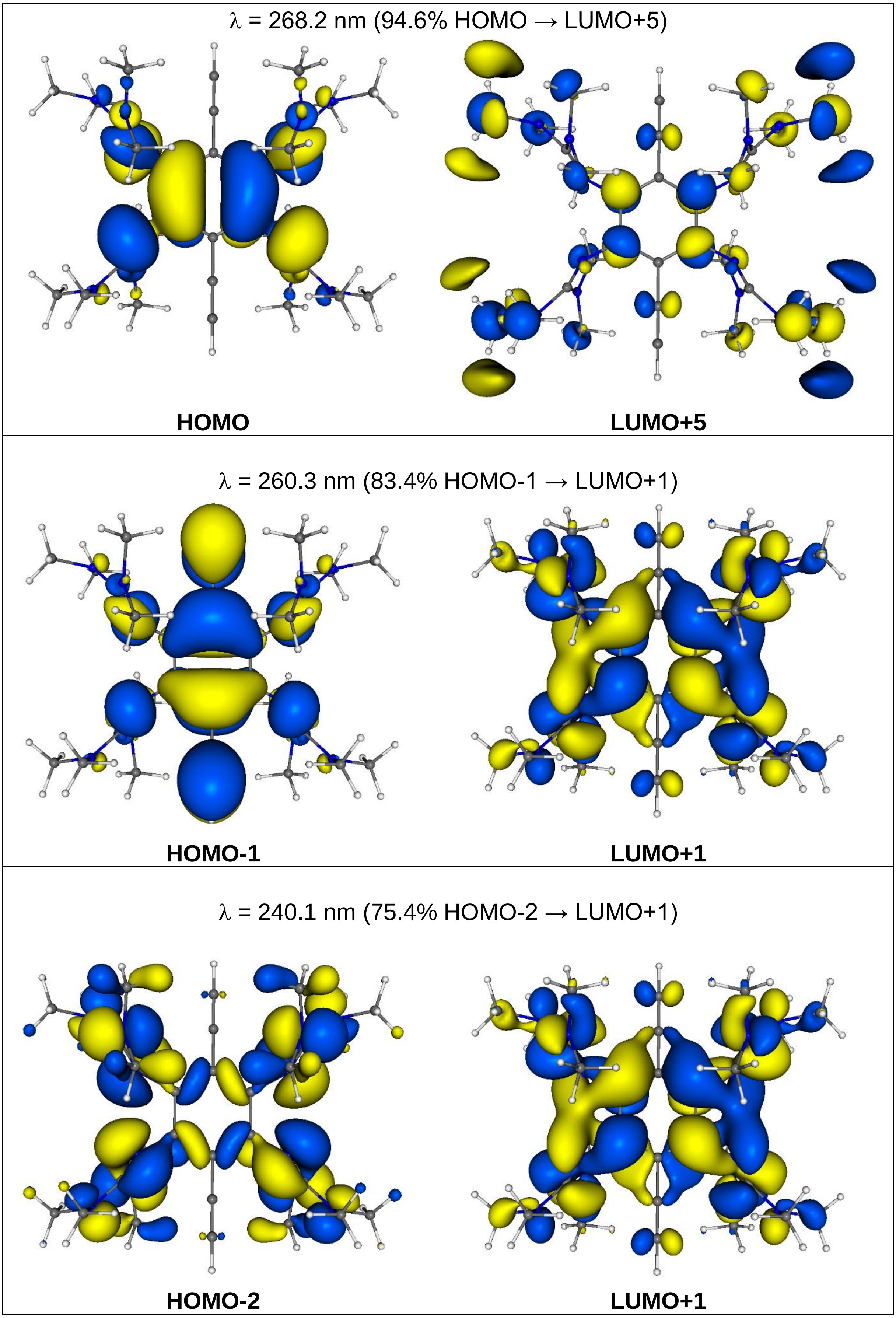
λ = 268.2 nm (94.6% HOMO → LUMO+5)
HOMO
LUMO+5
λ = 260.3 nm (83.4% HOMO-1 → LUMO+1)
HOMO-1
LUMO+1
λ = 240.1 nm (75.4% HOMO-2 → LUMO+1)
HOMO-2
LUMO+1

Calculations for **3**$^{2+}$

Coordinates and electronic energy for the structure of **3**$^{2+}$ optimized with B3LYP/def2-TZVP

Energy = -1829.181551 Hartree

N	-0.6179374	-1.3701165	-2.3448261
N	1.7271525	-1.1939581	4.1088118
N	-0.0137782	-3.0084769	-3.8622523
N	3.5433617	-0.2317812	2.9877073
N	-1.1445747	-3.6276684	-1.9194110
N	1.7563744	-1.0678559	1.7866834
C	-0.5543598	-2.6695460	-2.6740836
C	-0.2690021	-0.7915563	-1.2049877
C	0.6843357	-1.2416836	-0.2874337
C	1.4621392	-2.4016301	-0.5440661
C	2.3029702	-0.7663626	2.9680073
C	0.9441619	-0.5335877	0.9418609
C	2.1802878	-3.3468824	-0.7482232
H	2.8176729	-4.1812010	-0.9204674
C	2.5059655	-1.7630110	5.2169101
H	3.5531868	-1.8509499	4.9465781
H	2.1265346	-2.7645780	5.4258927
H	2.4138702	-1.1630018	6.1236542
C	-2.0852990	-3.2938242	-0.8585255
H	-1.5884127	-3.2243635	0.1132266
H	-2.8408758	-4.0782644	-0.8037133
H	-2.5777535	-2.3505237	-1.0745846
C	-0.7016471	-5.0239661	-1.9130122
H	-1.4584185	-5.6888807	-2.3338234
H	-0.5163073	-5.3220342	-0.8801802
H	0.2251283	-5.1325194	-2.4659125
C	-0.5389319	-4.0719715	-4.7198175
H	0.1645824	-4.9003610	-4.8216767
H	-0.7187368	-3.6563793	-5.7135908
H	-1.4812966	-4.4469152	-4.3327762
C	0.3150969	-1.5688493	4.1069852
H	-0.2602439	-0.8734025	3.5054381
H	-0.0535853	-1.5236090	5.1308046
H	0.1764354	-2.5867254	3.7310295
C	4.3625298	-0.1860264	1.7788817
H	4.0694221	-0.9799771	1.0979960
H	5.4055750	-0.3272533	2.0632676
H	4.2761693	0.7766267	1.2668672
C	4.0549726	0.5423777	4.1173348
H	3.2689345	0.7217716	4.8441036
H	4.4065027	1.5068609	3.7448982
H	4.8931347	0.0420369	4.6063382

C	0.9565871	-2.1287432	-4.5050309
H	0.4736274	-1.4608920	-5.2239260
H	1.6831660	-2.7421639	-5.0391706
H	1.4835417	-1.5400477	-3.7611770
N	0.6179374	1.3701165	2.3448261
N	-1.7271525	1.1939581	-4.1088118
N	0.0137782	3.0084769	3.8622523
N	-3.5433617	0.2317812	-2.9877073
N	1.1445747	3.6276684	1.9194110
N	-1.7563744	1.0678559	-1.7866834
C	0.5543598	2.6695460	2.6740836
C	0.2690021	0.7915563	1.2049877
C	-0.6843357	1.2416836	0.2874337
C	-1.4621392	2.4016301	0.5440661
C	-2.3029702	0.7663626	-2.9680073
C	-0.9441619	0.5335877	-0.9418609
C	-2.1802878	3.3468824	0.7482232
H	-2.8176729	4.1812010	0.9204674
C	-2.5059655	1.7630110	-5.2169101
H	-3.5531868	1.8509499	-4.9465781
H	-2.1265346	2.7645780	-5.4258927
H	-2.4138702	1.1630018	-6.1236542
C	2.0852990	3.2938242	0.8585255
H	1.5884127	3.2243635	-0.1132266
H	2.8408758	4.0782644	0.8037133
H	2.5777535	2.3505237	1.0745846
C	0.7016471	5.0239661	1.9130122
H	1.4584185	5.6888807	2.3338234
H	0.5163073	5.3220342	0.8801802
H	-0.2251283	5.1325194	2.4659125
C	0.5389319	4.0719715	4.7198175
H	-0.1645824	4.9003610	4.8216767
H	0.7187368	3.6563793	5.7135908
H	1.4812966	4.4469152	4.3327762
C	-0.3150969	1.5688493	-4.1069852
H	0.2602439	0.8734025	-3.5054381
H	0.0535853	1.5236090	-5.1308046
H	-0.1764354	2.5867254	-3.7310295
C	-4.3625298	0.1860264	-1.7788817
H	-4.0694221	0.9799771	-1.0979960
H	-5.4055750	0.3272533	-2.0632676
H	-4.2761693	-0.7766267	-1.2668672
C	-4.0549726	-0.5423777	-4.1173348
H	-3.2689345	-0.7217716	-4.8441036
H	-4.4065027	-1.5068609	-3.7448982
H	-4.8931347	-0.0420369	-4.6063382
C	-0.9565871	2.1287432	4.5050309
H	-0.4736274	1.4608920	5.2239260
H	-1.6831660	2.7421639	5.0391706
H	-1.4835417	1.5400477	3.7611770

Electronic transitions of **3**$^{2+}$ found in the TD-DFT calculations (B3LYP/def2-TZVP)
Only electronic ttransitions with an intensity of more than 1.10^{-2} km/mol are listed

λ/nm	oscillator strength	leading contribution		
439.2	0.308	$78a_u \rightarrow 79a_g$	HOMO-1 → LUMO	77.5%
414.6	0.066	$77a_u \rightarrow 79a_g$	HOMO-2 → LUMO	71.8%
320.8	0.108	$78a_g \rightarrow 79a_u$	HOMO-4 → LUMO	83.6%

Figure S47. Comparison between experimental UV-vis spectrum of **3**$^{2+}$$(PF_6^-)_2$ and simulated electronic excitation spectrum of **3**$^{2+}$

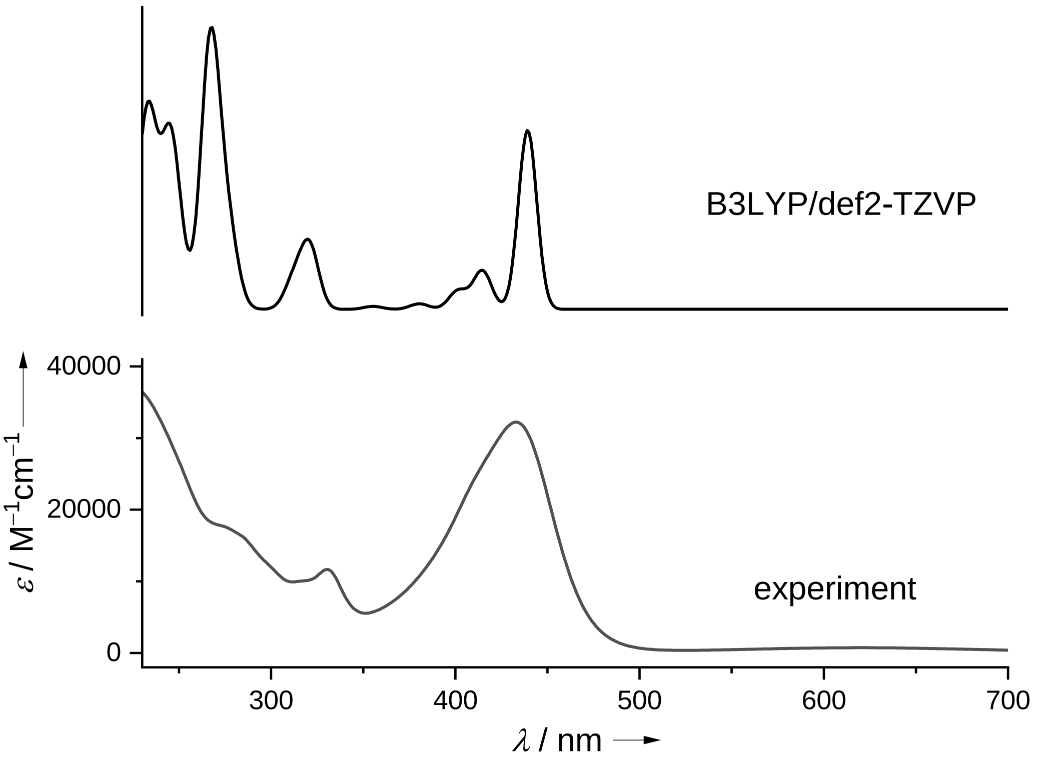

Figure S48. Isodensity plots for the relevant orbitals of **3**$^{2+}$. Contour values for the isodensity plots are ±0.02 Bohr$^{(-3/2)}$.

λ = 439.2 nm (77.5% HOMO-1 → LUMO)

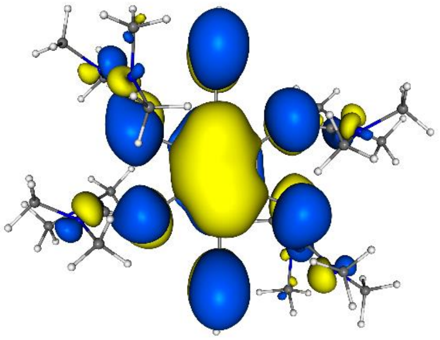

HOMO-1

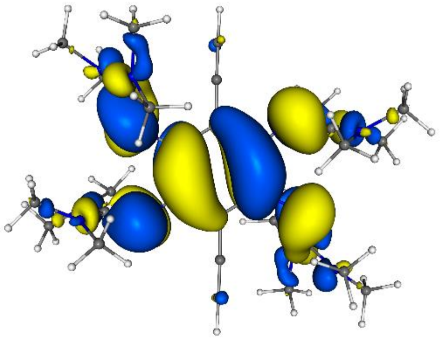

LUMO

λ = 414.6 nm (71.8% HOMO-2 → LUMO)

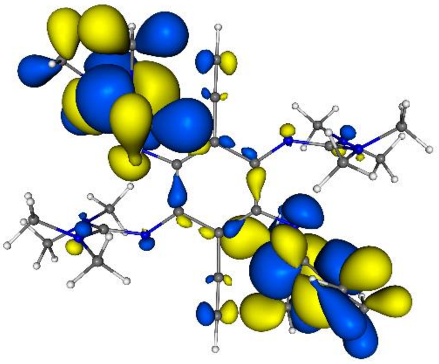

HOMO-2

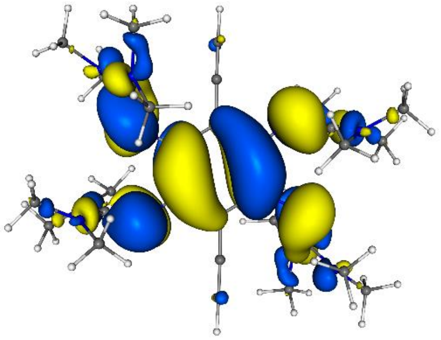

LUMO

λ = 296.9 nm (59.9% HOMO → LUMO+1)

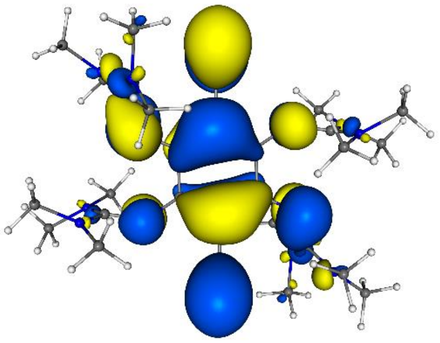

HOMO

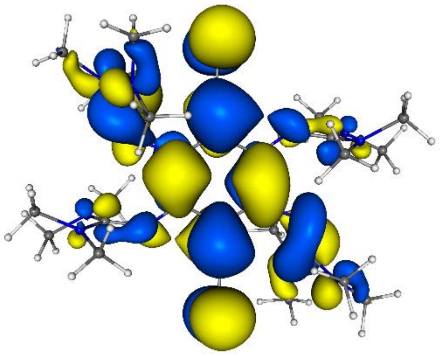

LUMO+1

Calculations for the complex [**3**($CuI)_2$]

Coordinates and electronic energy for the structure of [**3**($CuI)_2$] optimized with B3LYP/def2-TZVP

Energy = -5706.190762 Hartree

I	0.0000000	6.4759342	1.3618923
Cu	0.0000000	4.0023375	1.0826340
N	-1.3405159	2.4672183	0.6554755
N	-2.9351779	2.5207999	-1.1117270
N	-3.3118854	3.6561568	0.8818308
N	1.3405159	2.4672183	0.6554755
N	2.9351779	2.5207999	-1.1117270
N	3.3118854	3.6561568	0.8818308
C	-0.7080381	1.2384467	0.4583099
C	0.7080381	1.2384467	0.4583099
C	-1.3983189	0.0000000	0.5170728
C	-2.4963045	2.8429431	0.1505363
C	-4.3375267	2.3222184	-1.4332466
H	-4.9393619	2.3900096	-0.5323353
H	-4.4800670	1.3270727	-1.8652905
H	-4.6951126	3.0618921	-2.1575000
C	-2.0012106	2.2135747	-2.1735758
H	-1.0378431	2.6623690	-1.9522001
H	-2.3730262	2.6382763	-3.1099121
H	-1.8619503	1.1365614	-2.3146702
C	-3.9625288	4.8237365	0.2945237
H	-3.8215524	4.8370336	-0.7820586
H	-3.5142554	5.7329094	0.7042123
H	-5.0340509	4.8278079	0.5143771
C	-3.1647344	3.7168975	2.3301388
H	-2.8434433	2.7509364	2.7086293
H	-4.1360751	3.9677195	2.7633773
H	-2.4383468	4.4785680	2.6291169
C	2.4963045	2.8429431	0.1505363
C	2.0012106	2.2135747	-2.1735758
H	1.8619503	1.1365614	-2.3146702
H	2.3730262	2.6382763	-3.1099121
H	1.0378431	2.6623690	-1.9522001
C	4.3375267	2.3222184	-1.4332466
H	4.6951126	3.0618921	-2.1575000
H	4.4800670	1.3270727	-1.8652905
H	4.9393619	2.3900096	-0.5323353
C	3.1647344	3.7168975	2.3301388
H	2.4383468	4.4785680	2.6291169
H	4.1360751	3.9677195	2.7633773
H	2.8434433	2.7509364	2.7086293
C	3.9625288	4.8237365	0.2945237
H	3.8215524	4.8370336	-0.7820586

H	5.0340509	4.8278079	0.5143771
H	3.5142554	5.7329094	0.7042123
C	-2.7832014	0.0000000	0.8450541
C	-3.9303346	0.0000000	1.2155183
H	-4.9395199	0.0000000	1.5466161
N	1.3405159	-2.4672183	0.6554755
N	2.9351779	-2.5207999	-1.1117270
N	3.3118854	-3.6561568	0.8818308
N	-1.3405159	-2.4672183	0.6554755
N	-2.9351779	-2.5207999	-1.1117270
N	-3.3118854	-3.6561568	0.8818308
C	0.7080381	-1.2384467	0.4583099
C	-0.7080381	-1.2384467	0.4583099
C	1.3983189	0.0000000	0.5170728
C	2.4963045	-2.8429431	0.1505363
C	4.3375267	-2.3222184	-1.4332466
H	4.9393619	-2.3900096	-0.5323353
H	4.4800670	-1.3270727	-1.8652905
H	4.6951126	-3.0618921	-2.1575000
C	2.0012106	-2.2135747	-2.1735758
H	1.0378431	-2.6623690	-1.9522001
H	2.3730262	-2.6382763	-3.1099121
H	1.8619503	-1.1365614	-2.3146702
C	3.9625288	-4.8237365	0.2945237
H	3.8215524	-4.8370336	-0.7820586
H	3.5142554	-5.7329094	0.7042123
H	5.0340509	-4.8278079	0.5143771
C	3.1647344	-3.7168975	2.3301388
H	2.8434433	-2.7509364	2.7086293
H	4.1360751	-3.9677195	2.7633773
H	2.4383468	-4.4785680	2.6291169
C	-2.4963045	-2.8429431	0.1505363
C	-2.0012106	-2.2135747	-2.1735758
H	-1.8619503	-1.1365614	-2.3146702
H	-2.3730262	-2.6382763	-3.1099121
H	-1.0378431	-2.6623690	-1.9522001
C	-4.3375267	-2.3222184	-1.4332466
H	-4.6951126	-3.0618921	-2.1575000
H	-4.4800670	-1.3270727	-1.8652905
H	-4.9393619	-2.3900096	-0.5323353
C	-3.1647344	-3.7168975	2.3301388
H	-2.4383468	-4.4785680	2.6291169
H	-4.1360751	-3.9677195	2.7633773
H	-2.8434433	-2.7509364	2.7086293
C	-3.9625288	-4.8237365	0.2945237
H	-3.8215524	-4.8370336	-0.7820586
H	-5.0340509	-4.8278079	0.5143771
H	-3.5142554	-5.7329094	0.7042123
C	2.7832014	0.0000000	0.8450541
C	3.9303346	0.0000000	1.2155183
H	4.9395199	0.0000000	1.5466161

I 0.0000000 -6.4759342 1.3618923
Cu 0.0000000 -4.0023375 1.0826340

Electronic transitions of [**3**$(CuI)_2$] found in the TD-DFT calculations (B3LYP/def2-TZVP)
Only electronic ttransitions with an intensity of more than 1.10^{-2} km/mol are listed

λ/nm	oscillator strength	leading contribution		
497.7	0.009	$50b_1 \rightarrow 63a_1$	HOMO-1 → LUMO	98.8%
470.6	0.161	$56b_2 \rightarrow 63a_1$	HOMO-2 → LUMO	97.8%
393.5	0.031	$55b_2 \rightarrow 63a_1$	HOMO-4 → LUMO	97.5%
354.8	0.068	$50b_1 \rightarrow 44a_2$	HOMO-1 → LUMO+1	89.6%
339.2	0.047	$56b_2 \rightarrow 44a_2$	HOMO-2 → LUMO+1	43.0%
321.3	0.219	$56b_2 \rightarrow 44a_2$	HOMO-2 → LUMO+1	28.7%

Figure S49. Comparison between experimental and simulated electronic excitation spectrum of [**3**$(CuI)_2$]

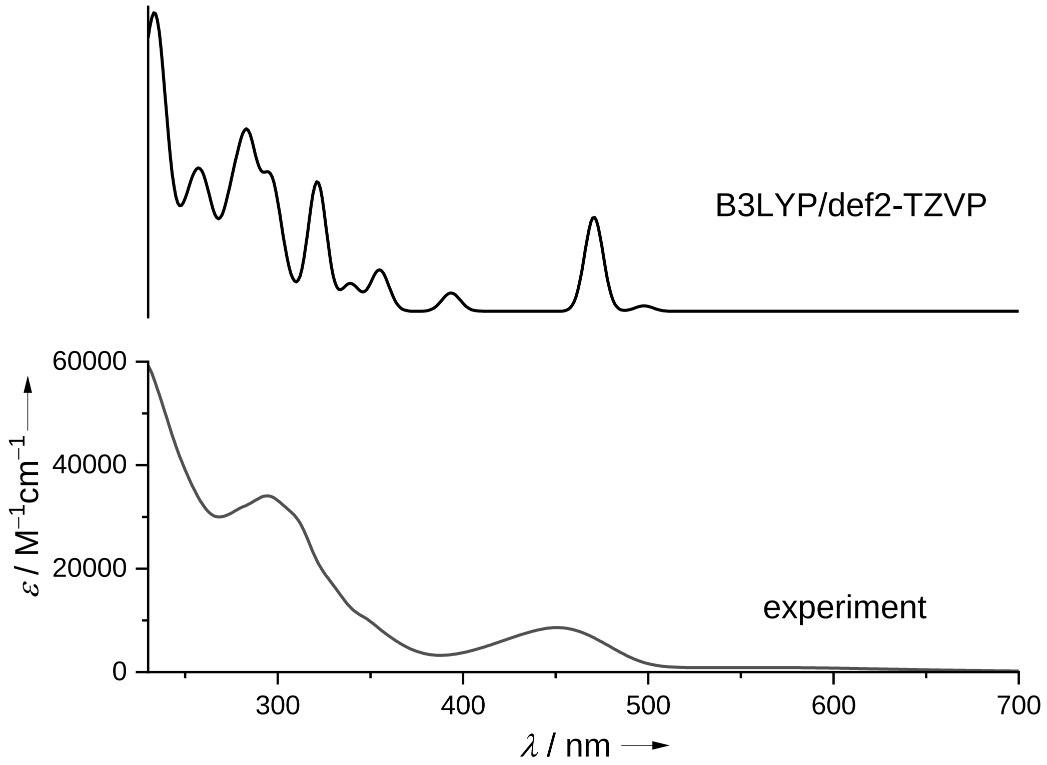

Figure S50. Isodensity plots for the relavant orbitals of [**3**$(CuI)_2$]. Contour values for the isodensity plots are ±0.02 $Bohr^{(-3/2)}$.

λ = 497.7 nm (98.8% HOMO-1 → LUMO)

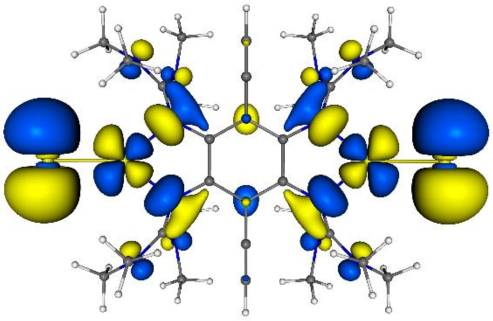

HOMO-1

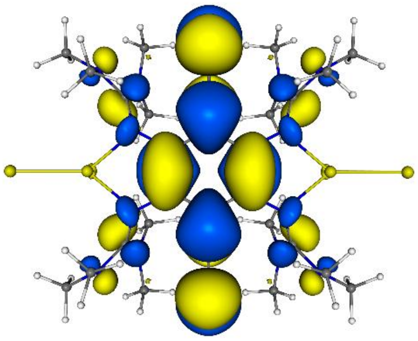

LUMO

λ = 470.6 nm (97.8% HOMO-2 → LUMO)

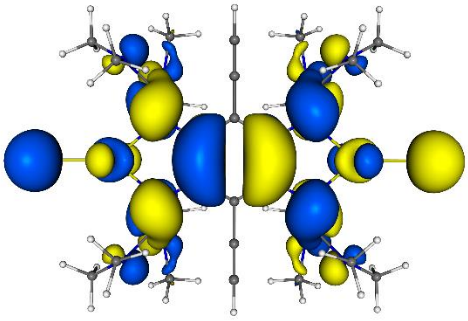

HOMO-2

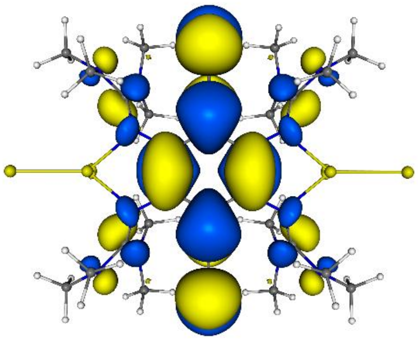

LUMO

λ = 393.5 nm (97.5% HOMO-4 → LUMO)

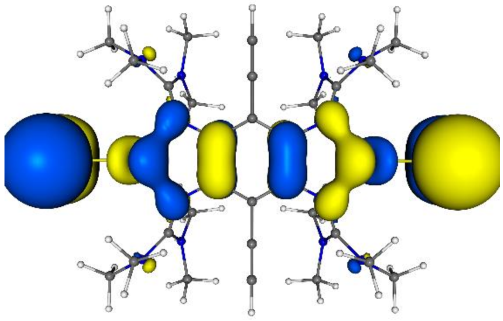

HOMO-4

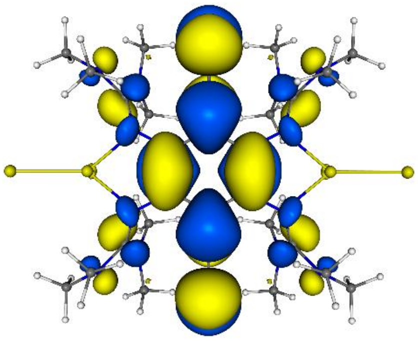

LUMO+1

λ = 354.8 nm (89.6% HOMO-1 → LUMO+1)

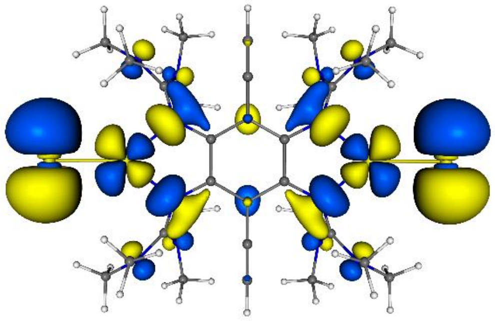

HOMO-1

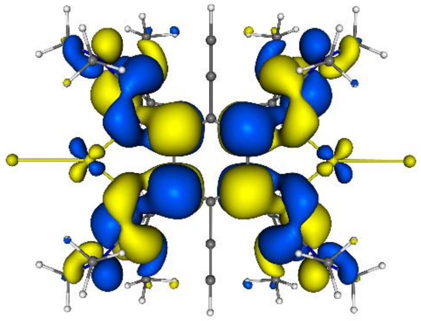

LUMO+1

λ = 339.2 nm (43.0% HOMO-2 → LUMO+1)

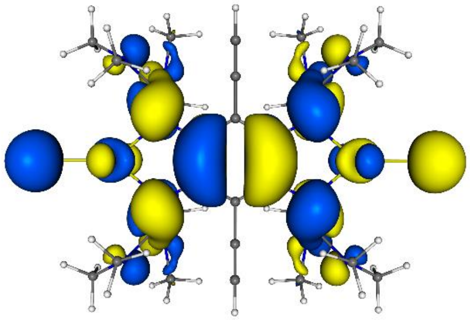

HOMO-2

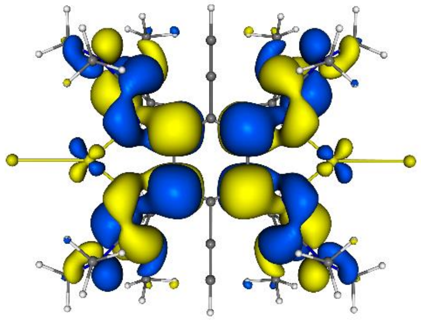

LUMO+1

λ = 321.3 nm (28.7% HOMO-2 → LUMO+1)

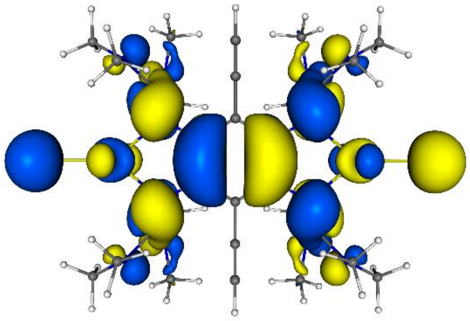

HOMO-2

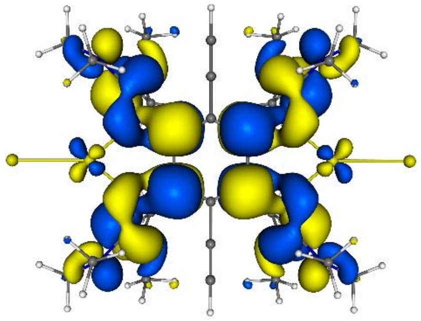

LUMO+1

Calculations for the complex [**2a**$(CuI)_2$]

Coordinates and electronic energy for the structure of [**2a**$(CuI)_2$] optimized with B3LYP/def2-TZVP

Energy = -6994.963915 Hartree

I	4.4413335	3.6752108	-3.1445463
Cu	2.7286013	2.3031139	-1.9802671
Si	0.4890142	3.3480733	4.6885857
N	1.2320707	0.9053413	-2.3683372
N	1.1566656	1.2388534	-4.6576832
N	-0.6087975	2.0394132	-3.3554300
N	1.9951171	1.9956710	-0.0278371
N	3.5929676	3.2702468	1.0550311
N	3.7144402	0.9489979	1.2411383
C	0.5782987	0.4668136	-1.2066185
C	1.0341159	0.9769897	0.0256278
C	0.5905368	1.3714790	-3.4248279
C	1.1397386	2.3294393	-5.6303398
H	0.5026251	3.1387912	-5.2891832
H	0.7834860	1.9782773	-6.6023935
H	2.1512738	2.7275496	-5.7442825
C	2.2649266	0.3104917	-4.8358055
H	3.2186336	0.7764109	-4.5689929
H	2.3000253	0.0142639	-5.8859490
H	2.1138105	-0.5661815	-4.2145364
C	-1.6226906	1.9705676	-4.3901096
H	-1.7694735	2.9366637	-4.8860078
H	-2.5787649	1.6746337	-3.9473376
H	-1.3489980	1.2309873	-5.1356479
C	-0.9273872	2.8915966	-2.2224394
H	-1.6898295	2.4528665	-1.5736503
H	-1.3049084	3.8492445	-2.5941909
H	-0.0361781	3.0812744	-1.6301756
C	3.0593126	2.0521378	0.7530405
C	2.8108457	4.4736397	0.8082603
H	2.9233109	4.8138677	-0.2258895
H	3.1675101	5.2605657	1.4760989
H	1.7617819	4.2796311	1.0051909
C	5.0370005	3.4972180	1.0616995
H	5.5728987	2.5625706	0.9332664
H	5.3538120	3.9670834	1.9968014
H	5.3030595	4.1494872	0.2264781
C	3.7473822	-0.2961767	0.4965313
H	3.0862751	-1.0528075	0.9266918
H	4.7690333	-0.6874350	0.5054308
H	3.4552623	-0.1264746	-0.5366064
C	4.3701883	0.9220029	2.5349259
H	4.1940276	1.8529922	3.0630854

H	5.4500797	0.7643189	2.4401546
H	3.9610500	0.1055862	3.1379004
C	0.4097907	0.5537488	1.2306000
C	0.6143867	1.2995245	2.4296660
C	0.6735704	2.0291883	3.4051216
C	-0.9212260	4.5033912	4.1044270
H	-0.9704703	5.2991763	4.8588986
C	-0.6316792	5.1665492	2.7491838
H	0.3031657	5.7300694	2.7487033
H	-1.4324775	5.8635406	2.4824642
H	-0.5698417	4.4195259	1.9542903
C	-2.2941427	3.8142804	4.0689049
H	-2.3020139	2.9867749	3.3550706
H	-3.0701617	4.5204751	3.7569854
H	-2.5894253	3.4182001	5.0421425
C	-0.0368056	2.5463197	6.3419951
H	-0.9950869	2.0765926	6.0924667
C	-0.3112235	3.5688063	7.4582955
H	0.6076013	4.0589599	7.7890274
H	-0.7430194	3.0745851	8.3342864
H	-1.0088977	4.3499224	7.1495397
C	0.8835304	1.4277647	6.8518975
H	1.1041935	0.7003165	6.0703072
H	0.4145896	0.8930073	7.6843368
H	1.8339096	1.8195675	7.2180682
C	2.1250154	4.3384362	4.7665435
H	2.3842516	4.4745254	3.7102580
C	1.9908328	5.7389209	5.3892855
H	1.2350666	6.3481764	4.8915656
H	2.9406540	6.2791511	5.3220588
H	1.7267938	5.6877022	6.4480749
C	3.2904403	3.5814647	5.4235744
H	3.1652110	3.5231282	6.5068651
H	4.2386668	4.0970569	5.2401506
H	3.3886616	2.5606783	5.0507474
Si	-0.4890142	-3.3480733	-4.6885857
N	-1.2320707	-0.9053413	2.3683372
N	-1.1566656	-1.2388534	4.6576832
N	0.6087975	-2.0394132	3.3554300
N	-1.9951171	-1.9956710	0.0278371
N	-3.5929676	-3.2702468	-1.0550311
N	-3.7144402	-0.9489979	-1.2411383
C	-0.5782987	-0.4668136	1.2066185
C	-1.0341159	-0.9769897	-0.0256278
C	-0.5905368	-1.3714790	3.4248279
C	-1.1397386	-2.3294393	5.6303398
H	-0.5026251	-3.1387912	5.2891832
H	-0.7834860	-1.9782773	6.6023935
H	-2.1512738	-2.7275496	5.7442825
C	-2.2649266	-0.3104917	4.8358055
H	-3.2186336	-0.7764109	4.5689929

H	-2.3000253	-0.0142639	5.8859490
H	-2.1138105	0.5661815	4.2145364
C	1.6226906	-1.9705676	4.3901096
H	1.7694735	-2.9366637	4.8860078
H	2.5787649	-1.6746337	3.9473376
H	1.3489980	-1.2309873	5.1356479
C	0.9273872	-2.8915966	2.2224394
H	1.6898295	-2.4528665	1.5736503
H	1.3049084	-3.8492445	2.5941909
H	0.0361781	-3.0812744	1.6301756
C	-3.0593126	-2.0521378	-0.7530405
C	-2.8108457	-4.4736397	-0.8082603
H	-2.9233109	-4.8138677	0.2258895
H	-3.1675101	-5.2605657	-1.4760989
H	-1.7617819	-4.2796311	-1.0051909
C	-5.0370005	-3.4972180	-1.0616995
H	-5.5728987	-2.5625706	-0.9332664
H	-5.3538120	-3.9670834	-1.9968014
H	-5.3030595	-4.1494872	-0.2264781
C	-3.7473822	0.2961767	-0.4965313
H	-3.0862751	1.0528075	-0.9266918
H	-4.7690333	0.6874350	-0.5054308
H	-3.4552623	0.1264746	0.5366064
C	-4.3701883	-0.9220029	-2.5349259
H	-4.1940276	-1.8529922	-3.0630854
H	-5.4500797	-0.7643189	-2.4401546
H	-3.9610500	-0.1055862	-3.1379004
C	-0.4097907	-0.5537488	-1.2306000
C	-0.6143867	-1.2995245	-2.4296660
C	-0.6735704	-2.0291883	-3.4051216
C	0.9212260	-4.5033912	-4.1044270
H	0.9704703	-5.2991763	-4.8588986
C	0.6316792	-5.1665492	-2.7491838
H	-0.3031657	-5.7300694	-2.7487033
H	1.4324775	-5.8635406	-2.4824642
H	0.5698417	-4.4195259	-1.9542903
C	2.2941427	-3.8142804	-4.0689049
H	2.3020139	-2.9867749	-3.3550706
H	3.0701617	-4.5204751	-3.7569854
H	2.5894253	-3.4182001	-5.0421425
C	0.0368056	-2.5463197	-6.3419951
H	0.9950869	-2.0765926	-6.0924667
C	0.3112235	-3.5688063	-7.4582955
H	-0.6076013	-4.0589599	-7.7890274
H	0.7430194	-3.0745851	-8.3342864
H	1.0088977	-4.3499224	-7.1495397
C	-0.8835304	-1.4277647	-6.8518975
H	-1.1041935	-0.7003165	-6.0703072
H	-0.4145896	-0.8930073	-7.6843368
H	-1.8339096	-1.8195675	-7.2180682
C	-2.1250154	-4.3384362	-4.7665435

H -2.3842516 -4.4745254 -3.7102580
C -1.9908328 -5.7389209 -5.3892855
H -1.2350666 -6.3481764 -4.8915656
H -2.9406540 -6.2791511 -5.3220588
H -1.7267938 -5.6877022 -6.4480749
C -3.2904403 -3.5814647 -5.4235744
H -3.1652110 -3.5231282 -6.5068651
H -4.2386668 -4.0970569 -5.2401506
H -3.3886616 -2.5606783 -5.0507474
I -4.4413335 -3.6752108 3.1445463
Cu -2.7286013 -2.3031139 1.9802671

Electronic transitions of [**2a**($CuI)_2$] found in the TD-DFT calculations (B3LYP/def2-TZVP)

Only electronic ttransitions with an intensity of more than 1.10^{-2} km/mol are listed

λ/nm	oscillator strength	leading contribution		
486.4	0.013	$150a_g \rightarrow 150a_u$	HOMO → LUMO	97.4%
453.5	0.127	$149b_2 \rightarrow 150a_u$	HOMO-2 → LUMO	96.1%
367.2	0.104	$147a_g \rightarrow 150a_u$	HOMO-5 → LUMO	79.0%
335.5	0.395	$149a_g \rightarrow 151a_u$	HOMO-2 → LUMO+1	68.1%

Figure S51. Comparison between experimental and simulated electronic excitation spectrum of [**2a**($CuI)_2$]

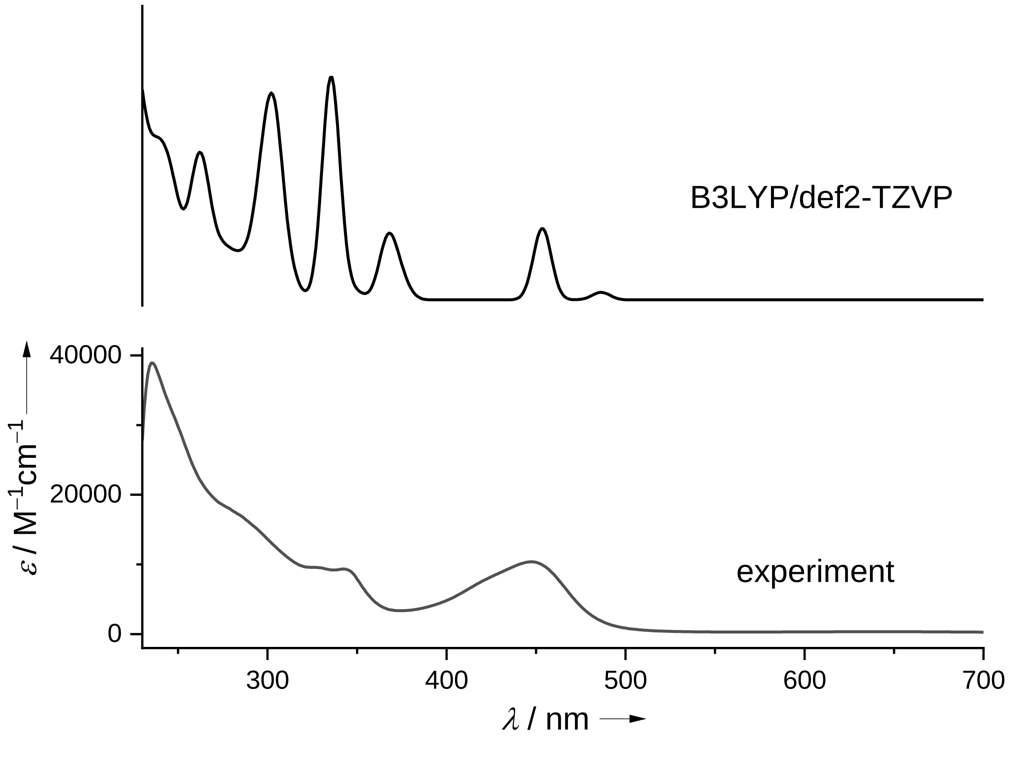

Figure S52. Isodensity plots for the relavant orbitals of [**2a**(CuI)$_2$]. Contour values for the isodensity plots are ±0.02 Bohr$^{(-3/2)}$.

λ = 486.4 nm (97.4% HOMO → LUMO)

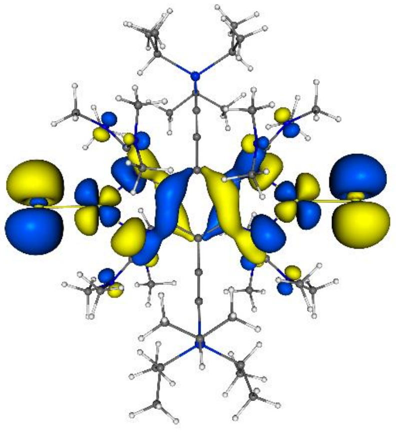

HOMO

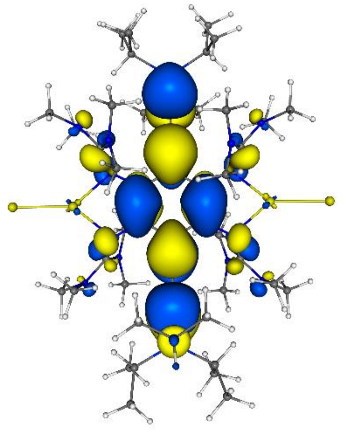

LUMO

λ = 453.5 nm (96.1% HOMO-2 → LUMO)

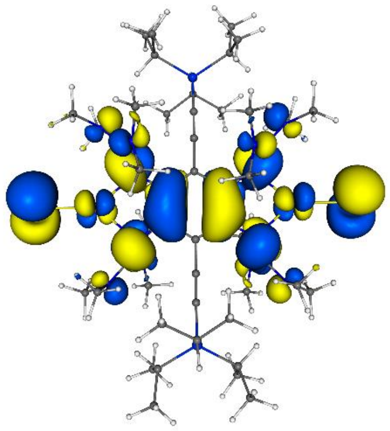

HOMO-2

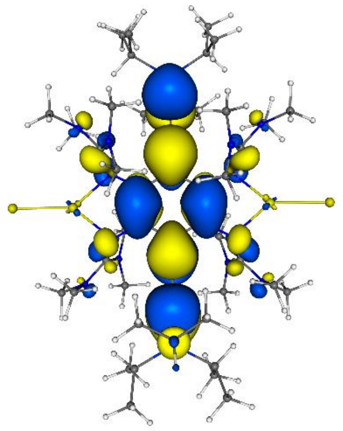

LUMO

λ = 367.2 nm (79.0% HOMO-5 → LUMO)

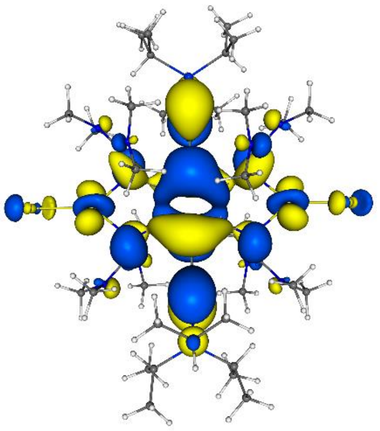

HOMO-5

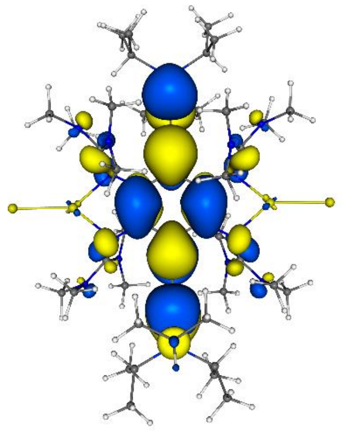

LUMO

λ = 335.5 nm (68.1% HOMO-2 → LUMO+1)

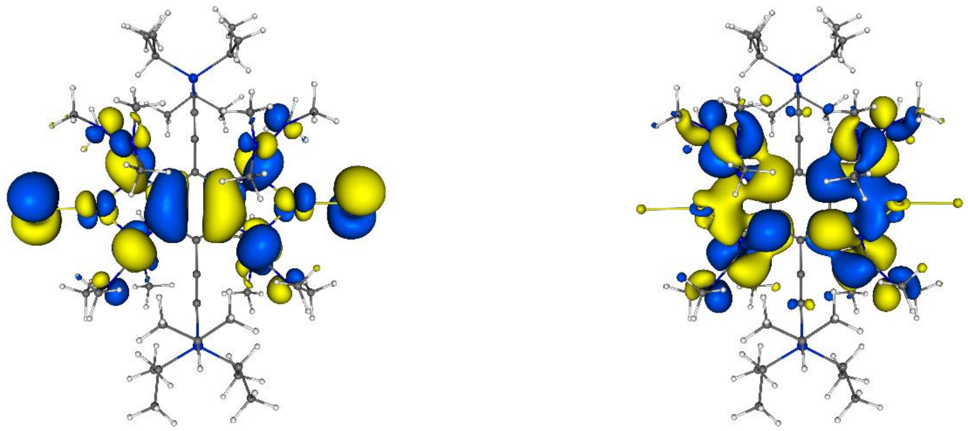

HOMO-2 **LUMO+1**
